# Meeting abstracts from the International Stillbirth Alliance Conference 2017: Cork, Ireland. September 22-24, 2017

**DOI:** 10.1186/s12884-017-1457-7

**Published:** 2017-09-21

## Plenary Orals

### PL1 Prototype “Belly Band” wearable monitor for continuous monitoring of fetal ECG and fetal movement during the third trimester

#### Ajay Iyer, Ali Carlile, Bruce Olney, Paul Allen, Sean Kerman, Ryan Workman, Zack Bomsta, Kurt Workman, Kenneth Ward

##### Owlet Baby Care, Lehi, UT, USA

###### **Correspondence:** Ajay Iyer; Kenneth Ward

Intrauterine fetal demise due to utero-placental insufficiency usually occurs between obstetric evaluations when the gestation is not being actively monitored. Rapid advances in the development of “wearable” physiologic monitors linked to smart phone apps allow us to envision novel methods for evaluating the health of a pregnancy, potentially allowing intervention before fetal death or damage from asphyxia occur. In this report, we describe efforts to develop a fetal ECG monitor that can be worn throughout the day to confirm fetal wellbeing.

A wearable fabric band worn around the maternal abdomen was developed with various novel electrode materials and using various electrode placement patterns. After optimization, “in-laboratory” and overnight “at-home” fetal ECG recordings were collected. The raw signal was processed to subtract interference from maternal ECG, movement, and breathing.

Low-cost breathable materials and miniaturized hardware were selected to allow a comfortable fit. To date over 125 hours of fetal ECG recordings have been obtained on 38 gravidae at gestational ages from 20 weeks’ to term (mean 27 weeks’). Remarkably, over 86.7% of the overnight and 47.6% of the “in-laboratory” records tracked the fetal ECG for more than 50% of the session. Mean fetal heart rate observed was 135 (range 112-186); mean maternal heart rate was 82 (range 55-111). As with prior efforts to obtain fetal ECGs, the best fetal ECG readings were obtained between 20-24 and 36-40 weeks’ gestation.

Progress is reported on development of wearable fetal monitoring hardware. The “belly band” under development can connect wirelessly to a smart-phone app that monitors, analyzes, and transmits the maternal and fetal heart rates. It is hoped that improved monitoring can help to reduce preventable fetal damage and stillbirths.

### PL2 Supine going-to-sleep position is a major risk factor for term stillbirth: findings from the New Zealand multicentre stillbirth case-control study

#### Minglan Li^1^, Lesley ME McCowan^1^, John MD Thompson^1,2^, Robin S Cronin^1^, Ngaire Anderson^1^, Tomasina Stacey^3^, Peter Stone^1^, Beverley A Lawton^4^, Alec J Ekeroma^1^, Edwin A Mitchell^2^

##### ^1^Department Of Obstetrics And Gynaecology, University Of Auckland, Auckland, New Zealand; ^2^Department of Paediatrics: Child and Youth Health, University of Auckland, Auckland, New Zealand; ^3^School of Healthcare, University of Leeds, Leeds, UK; ^4^Women’s Health Research Centre, University of Otago, Wellington, New Zealand

###### **Correspondence:** Minglan Li

Stillbirth is a major health burden and is twice as common as neonatal death in developed countries. Identifying potentially modifiable risk factors for stillbirth may reduce the prevalence of this tragic condition. Previous studies have reported that going-to-sleep supine may increase late stillbirth (≥28 weeks’) risk by more than two-fold compared to left side going-to-sleep position. However, it is not clear if this risk differs between preterm (≥28-36 weeks’) and term (≥37 weeks’) gestations.

A multicentre case-control study was conducted in seven New Zealand health regions, between February 2012 and December 2015. Cases (*n* = 164) were women with singleton pregnancies and late stillbirth, without congenital abnormality. Controls (n = 569) were women with on-going singleton pregnancies, frequency matched for health region and gestation. The going-to-sleep position was the position on the night before the stillbirth was thought to have occurred or the night before interview for controls. Multivariable logistic regressions were conducted in all pregnancies and by term and preterm, and adjusted for known confounders.

The overall risk of supine going-to-sleep position is 3.67 (adjusted odds ratio (aOR), 95% confidence interval (CI) 1.74-7.78). In term pregnancies, there was an increase in stillbirth risk in women who went to sleep supine (aOR 10.26, 95% CI 3.01-35.04) compared to women who went to sleep on their left. In preterm pregnancies, the aOR for late stillbirth in women who went to sleep supine was 3.12 (95% CI 0.97-10.05) compared to those who went to sleep on their left. The prevalence of supine going-to-sleep position was 2.8% in term controls, and 4.4% in preterm controls.

The magnitude of risk associated with supine going-to-sleep position may be greater for term pregnancies compared with those between 28 and 36 weeks’. The prevalence of supine going-to-sleep position is lower in term gestation than in preterm gestation.

Ethical approval for the New Zealand multicentre stillbirth case-control study was obtained from the Northern “X” Regional Ethics Committee: NTX/06/05/054. Written informed consent was obtained by all study participants.

### PL3 The public awareness of stillbirth: a population study

#### Daniel Nuzum^1^, Sarah Meaney^2^, Keelin O’Donoghue^1,3^

##### ^1^Department of Obstetrics and Gynaecology, University College Cork, Cork, Ireland; ^2^National Perinatal Epidemiology Centre, University College Cork, Cork, Ireland; ^3^Irish Centre for Fetal and Neonatal Translational Research (INFANT), University College Cork, Cork, Ireland

###### **Correspondence:** Daniel Nuzum

There is renewed global focus to reduce the incidence of stillbirth. Studies have identified modifiable and non-modifiable risk factors and causes of stillbirth. The aim of this study was to evaluate the general population’s understanding of stillbirth incidence, causes and risk factors.

A cross sectional survey of Irish adults was facilitated by a professional telephone polling company. Participants were contacted using random digit dialling. To ensure a representative sample of the population, fieldwork was monitored daily in terms of age, gender, social class and region of respondents.

There were 999 participants in the study, of whom 53% (n = 510) were female. A minority, 17% (n = 174) were aware of the correct incidence of stillbirth in Ireland. Females and those aged under 45 believed stillbirth to be more common with 23% (n = 230) believing it occurred in 1/100 pregnancies and 24% (n = 244) in 1/50 pregnancies. The majority of respondents 70% (n = 693) stated that stillbirth cannot be prevented. Over half of respondents 53% (n = 528) responded that stillbirth was due to a problem with the baby, while 31% (n = 309) agreed that stillbirth was due to care provided to the mother. One in three, 29% (n = 285) stated that the cause of stillbirth is usually unexplained, however 79% (n = 792) agreed that all stillbirths should be medically investigated. Women, were more likely than men (82% v 76.4%; p = 0.043) to state that all stillbirths should be investigated. Over half of respondents, 56% (n = 556) were unable to identify any risk factors for stillbirth; 28% (n = 275) identified alcohol, 22% (n = 222) smoking, 16% (n = 158) substance abuse and <1% (n = 2) identified reduced fetal movements.

These findings highlight a lack of knowledge about incidence, causes and investigation of stillbirth. The findings demonstrate the need for improved education and awareness about stillbirth risk factors as part of antenatal education.

Ethical approval for the study was granted by Clinical Research Ethics Committee of the Cork Teaching Hospitals (Reference; ECM 4 (k) 06/12/16).

### PL4 Development and initial validation of ‘Perinatal Bereavement Care Confidence Scale (PBCCS)’

#### Felicity Agwu Kalu^1^, Barbara Coughlan^2^, Philip Larkin^3^

##### ^1^Rotunda Hospital, Dublin, Ireland; ^2^University College Dublin, Dublin, Ireland; ^3^University College Dublin, Dublin, Ireland

###### **Correspondence:** Felicity Agwu Kalu

Evaluating the levels of confidence and psychosocial factors that impact on midwives’ and nurses’ confidence to provide perinatal bereavement care to parents who have experienced a perinatal loss is vital for assessing their abilities for providing effective care to the parents. Due to the shortage of questionnaires specifically developed and designed to measure the level of midwives’ and nurses’ confidence (bereavement care knowledge and skills) and the psychosocial factors that impact on their confidence (self-awareness and organisational support) to provide bereavement care to parents who experienced a perinatal loss, a valid and reliable questionnaire was needed.

The purpose of the study was to develop a valid and reliable perinatal bereavement care confidence scale (PBCCS).

The PBCCS was developed in 4 phases. First, 42 questions were formulated from extensive literature review. This was followed by expert panel review (n = 6). Then pilot study was conducted and included cognitive pre-testing interviews (n = 10) and test-retest reliability assessment with midwives (n = 26). Finally, factor analysis was conducted to examine the factor structure of the PBCCS with midwives and nurses (n = 277). Item reduction was done by removing those items with poor loading of less than 0.5. Internal consistency reliability measurement was assessed. Ethical approval of the study was obtained from four research sites.

The final questionnaire had 41 items. Bereavement care knowledge had 15 items. Bereavement care skills had 9 items. Self-awareness had 8 items. Organisational support had 11 items. The internal consistency reliabilities ranged from 0.797 to 0.855.

The PBCCS has adequate psychometric properties to identify the levels of confidence of midwives and nurses as well as the psychosocial factors that impact on their confidence to provide effective care to parents who have experienced a perinatal loss.

An ethical approval for the study was granted by four research sites namely; The Health Service Executive Dublin North East, Rotunda Hospital, National Maternity Hospital, and Coombe Women & Infants University Hospital Research Ethics Committees (REC-2013-009 & REC-2013-018). Written informed consent was obtained by all study participants with the exception of participants who completed anonymised survey used for the factor analysis. In that case, it was clearly stated on each of the participant’s information leaflet that, by voluntarily completing and returning the questionnaire, the participant was consenting to participate in the research.

### PL5 Global reporting of the causes of stillbirth: a systematic review

#### Hanna Reinebrant^1^, Michael Coory^1^, Susannah Hopkins Leisher^1^, Sarah Henry^1^, Rohan Lourie^1^, Aleena M Wojcieszek^1^, Hannah Blencowe^1^, Vicki Flenady^1,2^

##### ^1^Centre of Research Excellence in Stillbirth, Mater Research Institute - The University of Queensland (MRI-UQ), Brisbane. Australia; ^2^on behalf of the International Stillbirth Alliance Collaborative for Improving Classification of Perinatal Deaths, Bristol, UK

###### **Correspondence:** Hanna Reinebrant

Stillbirth is a global health problem. Understanding the causes globally is key to prevention. Numerous disparate classification systems in use makes understanding causes difficult. The primary objective of this study was to comprehensively summarise the causes of stillbirth reported globally to identify areas for prevention and improvement in data quality.

We undertook a systematic literature review for studies reporting causes of stillbirth over the period 1 January 2009 to 31 December 2016. Study selection, quality assessment and data extraction on causes and characteristics of systems used, stillbirth investigations undertaken and data sources was undertaken independently by two authors. Reported causes were mapped into clinically relevant groupings. A pooled analysis (including 95% Prediction intervals –PI) of major categories for a selection of country representative studies was performed by country setting: High, Middle, and Low income countries (HIC, MIC, LIC).

89 studies reporting 489,305 stillbirths were included with a total of 22 different classification systems. 897 unique causes were identified and mapped to 13 major and 45 minor categories of which 7 major categories were represented in over 60% of studies. Pooled analysis of 49 studies (461,166 stillbirths) showed wide variation in the major categories. Unexplained stillbirth was the top category across all country settings with a pooled estimate of 30-40% of stillbirths. The leading category in LIC of Hypoxic peripartum death (16.8%), Other unspecified condition in MIC (17.6%) and Placenta in HIC (15.5%). Suboptimal data sources (retrospective use of death certificate data) and investigation of stillbirth (low autopsy and placental pathology rates) were evident for the vast majority of the studies.

Efforts to improve consistency and accuracy in global reporting of stillbirth is a priority to inform effective prevention.

## Break out orals

### B1 Improving quality of care in pregnancies after stillbirth- an improvement science project in two UK maternity hospitals

#### Louise Stephens^1^, Christine Navin^2^, Suzanne Thomas^1^, Sreebala Sripada^2^, Elaine Church^2^, Clare Tower^1,3^, Alexander Heazell^1,3^

##### ^1^St Mary’s Hospital, Central Manchester University Hospitals NHS Foundation Trust, Manchester Academic Health Science Centre, Manchester, M13 9WL, Manchester, UK; ^2^University Hospitals of South Manchester, Manchester Academic Health Science Centre, M23 9LT, Manchester, UK; ^3^Maternal and Fetal Health Research Centre, School of Medical Sciences, Faculty of Biological, Medical and Human Sciences, University of Manchester, M13 9WL, Manchester, UK

###### **Correspondence:** Louise Stephens

Pregnancies after stillbirth have increased risk of stillbirth and other adverse pregnancy outcomes which alter mothers’ needs for maternity care. In response to the additional need for support and screening we developed a specialist clinic for families at St Mary’s Hospital, a Tertiary Maternity Unit, in 2013. In response to clinic demand, a second site in University Hospitals South Manchester, a large secondary maternity unit, was set up in 2016.

We aimed to improve access to specialist care, improve patient experience and improve pregnancy outcome for parents who have experienced a stillbirth in a previous pregnancy by May 2017.

We developed a measurement strategy which recorded process measures of care including: the number of women attending the Rainbow Clinic, the number of women who did not attend and their reasons for not attending. Patient experience was measured using a bespoke Patient Experience Questionnaire, collecting both qualitative and quantitative responses at the final Rainbow Clinic appointment. The outcome of pregnancies including gestation at birth, mode of delivery and NICU admissions was recorded.

**Fig. 1 (abstract B1).**
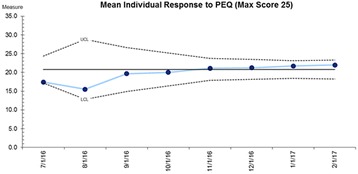
Mean individual response to PEQ (Max Score 25)

In 2016, 160 women were seen in Rainbow Clinics on both sites, an increase of 12.5%. Patient experience improved on both sites over the duration of the project (Fig. 1). All families would recommend our service to friends and family. Qualitative data indicates positive patient experience with relevant recommendations for improvement.

There were no subsequent stillbirths, a reduced preterm birth rate from 21 to 13% and admissions to Neonatal Intensive Care Unit reducing from 17.5% to 8.5%.

Attendance at a specialist clinic in subsequent pregnancy has a positive effect on patient experience and pregnancy outcomes. Provision of the model of care on a second site has provided a choice of settings for women and their families with no negative impact on patient experience.

### B2 The social and economic value of a clinical service for care in pregnancy after perinatal death

#### Suzanne Thomas^1,3^, Louise Stephens^1,3^, Christine Navin^2^, Anthony Harrison^4^, Alexander Heazell^1,3^

##### ^1^Central Manchester Foundation Trust, Manchester, UK; ^2^University Hospitals of South Manchester, Manchester, UK; ^3^Maternal & Fetal Health Research Centre, Manchester, UK; ^4^The Alliance Manchester Business School, Manchester, UK

###### **Correspondence:** Suzanne Thomas

The Rainbow Clinic at St Mary’s Hospital, Manchester was established in 2013 to provide individualised care to women and their families in pregnancies following a perinatal death. Although the clinic is highly valued by the families it has cared for, the service has not been evaluated in terms of its wider economic and social impact. In 2015 The Rainbow Clinic worked with Alliance Manchester Business School (AMBS) to determine the social and economic value of the Rainbow Clinic.

A Social Return on Investment (SROI) framework was used to capture a monetised value of a range of outcomes whether they already have a financial value or not. SROI analysis produces a narrative of how a service creates and loses value in the course of making change. The six stage process results in the calculation of a ratio that states how much social value is created for every £1 of investment. A mixed methods approach was used to collect data required to calculate SROI. Interviews were conducted with stakeholders and service users experiences were collected via questionnaires.

For every £1 invested the Rainbow Clinic delivers £1.78 of social value. The quantified benefits of the Rainbow Clinic across all stakeholder groups were identified as: reduced anxiety and stress for mothers-to-be, reduced outlays for mothers-to-be through the avoidance of funeral costs, avoidance of stillbirth, value of a new life, reduced health and post-mortem expenses, recognition and donations for charitable funders, education and enhanced skill base amongst Clinic staff and wider non-specialist antenatal staff and increased job satisfaction.

The findings suggest that the investment in Rainbow Clinic generates social and economic value. The clinic also decreases costs for wider health services. SROI analysis is a valuable means to assess the impact of services for parents who have experienced a perinatal death.

### B3 Journey of loss: the lived experiences of couples’ journeys from stillbirth to subsequent pregnancy

#### Margaret Murphy^1,2,4^, Eileen Savage^1^, Keelin O Donoghue^1,2,3^, Patricia Leahy-Warren^1^

##### ^1^School of Nursing and Midwifery, University College Cork, Cork, Ireland; ^2^Pregnancy Loss Research Group, Cork University Maternity Hospital, Cork, Ireland; ^3^Infant Research Centre, Cork University Maternity Hospital, Cork, Ireland; ^4^International Stillbirth Alliance, Bristol, UK

###### **Correspondence:** Margaret Murphy

Stillbirth affects 2.6 million families globally each year and can have significant adverse consequences for mothers and fathers, individually and also as a couple. A high percentage of couples proceed to a subsequent pregnancy within a short timeframe of their index loss and are trying to bond with a new baby while still actively grieving their lost baby. Little is known about how mothers and fathers, as a couple, experience pregnancy after loss. Previous research has predominantly focused on mothers and none has interviewed couples together to explore this experience. The aim of this qualitative study was to explore the experiences of couples, in pregnancy after stillbirth.

An Interpretive Phenomenological Analysis (IPA) methodology was employed and eight heterosexual couples (16 participants), in an immediate pregnancy after loss, agreed to participate. Following ethical approval, in-depth dyadic interviews lasting 90-120 minutes were conducted. Intensive data analysis using strict IPA principles was performed.

A superordinate theme that emerged from the data was ‘Journey of Loss’ in which couples made sense of their subsequent pregnancy through the lens of their previous pregnancy journey. Participants shared experiences of coping with unexpected loss as a couple, the importance of grieving the lost baby and acknowledging that baby’s place in their family. The challenges couples experienced trying to achieve a pregnancy after loss were also explored. Similarities and differences both between mother’s and father’s experiences and within couples emerged.

This study offers a unique insight into couples’ experiences of pregnancy after loss as it is the first study to explore the dynamics of couples as a dyad. It provides insights into how mothers and fathers make sense of their experiences of loss and subsequent pregnancy. It offers recommendations on how couples can be supported through the journey of loss and a subsequent pregnancy.

Ethical approval for the study was granted by Clinical Research Ethics Committee of the Cork Teaching Hospitals (Reference; ECM 4(d) 14/04/15). Written informed consent was obtained by all study participants.

### B4 Care practices after stillbirth: an international perspective

#### Fran Boyle^1,2,3^, Dell Horey^1,3,4^, David Ellwood^1,3,5^, Aleena Wojcieszek^1,2,3^, Katy Gold^3,6^, Dimitrios Siassakos^3,7^, Jan Jaap Erwich^3,8^, Jessica Ruidiaz^3,9^, Jillian Cassidy^3,10^, Paul Cassidy^3,10^, Susannah Leisher^2,3^, Lynn Farrales^3,11^, Claire Storey^3^, Margaret Murphy^3,12^, Mairie Cregan^3,13^, Claudia Ravaldi^3,14^, Alfredo Vannacci^3,14^, Victoria Bowring^3,15^, Jose Belizan^3,16^, Ingela Radestad^3,17^, Mechthild Gross^3,18^, Alex Heazell^3,19^, Vicki Flenady^1,2,3^

##### ^1^Centre of Research Excellence in Stillbirth, Brisbane, Australia; ^2^Mater Research Institute - University Of Queensland, Brisbane, Australia; ^3^International Stillbirth Alliance, Bristol, UK; ^4^College of Science, Health & Engineering, La Trobe University, Melbourne, Australia; ^5^Griffith University and Gold Coast University Hospital, Gold Coast, Australia; ^6^Department of Family Medicine and Department of Obstetrics & Gynecology University of Michigan, Ann Arbor, MI, USA; ^7^Academic Centre for Women’s Health, University of Bristol, Bristol & Southmead Hospital, Bristol, UK; ^8^Department of Obstetrics, University of Groningen, University Medical Centre, Groningen, The Netherlands; ^9^Era en Abril, Buenos Aires, Argentina; ^10^Umamanita, Girona, Spain; ^11^University of British Columbia and Still Life Canada, Vancouver, BC, Canada; ^12^School of Nursing and Midwifery, University College Cork, Cork, Ireland; ^13^Feileacain, Cork, Ireland; ^14^CiaoLapo Onlus, Prato, Italy; ^15^Stillbirth Foundation Australia, Annandale, Australia; ^16^Institute for Clinical Effectiveness and Health Policy, Buenos Aires, Argentina; ^17^Sophiahemmet Hogskola, Stockholm, Sweden; ^18^Hannover Medical School, Hannover, Germany; ^19^Institute of Human Development, Faculty of Medical and Human Sciences, The University of Manchester and St Mary’s Hospital, Central Manchester University Hospitals, NHS Foundation Trust, Manchester Academic Health Science Centre, Manchester, UK

###### **Correspondence:** Fran Boyle

Care after stillbirth has important implications for women and families. Care practices valued by parents and contributing to better outcomes have been identified, but implementation appears inconsistent. This international perspective highlights variations in care practices after stillbirth across selected high- (HIC) and middle-income countries (MIC).

Data were from a large multi-country survey of parents of stillborn infants conducted between December 2014 and February 2015. An online questionnaire, distributed primarily through ISA member organisations, covered a range of topics, including whether seven care practices were offered. Mothers’ responses (yes/no) to these seven items were compared for regions and countries.

More than 3000 mothers from 22 HICs and over 600 mothers from 10 MICs responded. Italy, (720); UK, (576); Australia & New Zealand, (460); US, (402) and Argentina, (423) and Mexico, (168) were most strongly represented.

Stillbirth was more likely within the last five years for those responding from MIC. Women from MIC were less likely to be offered recommended care than women from HIC, including opportunity to: spend time with baby (45 vs 81%); see and hold baby (51 vs 85%); have family and friends meet baby (56 vs 74%); create memories (37 vs 70%); name baby (62 vs 88%); take baby home (15 vs 19%); and have funeral or other service (58 vs 84%). Differences were also observed across HICs: e.g., in Ireland and New Zealand more than half had the opportunity to take their baby home but this was rare in other countries.

Striking differences were apparent. Despite the complexities of cross-country data comparisons, considerable variation between HIC and MIC and between some HIC was evident. Providing respectful and supportive care after stillbirth is challenging but building health system capacity to respond to families’ needs is an urgent priority, particularly in lower-resource settings.

Ethics approval was granted by the Mater Health Services Human Research Ethics Committee on 29th November 2013 (Ref #HREC/13/MHS/121) and by the University of British Columbia Office of Research Services, Behavioural Research Ethics Board on 22nd December 2014 (Ref #H14-02784). Completion of the anonymous online survey indicated consent to participate in the study.

### B5 Learning from deaths: healthcare professionals’ views on parental involvement in the perinatal mortality review process (The PARENTS 2 Study)

#### Danya Bakhbakhi^1,2^, Christy Burden^2^, Claire Storey^1^, Alexander Heazell^3^, Mary Lynch^1^, Laura Timlin^1^, Dimitrios Siassakos^2^

##### ^1^North Bristol Nhs Trust, Bristol, UK; ^2^University of Bristol, Bristol, UK; ^3^University of Manchester, Manchester, UK

###### **Correspondence:** Danya Bakhbakhi

There is a national priority to learn from perinatal deaths when they occur. The United Kingdom (UK) government has recommended that these incidents should be subject to a standardised process, which incorporates feedback from parents and families within a multidisciplinary review. The PARENTS1 project investigated parental opinion on the perinatal mortality review (PNMR) process and concluded that parents were in favour of a system where they could give and receive feedback as part of the review. Following on, we assessed healthcare professionals’ views on parental involvement in the PNMR as part of a larger project (PARENTS2) which aims to integrate of perceptions of parents, staff, and stakeholders into the design and pilot of a parental involvement in the PNMR.

We purposively sampled a range of healthcare professionals including obstetricians, midwives, neonatologists, nursing staff, and hospital chaplains, to participate in a focus group in Bristol, UK. We conducted a semi-structured group discussion using a pre-designed topic guide. The data were analysed with an inductive thematic analysis technique.

All healthcare professionals saw the benefit of receiving feedback from parents. Emerging themes included: parallels to parent focus group – consideration of emotional and clinical care, with flexibility and an optional process – and the importance of training and supporting staff, as well as the importance of multi-disciplinary follow-up consultation. Other themes were the structural/system adaptations required to the current PNMR process including ground rules, role of facilitators, and a standardised format, and coping strategies for emotional impact, conflict, complaints and litigation.

Parental involvement in perinatal mortality reviews is considered desirable by staff. They have made recommendations as to how it might be undertaken, so that it is feasible. The next step is to obtain key stakeholders’ views and reach a consensus as to how parental involvement in the process should be implemented in hospitals across the UK, and worldwide.

Approval for the study was granted by the Health Research Authority (Reference 220468). Written informed consent was obtained by all study participants.

### B6 Specialist photographic training increases midwives’ confidence in initiating and undertaking memento photography following perinatal loss

#### Elizabeth Bailey^1^, Rachel Hayden^2^, Sam Collinge^3^

##### ^1^University Hospitals Coventry & Warwickshire NHS Trust, & Coventry University, Coventry, UK; ^2^Gifts of Remembrance, Hinckley, UK; ^3^University Hospitals Coventry & Warwickshire NHS Trust, & Coventry University, Coventry, UK

###### **Correspondence:** Elizabeth Bailey; Rachel Hayden

This evaluation was developed to measure midwives’ confidence before and after specialist training which aimed to support midwives in providing meaningful photographic mementoes for families experiencing perinatal loss. (Creating memories can be of vital psychological importance in helping families come to terms with the death of a baby. Parents often cherish these photographs for the rest of their lives. As we only have ‘one chance to get it right’ (Downe et al 2103), it is desirable that midwives receive adequate training in introducing memento photography and memory-making activities).

Two training days took place in 2015. Before attending, those registered had been invited via email to complete an anonymous, online pre-training questionnaire. The post-training survey with similar questions to allow before and after comparison was sent out approx. 14 weeks after the training session. The training session focused on methods of empowerment along with how to approach families on the sensitive subject of memory making and skills in documentary photography in this setting.

The pre-training survey was completed by 31 training attenders and the post training by 22 respondents. There was a notable increase in confidence regarding memento photography among midwives post-training compared to pre-training. Midwives’ confidence increased in suggesting poses/positions to families (0% post-training vs 48% pre-training having ‘no confidence at all’). This increase in confidence was echoed in using props in photographs (such as teddies, blankets, toys); in arranging subjects and items in shot for photographic effect; and in initiating a discussion with parents on the value of mementoes (73% post-training vs 36% pre-training being ‘quite confident’).

Providing care to bereaved parents is a challenging component of midwifery practice with midwives reporting low confidence prior to specialist training on discussing and conducting memento photography with parents. This training was effective in increasing midwives’ confidence.

The photography training provided to healthcare staff formed part of their ongoing professional development and therefore ethics approval for this aspect was not required. The evaluation of the study however was approved by Coventry University Ethics Committee (reference P33015).

### B7 Failure of placental morphological adaptation may be implicated in stillbirth in older mothers: a case-control study

#### Michael Cocker^1,2^, Samantha C. Lean^1,2^, Rebecca L. Jones^1,2^, Gauri Batra^1,3^, John M. D. Thompson^4^, Alexander Heazell^1,2^

##### ^1^Divison of Developmental Biology and Medicine, School of Medical Sciences Faculty of Biology, Medicine & Health, University of Manchester, Manchester, UK; ^2^St. Mary’s Hospital, Central Manchester University Hospitals NHS Foundation Trust, Manchester, UK; ^3^Department of Histopathology, Royal Manchester Children’s Hospital, Central Manchester University Hospitals NHS Foundation Trust, Manchester, UK; ^4^Department of Paediatrics, University of Auckland, Auckland, New Zealand

###### **Correspondence:** Elizabeth Bailey; Rachel Hayden

Advanced maternal age (AMA) ≥35 years is a recognized risk factor for stillbirth, although the underlying aetiology is incompletely understood and appears particularly heightened in later gestations. This study aimed to examine placental factors in the aetiology of stillbirth in AMA mothers, and if these differ from stillbirths in younger women. We also aimed to investigate whether placental differences exist in stillbirth compared to live births in AMA mothers.

A retrospective case-control study was conducted examining differences in placenta weight, fetal:placental weight ratio and histopathological lesions between non-anomalous, singleton stillbirths in AMA mothers (n = 35) and stillbirths in women <35 years (n = 70) identified from the stillbirth database of a tertiary obstetric unit (2009-2015). AMA live births (n = 70) and live births to women <35 years (n = 27) were included from the Manchester Advanced Maternal Age Study (MAMAS). Subjects were matched for gestational age, ethnicity, pre-existing medical conditions, smoking status and fetal gender (Table 1).

Median placental weight centile corrected for gestation was higher (Mann-Whitney z = 2.90, p = 0.004), and median corrected F:PWR centile was lower (Mann-Whitney z = -2.52, p = 0.012) in AMA live birth compared to live births to younger women. No similar changes were observed between stillbirth groups. No significant differences were seen in ReCoDe classifications or placental histopathological findings between AMA stillbirths and stillbirths to women <35 years.

AMA live birth is associated with less efficient but larger placentas. The failure to develop the adaptive morphological mechanism seen in AMA live births may by implicated in the aetiology of stillbirth in older mothers. The potential impact of antenatal surveillance in this population is poorly understood and warrants further investigation

Ethical approval for the study was granted by NRES Committee North West (Reference; 12/NW/0015). Written informed consent was obtained by all study participants

**Table 1 (abstract B7).** Study group demographics, placental characteristics & placental histology results

		**AMA stillbirth** ***n*** **= 35**	**Stillbirth control group** ***n*** **= 70**	**AMA livebirth** ***n*** **= 70**	**Livebirth control group** ***n*** **= 27**
**Matching criteria**					
Gestational age (days)	Median (IQ range)	237 (199-266)	237.5 (192.5-263.8)	275 (273-278)	279 (272-285)
Pre-existing medical conditions n = (%)	VTE/thrombophilia	4 (11.4)	1 (1.4)	0 (0)	0 (0)
	Hypothyroidism	2 (5.7)	0 (0)	1 (1.4)	0 (0)
	Hypertensive disease	3 (8.6)	2 (2.9)	0 (0)	0 (0)
	Diabetes mellitus	1 (2.9)	3 (4.3)	0 (0)	0 (0)
	Asthma	1 (2.9)	5 (7.1)	0 (0)	0 (0)
	Epilepsy	0 (0)	2 (2.9)	1 (1.4)	0 (0)
Ethnicity n = (%)	White	17 (48.6)	39 (55.7)	57 (81.4)	20 (74)
	African & Caribbean	9 (25.7)	12 (17.1)	3 (4.3)	2 (7)
	Mixed race	1 (2.9)	3 (4.3)	2 (2.9)	1 (4)
	Asian	6 (17.1)	14 (20.0)	6 (8.6)	3 (11)
	Other	2 (5.7)	2 (2.9)	2 (2.9)	1 (4)
Fetal gender n = (%)	Male	21 (60.0)	36 (51.4)	34 (48.6)	15 (56)
	Female	14 (40.0)	34 (48.6)	36 (51.4)	12 (44)
Smoker during pregnancy n = (%)	Yes	7 (18)	16 (22.9)	1(1.4)	3(11)
**Placental characteristics**					
Median placental weight (g) (IQ range)		297 (187.8-387.5)	298.5 (196.5-388)	538 (463.5-602.5)	478 (406.5-530.5)
Median F:PWR (IQ range)		5.5 (4.1-7.2)	5.6 (4.1-7.3)	6.5 (5.7-7.2)	7.2 (6.4-7.8)
**Turowski placental histological classification n = (%)**					
Normal morphology		3 (8.6)	6 (8.6)	No data	
Chorioamnionitis		5 (14.3)	10 (12.8)		
Villitis/intervillositis		10 (28.6)	13 (14.3)		
	Villitis	4 (11.4)	4 (5.7)		
	Intervillositis	6 (17.1)	6 (8.6)		
Maternal circulatory disorders		18 (51.4)	41 (57.1)		
	Cotyledon infarct	9 (25.7)	11 (15.7)		
	Intervillous thrombus	0 (0)	2 (2.9)		
	Abruption	1 (2.9)	5 (7.1)		
	Maternal malperfusion	12 (34.3)	25 (35.7)		
Fetal circulatory disorders		2 (5.7)	7 (10.0)		
Delayed villous maturation		10 (28.6)	13 (18.6)		
**ReCoDe classification n = (%)**					
A2.2		4 (11.4)	4 (5.7)	No data	
A3		0 (0)	1 (1.4)		
A7		12 (34.3)	40 (57.1)		
A8		1 (2.9)	0 (0)		
B2		2 (5.7)	0 (0)		
C1		2 (5.7)	4 (5.7)		
C4		4 (11.4)	7 (10)		
F1		2 (5.7)	2 (2.9)		
F4		3 (8.6)	0 (0)		
F8		0 (0)	1 (1.4)		
G1		0 (0)	1 (1.4)		
I1		5 (14.3)	10 (14.3)		

.

### B8 Targeted for identification of cardiac variants in unexplained intrauterine fetal death – a retrospective pilot study

#### Dana Muin^1^, Martina Kollmann^2^, Gregor Hörmann^1^, Herbert Kiss^1^, Thomas Schwarzbraun^2^

##### ^1^Medical University Of Vienna, Vienna, Austria; ^2^Medical University of Graz, Graz, Austria

###### **Correspondence:** Dana Muin

Congenital heart disease associated with genetic anomalies contributes to intrauterine fetal death (IUFD) in up to 27%, of which approximately 75% of the cases had been prenatally diagnosed. However, structural cardiac disorders (e.g. hypertrophic and dilative cardiomyopathy) may also affect areas critical for electrical transmission, well before structural changes appear in the individual. Yet, without those morphological anomalies detected by ultrasound or post-mortem autopsy, those potential pathomechanisms get easily missed and many cases of fetal demise falsely concluded as “unexplained”.

Clinical exome sequencing and filtering for 122 genes with a known cardiac phenotype on post-mortem samples of 16 singleton fetuses after unexplained IUFD between gestational weeks 23 + 2 and 40 + 5 to analyse the spectrum and frequency of putative pathogenic variants.

In total, twelve (75%) male and four (25%) female fetuses were analysed. In 14 (87.5%) samples, 33 variants were detected in 22 genes by sequencing. Two (12.5%) male Caucasian fetuses elicited no relevant variants in any of the targeted cardiac genes. Potential cardio-pathogenic variants were found in 13 (81.3%) cases, four (30.7%) of which harboured two or more putative variants. Ten (30.3%) variants were detected in seven arrhythmogenic-susceptibility genes and 21 (63,6%) variants were found in 17 genes coding proteins primarily associated with cardiac morphology; two (6.1%) variants were found in a single gene coding for both heart rate and morphology. No correlation was observed with fetal gender, gestational age, medical and family history.

Cardio-genetic pathologies might be a potentially underexplored etiological factor in unexplained IUFD and should be considered further in fetal post-mortem examinations.

Ethical approval for the study was granted by Medical Research Ethics Committee of the Medical University of Vienna, Austria (Reference Number 1852/2016). Written informed consent was obtained by all study participants.

### B9 Recruitment barriers and participant feedback in a New Zealand stillbirth study

#### Robin Cronin^1^, Minglan Li^1^, Billie Bradford^1^, Vicki Culling^2^, John Michael David Thompson^3^, Edwin Mitchell^3^, Lesley Margaret Elizabeth McCowan^1^

##### ^1^University Of Auckland, Auckland, New Zealand; ^2^Vicki Culling Associates, Wellington, New Zealand; ^3^Department of Paediatrics: Child Health and Youth Health, University of Auckland, Auckland, New Zealand

###### **Correspondence:** Robin Cronin

Concern is often raised by maternity providers and families when pregnant and recently bereaved women are approached to participate in stillbirth research. Our aim was to assess factors influencing recruitment in the New Zealand Multicentre Case-Control Stillbirth Study and to gain insight into how women felt about their participation.

Eligible women were contacted through their maternity providers from seven New Zealand health regions between 2011 and 2015. Cases had a recent singleton non-anomalous late stillbirth (≥28 weeks’ gestation). Pregnant controls were randomly selected and matched for region and gestation. Participants were interviewed by a research midwife and given a free-post feedback form asking their views about participation. Feedback was evaluated using thematic analysis.

A total of 169 (254 eligible) cases and 569 (913 eligible) controls were recruited. Non-participants consisted of: 263 (22.5%) women who declined, 106 (9.0%) unable to be contacted, and 60 (5.1%) declined by the maternity provider. Reasons for women declining: no reason provided (166, 63.1%), interview missed/cancelled (42, 15.9%), ‘too busy’ (26, 9.9%), maternal/family anxiety concerns (21, 8.0%), and other factors (8, 3.0%). Of the 60 eligible participants where maternity providers declined on the woman’s behalf: no reason provided (26, 43.3%), concern regarding maternal anxiety (22, 36.7%), social/medical concerns (9, 15.0%) and other factors (3, 5.0%). Written feedback was provided by 111 participants (15.3% of cases and 14.9% of controls) and all described their involvement positively. Two main feedback themes were identified by both the cases and controls: ‘motivation to participate’ (with subthemes of ‘helping others’, ‘finding answers’ and ‘attributing meaning’) and ‘feeling about the experience’ (with subthemes of ‘ease of participation’, ‘talking and sharing’ and ‘personal benefit’).

Identification of recruitment barriers and our reassuring participant feedback may assist researchers and participants in future stillbirth research.

Ethical approval for the study was granted by the Northern “X” Regional Ethics Committee (Reference; NTX/06/05/054). Written informed consent was obtained by all study participants.

### B10 Anatomy of the collateral venous drainage in late pregnancy in different positions

#### Aimee Humphries^1,2^, Peter Stone^1^, Seyed Ali Mirjalili^2^

##### ^1^Department of Obstetrics and Gynaecology, University Of Auckland, Auckland, New Zealand; ^2^Department of Anatomy and Medical Imaging, University Of Auckland, Auckland, New Zealand

###### **Correspondence:** Aimee Humphries

Recent studies have demonstrated an increased risk of late pregnancy stillbirth while sleeping in the supine position. In this position the gravid uterus completely obstructs the inferior vena cava. While this occurs in the majority of women, only a small number experience supine hypotension syndrome. The aim of this study is to investigate the role of collateral venous drainage in late pregnancy in various positions.

After obtaining ethics approval, 10 healthy pregnant women at 35–38 weeks gestation, without supine hypotension syndrome, underwent Magnetic Resonance (MR) scanning in supine and left lateral decubitus positions. MR images (T2 Weighted) were evaluated to measure the calibre and blood flow of the major vessels (inferior vena cava, azygos vein and abdominal aorta) and cardiac output.

Preliminary results have shown that cardiac output remained relatively unchanged in both positions. The blood flow and diameter of the IVC dramatically decreased in the supine position, however, the diameter of the azygos vein was doubled in size.

This MRI study demonstrates for the first time that healthy pregnant women without symptomatic supine hypotension maintain cardiac output when lying flat by collateral venous drainage including the azygos venous system. Variations in the collateral system may affect venous return to the heart, reducing cardiac output and uteroplacental perfusion. This may, in part, explain the effects of maternal position on risk of stillbirth in late pregnancy.

### B11 Maternal sleep physiology in healthy late pregnancy: effect of position on inspiratory air flow

#### Jordan P. R. McIntyre^1,2^, Kevin M. Ellyett^3^, Peter R. Stone^1^, Edwin A. Mitchell^2^, John M. D. Thompson^1,2^, on behalf of the Maternal Sleep in Pregnancy Research Group

##### ^1^Department of Obstetrics and Gynaecology, University of Auckland, Auckland, New Zealand; ^2^Department of Paediatrics: Child and Youth Health, University of Auckland, Auckland, New Zealand; ^3^Respiratory Measurement Laboratory, Auckland District Health Board, Auckland, New Zealand

###### **Correspondence:** Jordan P. R. McIntyre

The Auckland Stillbirth Study (TASS) identified the importance of sleep position in late stillbirth, but relied on mothers’ self-reports, lacking objective physiological explanations for the increased risk. Non-clinically conventional measures of sleep physiology may be required to detect subtle changes associated with maternal position change. Descriptions of maternal sleep behaviour in late pregnancy, such as duration of each sleep position and frequency of position change, are also important in the context of TASS.

30 healthy women with a singleton pregnancy (35-38 weeks) underwent an overnight respiratory sleep study with infrared video. Sleep position was synchronised with the flattening index, an estimate of inspiratory flow limitation. The physiological effects of position were assessed by repeated measures in continuous five minute epochs. Conventional measures of obstructive sleep apnoea (OSA) severity, the apnoea-hypopnoea index (AHI) and oxygen desaturation index (ODI), were also assessed by sleep position. Data are presented as median (IQR).

The AHI and ODI were clinically insignificant in all positions. The supine position demonstrated a substantially greater proportion of flow-limited breaths (24%, 5-55%) than left-lateral (6%, 0-29%) and right-lateral (5%, 0-27%). 20/30 women initiated sleep in the left-lateral position, compared with 8/30 in right-lateral and 2/30 in supine. The women spent a significantly greater overall proportion of the night left-lateral (49%, 37-60%) than supine (19%, 1-29%), and the average duration of each left-lateral before changing position, 35 (26-48) minutes, was significantly longer than supine, 15 (4–32) minutes.

These healthy women in late pregnancy had no clinically-defined OSA in any position, but instead demonstrated marked inspiratory flow limitation when sleeping supine. This flow limitation may contribute to the observed reduction in the time spent supine by increasing arousals from sleep, and thus may have a protective function.

The Regional Human Ethics Committee approved the study protocol (NTX/12/06/048), and all participants provided written informed consent.

### B12 Going to sleep supine and reduced sleep duration are risk factors for late stillbirth: findings from the MiNESS Case-Control Study

#### Alexander E. P. Heazell^1,2^, Minglan Li^3^, John M. D. Thompson^3,4^, Jayne Budd^1,2^, Robin Cronin^3^, Edwin Mitchell^4^, Tomasina Stacey^5^, Devender Roberts^6^, Bill Martin^7^, Lesley M. E. McCowan^3^

##### ^1^Faculty of Biology, Medicine and Health, University Of Manchester, Manchester, UK; ^2^St. Mary’s Hospital, Central Manchester University Hospitals NHS Foundation Trust, Manchester Academic Health Science Centre, Manchester, UK; ^3^Department of Obstetrics and Gynaecology, University of Auckland, Auckland, New Zealand; ^4^Department of Paediatrics: Child Health and Youth Health, University of Auckland, Auckland, New Zealand; ^5^School of Healthcare, University of Leeds, Leeds, UK; ^6^Birmingham Women’s Hospital NHS Foundation Trust, Birmingham, UK; ^7^Liverpool Women’s Hospital NHS Foundation Trust, Liverpool, UK

###### **Correspondence:** Alexander E. P. Heazell

The significant variation in stillbirth rates between and within High Income Countries suggests that more could be done to reduce stillbirth rates. One approach is to identify modifiable risk factors. Two case-control studies from New Zealand and Australia have described an association between going to sleep position and the risk of late stillbirth. In the Midlands and North of England Stillbirth Study (MiNESS) we investigated maternal sleep practices and their association with late (> = 28 weeks’ gestation) stillbirth.

MiNESS was a multi-centre case control study conducted in 40 maternity units in England. In total 291 cases (women with a non-anomalous singleton late stillbirth) and 733 controls (women with ongoing pregnancies) participated. Extensive data were collected using a researcher-administered questionnaire which included questions on sleep practices before pregnancy, and in the four weeks prior to and on the night before the interview/stillbirth.

In multivariable analysis supine going to sleep position the night before stillbirth was thought to have occurred had a greater than 2-fold increased risk of late stillbirth (adjusted Odds Ratio (aOR) 2.31, 95%CI 1.04-5.11) compared to the left side. In addition, sleep duration less than 5.5 hours (aOR 1.83, 95%CI 1.24-2.68), getting up to the toilet once or less (aOR 2.81, 95%CI 1.85-4.26) and a daytime nap every day (aOR 2.22, 95%CI 1.26-3.94) were also associated with late stillbirth. No interaction was detected between the effect of supine going to sleep position and a small for gestational age infant, maternal body mass index or gestation. The population attributable risk for supine going to sleep position was 3.7% (95% CI 0.5-9.2).

This UK study confirms findings from New Zealand and Australian studies that going to sleep position is associated with late stillbirth. We should now consider the best way to change practice via a public health campaign.

Ethical approval for the study was granted by Greater Manchester Central Research Ethics Committee (Reference 13/NW/0874). Written informed consent was obtained by all study participants.

### B13 Intrapartum death and doctors; a qualitative exploration

#### Karen McNamara^1^, Sarah Meaney^1,2^, Keelin O’Donoghue^1,3^

##### ^1^Pregnancy Loss Research Group, Department Of Obstetrics and Gynaecology, University College Cork, Cork, Ireland; ^2^National Perinatal Epidemiology Centre, Department Of Obstetrics and Gynaecology, University College Cork, Cork, Ireland; ^3^The Irish Centre for Fetal and Neonatal Translational Research (INFANT), University College Cork, Cork, Ireland

###### **Correspondence:** Karen McNamara

The death of an infant at any stage in a pregnancy is profoundly traumatic both for the parents and the healthcare professionals involved. Most of the research pertaining to healthcare professionals in this area has focused on the effects of antenatal stillbirth or perinatal death, without investigating the specific impact of unexpected IPD. Our study aims to provide an in-depth qualitative exploration of the attitudes and responses that Irish Obstetricians have following direct involvement with an intrapartum fetal death.

Following ethical approval this qualitative study was conducted in a tertiary university maternity teaching hospital in the Republic of Ireland. Ten obstetricians composed of five consultants and five obstetricians in training were purposively recruited. Semi-structured interviews were conducted in a time and location that suited the participant. The data were analysed using interpretative phenomenology as it explores and understand how individuals make sense of major life experiences.

Direct involvement with an intrapartum death had a profound and negative impact on obstetricians. Devastation, shock, sadness, fear and guilt were some of the emotions experienced by doctors in the aftermath of an IPD. Analysis of the data revealed two superordinate themes; the doctor as a person, and supporting each other. The doctor as person was characterised by two subordinate themes; emotional impact and frustration. Supporting each other was also characterised by two subordinate themes; the good and the bad and what might work.

The impact of intrapartum death on Obstetric doctors is profound and long lasting and doctors are the second victims of these events. This needs greater acknowledgement and acceptance. The development of timely and effective emotional support interventions for all obstetricians is of crucial importance.

Ethical approval for the study was granted by Clinical Research Ethics Committee of the Cork Teaching Hospitals (Reference No: ECM 4(III) 07/07/15 and ECM (III) 08/12/2015).

Written informed consent was obtained by all study participants.

### B14 Confidence and training needs of health professionals providing support to parents who have experienced a loss from a multiple pregnancy

#### Judith Rankin^1^, Louise Hayes^1^, Nicholas Embleton^2^

##### ^1^Newcastle University, Newcastle upon Tyne, UK; ^2^Newcastle upon Tyne Hospitals NHS Foundation Trust, Newcastle upon Tyne, UK

###### **Correspondence:** Judith Rankin

It is well documented that sensitive emotional care from health professionals has a significant impact upon life-long memories formed by parents at the time of the loss of their baby. Less is known about how confident health professionals feel providing this emotional care and if sufficient training is available to support them. This study aimed to ascertain self-rated confidence and training needs of health professionals providing support to parents who have experienced a loss from a multiple pregnancy.

An online survey, consisting of open and closed questions, was sent by email via professional organisations and clinical networks to fetal medicine and neonatal health professionals between March and June 2016. Responses were anonymous.

293 health professionals responded including 130 (44.4%) midwives/obstetricians/fetal medicine specialists and 156 (53.2%) neonatal nurses/doctors. 232 (79.2%) respondents were female and 171 (66.3%) had worked in their current role for more than 15 years. Although confidence in providing practical support to parents who had experienced a loss from a multiple pregnancy was high (156, 58.9%), almost a third of respondents (80, 30.2%) reported having no or some confidence to provide emotional support. Respondents with less time in their current role reported lower confidence in providing emotional support. 131 respondents (47.3%) reported receiving training around loss from a multiple pregnancy, 91 (32.9%) had used national guidelines to inform their practice and 103 (37.2%) knew of local guidelines. 91 respondents (33%) felt that the current training/guidelines were inadequate and 263 (95.3%) felt that more training/guidelines would be helpful.

Self-rated confidence in providing emotional support to parents following a loss from a multiple pregnancy was low. Less than half of respondents had received training on this important aspect of clinical care and the majority of respondents felt more training and further guidelines were needed.

### B15 Caring for the caregiver: using retreats to care for perinatal loss health professionals

#### Lindsey Wimmer, Sarah Rodriguez

##### Star Legacy Foundation, Eden Prairie, MN, USA

###### **Correspondence:** Lindsey Wimmer

Health professionals routinely report the impact of work-related stress on job satisfaction, personal health, and relationships. Health care leaders experience high levels of burn-out and turn-over as a result. Obstetrics is expected to be a positive place to work where poor outcomes are rare, which increases the burden on health professionals in this area when tragedies occur. Perinatal loss has been cited as a primary cause of obstetrics professionals leaving the specialty. Health professionals have also reported feeling inadequately trained to care for families experiencing perinatal death. This lack of knowledge and confidence can magnify the stress on these individuals.

Health professional retreats were designed to support the self-care, educational, and team-building needs of these caregivers. Each retreat is conducted off-site and unique to the attendees. Examples of education sessions include Bereavement Photography, Emerging Evidence in Perinatal Loss, and Understanding Perinatal Autopsy and Evaluation. Self-care modules may include yoga, Reiki, massage, aromatherapy, and more. Parent panels are included in every retreat as is time for brainstorming and team-building activities.

The health professional retreats have been extremely well-received. Event evaluations indicate high levels of rejuvenation, team-building, gratitude, and knowledge. The parent panels are consistently the most popular modules offered. In follow-up surveys, attendees report increased comfort with their role during perinatal loss, decreased personal and work-related stress, increased confidence in their knowledge, and a desire to attend additional offerings.

Health professional retreats can be an effective strategy to decrease work-related stress, burn-out, and turn-over in obstetrics. In addition, they serve to improve care provided to families experiencing perinatal death.

### B16 International variation in the classification of stillbirths and neonatal deaths at 22 to 26 weeks gestational age

#### Lucy K. Smith^1^, Naho Morisaki^2^, Nils-Halvdan Morken^3^, Mika Gissler^4^, Paromita Deb-Rinker^5^, Jocelyn Rouleau^5^, Stellan Håkansson^6^, Michael R. Kramer^7^, Michael S. Kramer^8^

##### ^1^University of Leicester, Leicester, UK; ^2^National Center for Child Health and Development, Tokyo, Japan; ^3^University of Bergen, Bergen, Norway; ^4^National Institute for Health and Welfare, Helsinki, Finland; ^5^Public Health Agency of Canada, Ottawa, Canada; ^6^Umeå University, Umeå, Sweden; ^7^Emory University, Atlanta, GA, USA; ^8^McGill University, Montreal, Canada

###### **Correspondence:** Lucy K. Smith

High-income countries differ substantially in reported survival rates of infants born near the limit of viability. We hypothesized that adherence to “official” criteria of signs of life at birth to classify deaths as stillbirth vs neonatal death, as well as classification of stillbirths as ante- vs intra-partum, may explain much of this variation.

We calculated the number of births by time of death for each completed gestational week using national data on births at 22-26 weeks from the UK (2014 n = 3,264), Japan (2014, n = 3,110), US (2012, n = 24,929), Canada (2009-2014, n = 7,491), Finland (2010-2015, n = 854), and Norway (2010-2014, n = 933) and at 22-25 weeks for Sweden (2011-2014, n = 1,034). We compared the proportion of births classified as antepartum stillbirths, intrapartum stillbirths and live births.

At 22 weeks, large differences were observed among countries in the percent of births classified as ante-partum stillbirth (20%-30%), intra-partum stillbirth (3%-10%) or live birth (48%-65%). For live births, wide variation was observed in the percentage infants dying before 1 hour (11%-42%), suggesting differences in perceptions of viability and in resuscitation practices. All of these differences narrowed with increasing gestational age and nearly disappeared by 26 weeks.

Our findings show wide international differences in the classification of births and deaths for fetuses and infants born near the limit of viability. This makes international comparisons of stillbirth rates and neonatal death rates problematic at very early gestations.

This study is based on publicly available, de-identified, aggregated datasets exempt from ethics review.

### B17 Perinatal 0utcomes using the Robson Ten Group Classification System (RTGCS)

#### Fionan Donohoe, Meenakshi Ramphul, Mark O’Connor, Martina Murphy, Michael Robson

##### National Maternity Hospital, Holles Street, Dublin, Ireland

###### **Correspondence:** Fionan Donohoe

The Robson Ten Group Classification System (RTGCS) is increasingly being used worldwide to compare induction and rates of mode of delivery. It has been less used to simultaneously examine perinatal outcome of childbirth, which is one of the lesser known advantages of the classification. The aim of this study is to suggest a method of using the RTGCS to assess perinatal mortality and morbidity. In particular, the rates of: stillbirths (infant born without any signs of life after 24 weeks gestation and/or weighing more than 500g at birth); intrapartum deaths (death of an infant during labour); early neonatal deaths (death of a liveborn infant within the first seven days of life) and rates of hypoxic-ischemic encephalopathy (infants > 37weeks who have neonatal encephalopathy in the first week of life with evidence of metabolic acidosis in intrapartum fetal umbilical arterial cord or very early neonatal blood samples (pH <7.0, base deficit ≥ 12).

Data were retrospectively collected from contemporaneously written annual reports of a tertiary teaching maternity hospital in Dublin, Ireland.

From the 1st of January 2005 to the 31st of December 2014, there were 88,005 deliveries of infants after 24 weeks gestation and/or who weighed >500g. The perinatal mortality and morbidity rates are described in the table below (Table 2). The rates reported include congenital anomalies. The overall perinatal mortality rate during that time period ranged from 5.6 to 9.8 per 1,000.

As recommended by the World Health Organisation and as increasing numbers of countries worldwide implement the RTGCS to compare induction and rates of mode of delivery, it is important to remember that other perinatal outcomes can, and should, be analysed using the same system. This will allow focussed interventions on prospective groups of women to take place depending on local results.

**Table 2 (abstract B17).** See text for description

	Stillbirth*	Intrapartum deaths*	Early neonatal death*	Hypoxic-ischaemic encephalopathy
	(per 1,000)	(per 1,000)	(per 1,000)	(per 1,000)
Nulliparous singleton cephalic, >37weeks (Robson groups 1,2)	83/35406 (2.3)	0/35406 (0)	19/35406 (0.5)	50/35406 (1.4)
Multiparous singleton cephalic, > 37weeks, excluding previous caesarean section (Robson groups 3,4)	46/35317 (1.3)	0/35317 (0)	17/35317 (0.5)	25/35317 (0.7)
Previous caesarean section, singleton cephalic, >37weeks (Robson group 5)	13/8682 (1.5)	0/8682 (0)	6/8682 (0.7)	5/8682 (0.6)
Multiple pregnancies (Robson group 8)	27/1647 (16.3)	0/1647 (0)	53/1647 (32.2)	5/1647 (1.2)
Malpresentation, deliveries <37 weeks (Robson groups 6,7,9,10)	192/6953 (27.6)	2/6953 (0.3)	126/6953 (18.1)	0/6953 (0)

### B18 Small for gestational age and perinatal mortality at term: an audit in a Dutch national cohort study

#### Martine Eskes^1^, Adja Waelput^2^, Sicco Scherjon^3^, Klasien Bergman^3^, Ameen Abu-Hanna^1^, Anita Ravelli^1^

##### ^1^Academic Medical Center, Amsterdam, The Netherlands; ^2^Erasmus MC, Rotterdam, The Netherlands; ^3^University Medical Center Groningen, Groningen, The Netherlands

###### **Correspondence:** Martine Eskes; Anita Ravelli

An important goal of prenatal care is a timely detection of fetal growth restriction, and prevention of fetal asphyxia or perinatal mortality and morbidity by fetal monitoring and timely birth. Many studies showed the low detection rate of SGA during pregnancy, which varied between 15-32%. We assessed the underlying risk factors for perinatal mortality in term born SGA infants.

We performed a population based nationwide cohort study in the Netherlands of 465,532 term born infants from January 2010 to January 2013. Logistic regression analyses were performed. Also audit results were investigated for detailed care information.

We studied 162 SGA infants who died in the perinatal period. Risk factors were: gestational age between 37.0-37.6 weeks (adjusted Odds Ratio (aOR) 2.60, 95% Confidence Interval (CI) 1.58, 4.28), male gender (aOR 1.39, 95% CI 1.01, 1.90), South Asian ethnicity (aOR 3.63, 95% CI 1.58, 8.35), African (aOR 3.54, 95% CI 1.93, 6.49), and other non-Western ethnicity (aOR 1.92, CI 1.18, 3.10). At 37.0-37.6 weeks gestation audit results showed that 26% of the women smoked, 91% were boys and in all but one case death occurred before birth. In 61% of all deceased SGA infants born at 37.0-37.6 weeks gestation, referral from primary care by independent midwives to the obstetrician took place because of antepartum death before labour.

Gestational age between 37.0-37.6 weeks, male gender, South Asian, African or other non-Western ethnicity and smoking are associated with perinatal mortality in SGA infants. These risk factors concern the complete term population starting at 37.0 weeks or even earlier. Therefore, it is of utmost importance to develop accurate diagnostic tests to screen for SGA before 36 weeks gestation to prevent perinatal.

## Poster presentation

### P1 Bereavement care services within the Maternity unit at Our Lady of Lourdes Hospital, Drogheda

#### Fiona Mulligan, Colette McCann, AnnMarie Connor

##### Our Lady of Lourdes Hospital, Drogheda, Ireland

###### **Correspondence:** Fiona Mulligan

This poster outlines the advancements in Bereavement Services within the Maternity Unit of Our Lady of Lourdes Hospital, Drogheda.

In 2012, in spite of the financial and operational challenges the ‘Butterfly Room’ was developed to provide a quiet comfortable space for bereaved parents and their baby. It allows for the parents to spend time with their baby and close family and friends before their final goodbye. The use of a Cuddle cot donated by Feileacain (again as a result of fundraising from bereaved parents) allows for the baby to remain with the parents for as long as they wish.

The Flower room was later established in order to provide a cool environment for baby prior to Postmortem or burial in accordance to the wishes of the parents. This was followed by the appointment of The Bereavement support midwife in 2013. Practical and emotional advice and support is facilitated by the bereavement team-Bereavement midwife, Pastoral care and Medical social work and staff at ward level, at this very difficult time.

Subsequently in 2015 the ‘Dragon Fly Room’ was opened to provide a quiet private space for parents who receive bad news during pregnancy and also for women and their families who receive poor diagnosis from the Gynaecological ward. This came about as a result of fundraising by two families who had been affected by the loss of a baby through stillbirth.

We continue to develop our bereavement services with the support of our Bereavement Midwife, the Multi-disciplinary Bereavement Team, The Breaking Bad News Training Programme and most recently, The National standards for Bereavement care following Peri-natal death and Pregnancy loss.

Ethical approval was not required as this work was a service evaluation.

### P2 Breaking the conspiracy of silence - narratives about disenfranchised grief and actions to enfranchise it

#### Juha Itkonen

##### University Of Helsinki, Orivesi, Finland

Stillborn children were surrounded by silence for a long time. The issue of having a stillborn has been something awkward; not a proper topic to speak about, not to mention to mourn. Even today the parents tend to be met with silence, intended as a form of kindness, by care givers in hospital and church, friends stopping by at home and coworkers on the job.

The data of the first phase was collected by narrative interviews (N = 24). Interviews were analyzed with computer assisted NCT –method (notice, collect, think). The second phase was an action research. With KÄPY - The Child Death Families and The Federation of Finnish Midwives we started a citizens’ initiative to change the law so that stillborn children could be written in the Finnish Population Information System by their own names. Response to the initiative in internet conversations were also analyzed with NCT –method.

Parents did not feel encounters with professionals supportive, rather formal and distant. When support was received, the helper did not approach the bereaved parent as a professional but as a human being who had courage to come close and be there. The Finnish media were first reluctant to publish articles about our citizens’ initiative and when we get our message trough, a lot of comments were dismissive to the subject. These can be seen in framework of disenfranchised grief. However, our action research process broke the silence in some degree.

The results shows that “a conspiracy of silence” is still an issue in Finnish context, although there were a lot of narratives about supportive encounters too. Seems that the first step to help a parents of a stillborn is recognizing that the they have sustained a real loss, a death of a human being.

The research was carefully made by the guidelines of the Finnish Advisory Board on Research Integrity. Written informed consent was obtained by all 24 study participants.

### P3 Stillbirth Stories: audio oral history archive of personal testimonies - a tool for peer support, professional learning and public engagement

#### Emma Beck^1^, Nicola Gibson^1^, Alexander Heazell^1,2^

##### ^1^Stillbirth Stories, London, UK; ^2^University of Manchester, Manchester, UK

###### **Correspondence:** Emma Beck

‘Stillbirth Stories’ is a Wellcome Trust funded oral history archive documenting the experience of stillbirth from the perspective of parents and professionals. The testimonies gathered on the open-access website will provide a unique peer support resource for those directly affected by the experience of stillbirth and for those who care for them.

The value of access to the material gathered is anticipated to be both immediate and lasting for bereaved parents, families and friends especially parents from “hard to reach” groups or those who may have not accessed formal support before. It is hoped that for interested clinicians and other professionals it will provide the basis of a tool for informal and formal learning.

Inspired by personal experiences, two former documentary producers worked with the perinatal bereavement team at St Mary’s Hospital, Manchester to record parents’ and professionals experiences in 60-90 minute interviews covering the clinical and bereavement care received/provided. The first phase of www.stillbirthstories.org will feature 20+ audio interviews and transcriptions. The audio interviews can also be searched by ‘Theme’: headings include: ‘The birth’; ‘Spending time with your baby/making memories, ‘Deciding about a post-mortem’, ‘Getting pregnant again’.

The stillbirthstories.org site will launch in Autumn 2017. A functioning section can be viewed at: http://nominomi.com/stillbirthstories/. Evaluation has included a survey of maternity staff to address whether the opportunity to listen to clinical and personal experiences of bereaved parents and other clinicians improves clinicians’ confidence in caring for families who have had a stillbirth. The results of this evaluation will be presented.

This project has established a methodology that could be applied in other countries/units to extend the archive. Personal testimony can be used as a peer support resource for bereaved parents, a tool for professional learning and to engage the public and raise awareness

The archive itself did not require ethical approval, but all interviewees gave written consent for their interviews to be used.

### P4 Women’s attitudes, experiences and compliance concerning the use of Mindfetalness-a method for systematic observation of fetal movements in late pregnancy

#### Anna Akselsson^1,2^, Susanne Georgsson^1,2^, Helena Lindgren^1^, Karin Pettersson^1^, Ingela Rådestad^2^

##### ^1^Karolinska Institutet, Stockholm, Sweden; ^2^Sophiahemmet University, Stockholm, Sweden

###### **Correspondence:** Anna Akselsson

Maternal perception of decreased fetal movements and low awareness of fetal movements are associated with a negative birth outcome. Mindfetalness is a method developed for women to facilitate systematic observations of the intensity, character and frequency of fetal movements in late pregnancy. We sought to explore women’s attitudes, experiences and compliance in using Mindfetalness.

We enrolled 104 pregnant women treated at three maternity clinics in Stockholm, Sweden, from February to July of 2016. We educated 104 women in gestational week 28-32 by providing information about fetal movements and how to practice Mindfetalness. Each was instructed to perform the assessment daily for 15 minutes. At each subsequent follow-up, the midwife collected information regarding their perceptions of Mindfetalness, and their compliance. Content analyses, descriptive and analytic statistics were used in the analysis of data.

Of the women, 93 (89%) were positive towards Mindfetalness and compliance was high 78 (75%). Subjective responses could be binned into one of five categories: Decreased worry, relaxing, creating a relationship, more knowledge about the unborn baby and awareness of the unborn baby. Eleven (11%) women had negative perceptions of Mindfetalness, citing time, and the lack of need for a method to observe fetal movements as the most common reasons.

Women in late pregnancy are generally positive about Mindfetalness and their compliance with daily use is high. The technique helped them to be more aware of, and create a relationship with, their unborn baby. Mindfetalness can be a useful tool in antenatal care. However, further study is necessary in order to determine whether the technique is able to reduce the incidence of negative birth outcome.

The women gave consent to receiving the study material. The study was approved by the Regional Ethical Review Board in Stockholm: DNR: 2015/2105-31/1.

### P5 Renegotiating father’s identity following stillbirth: what and who am I?

#### Kerry Jones^1,2^

##### ^1^The Open University/The Talking Shed, Milton Keynes, UK; ^2^The Talking Shed [Counselling services], Credtion, UK

This study examines the experiences of men following stillbirth in particular the challenges they face in claiming their identity as a father of an absent child. Fathers felt diminished when concerns about how they were coping were directed only to the women. Contrary to the notion that father’s experience suggests men suffer less distress, this research shows that men also deal with loss at an emotional level.

This investigation into men’s accounts of loss forms part of a larger study in which 28 men and women participated in interviews and focus groups about their experiences of perinatal death.

By listening to narrative accounts of loss, the passage to parenthood for bereaved men represents a disruption and re-evaluation of who they are, what they knew about the world as they negotiate the incomprehensibility of the death itself. Narratives by bereaved men also reveal how their sense of self and identity is mediated by the social and cultural milieu to which they belong and are largely disenfranchising experiences when friends, family and others, at times, fail to acknowledge the enormity of their loss.

The findings suggest that recognition of the death of baby who is stillborn as well as the impact of the death for father’s is intertwined with personal identity. Men in this study needed to receive recognition as fathers, both at the time of their loss and after. In examining the reproductive and bereavement journey of men, several domains occurred to illuminate the experience of men including; men as support partners; the impact of the death; parenting an absent child [advocate, protector]. The findings from this study will offer insight into the experiences of men that will resonate for others including practitioners who support individuals going through similar experiences.

Ethical approval for the study was granted by the University of Bristol Ethics Committee as part of doctoral research. Written informed consent was obtained by all study participants. No formal recruitment was obtained through the National Health Service or Government Institution and was entirely voluntary.

### P6 The four-leg model of recovery - clues to identify parents of stillborn at high-risk

#### Juha Itkonen

##### University Of Helsinki, Helsinki, Finland

Grief is fundamental part of human life and people grieve in various manners. However, all ways to grieve are not equally beneficial. It is difficult to recognize those mourners at higher risk, because of the multidimensional nature of grief. Recent studies have shown that many factors can complicate the grief of having a stillborn child. This paper aims to discover the significant risk-factors in stillbirth-grief and presents a model to identify them.

The data of this paper was collected by narrative interviews (14 mothers and 10 fathers of stillborn child). Interviews were analyzed with computer assisted NCT –method (notice, collect, think) with help of Atlas.ti –software. The Four-Leg-Model of recovery was created from the data and based contemporary grief theories. The data was analyzed again in the light of this Model.

The first result was the analysis-tool itself. According to The Four-Leg-Model the recovery is like a stool with four legs. These legs are nature of loss, personal resources, social support and other concurrent stressors. If one of these legs are broken, a mourner will most likely recover. If two, a mourner is at a high-risk of falling. If three, a mourner will almost certainly fall. If all four, immediately actions are needed. Results also revealed that grief after having a stillborn can test the firmness of all three of the loss-related legs. After loss, social support is only leg that can be affected directly and only through social support can help be offered in dealing with the concurrent stressor and personal resource legs.

All forms of social support – emotional, tangible, informational and spiritual – play an essential role in helping mourners. The Four-Leg-Model gives professionals a useful tool to identify mourners at high-risk – not only after a stillbirth, but in any loss situation.

The research was carefully made by the guidelines of the Finnish Advisory Board on Research Integrity. Written informed consent was obtained by all 24 study participants.

### P7 Perinatal palliative care after a stillbirth - midwives experiences of using Cubitus baby

#### Ingela Rådestad, Karin Henley Listermar

##### Sophiahemmet University, Stockholm, Sweden

###### **Correspondence:** Ingela Rådestad

In Sweden, around 450 babies are stillborn every year. Usually, the parents stay at the hospital a couple of days after the birth and they can have the baby in their room. Due to the importance to keep a dead body cold it has, until recently, been a routine to separate the baby from the parents and place the baby in a refrigerator during the night. With the goal to improve the dignity for the baby and the family a tool was developed. Cubitus baby, a special cot with cooling blocks, was implemented at all 48 delivery wards in Sweden during 2013-2014.

The aim of the study was to investigate the midwives experiences of using Cubitus baby. In total 155 midwives answered a questionnaire. One open question was analyzed with content analyses.

Five categories were formed concerning the midwives experiences; a gracious feeling, a sense of relief in their work, caring with coldness, time to say goodbye and a good feeling for the parents.

Cubitus Baby is an essential tool for the midwife when they provide perinatal palliative care. The midwife can give time to say farewell without feeling stressed that they must separate the baby from the parents.

### P8 A review of the stillbirth registration process in the UK: support and care offered to parents

#### Bethany Jakubowski, Laura Oakley, Diane Duclos

##### London School Of Hygiene And Tropical Medicine, London, England

###### **Correspondence:** Bethany Jakubowski

To review the stillbirth registration process in the UK and identify gaps in the care and support offered to parents.

**Fig. 2 (abstract P8).**
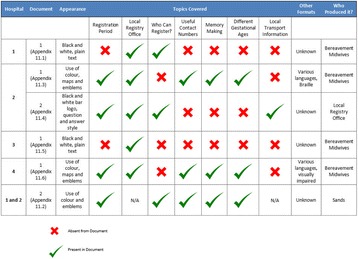
Table comparing the documents included in the document analysis

This mixed-method study included a document analysis (see Fig. 2) and five face-to-face, semi structured interviews. The documents gathered were samples of the written information provided to parents about the stillbirth registration process, taken from a purposive sample of four hospitals in the London area. The first round of interviews conducted with five stillbirth charity workers were identified through purposive and snowball sampling. One follow-up phone interview was also conducted.

The primary themes identified using a thematic analysis were; the lack of consistent care, the importance of bereavement midwives, a desire for change to the registration process, and the need to normalise stillbirth through support networks. Most charity workers identified that registering a stillbirth was a traumatic and difficult experience, and made suggestions to improve the process, such as changing the locality of the registration and lowering the gestational age specified in the registration law. All charity workers interviewed viewed two samples from the document analysis to be unsympathetic to bereaved parents. The analysis of the documents demonstrated there are inconsistencies in written information provided to bereaved parents.

This study demonstrates a strong desire among stillbirth charity workers to change the registration process, and for a consistent level of care to be provided to bereaved parents. Findings from both the interviews and the document analysis demonstrate parents currently receive an inconsistent level of care. Participants suggested the level of care and support provided by bereavement midwives can vary, indicating the need for standardisation at a national level. Efforts should be made to include perspectives of bereaved parents to pinpoint gaps and weaknesses in current practices of care and support provided during the stillbirth registration process.

### P9 Placental findings and perinatal deaths - a national review using consensus terminology

#### Yvonne McCartney^1^, Edel Manning^2^, Irene O’Farrell^2^, Eoghan Mooney^1,2^

##### ^1^National Maternity Hospital, Holles Street, Dublin, Ireland; ^2^National Perinatal Epidemiology Centre, Cork, Ireland

###### **Correspondence:** Yvonne McCartney

The National Perinatal Epidemiology Centre (NPEC) collects anonymised perinatal mortality data from all 19 Irish maternity units. Consensus terminology has been published to assist in reporting of placental pathology, and is now used in Ireland on a national basis.

Placental pathology contributes to or causes stillbirth in 11 to 65% of cases in various classifications. Maternal vascular malperfusion (MVM) and fetal vascular malperfusion (FVM) play a major role. We assessed perinatal deaths where placental disease is the main cause of death, focusing on MVM and FVM.

For the years 2014 and 2015, all cases of early and late neonatal deaths (regardless of gestation and birthweight at delivery) and stillbirths (≥24 weeks gestation or birthweight ≥500g) were included when placental disease was the main cause of death or an associated factor (n = 571).

MVM and FVM were reported in 73.7% and 77.9% of stillbirths and in 26.3% and 22% of neonatal deaths, respectively. There were 28 (5%) cases with both MVM and FVM. Both MVM and FVM were more common in males (57.5% and 55.2%). Co-existing pathologies included intra-uterine growth restriction (3.9% of MVM) and cord pathology (9.5% of FVM).

This is the first application of placental consensus terminology to national data of which we are aware. The frequency of placental pathology is consistent with previous studies. Our results highlight the major contribution to perinatal loss of MVM and FVM. The co-existence of MVM and FVM is an under-recognised phenomenon and is seen in 5% of cases.

Ethical approval for the NPEC national clinical audits of obstetric outcomes was provided by the Clinical Research Ethics Committee of the Cork Teaching Hospitals (Reference: ECM 4(g) 05/08/08

### P10 Mixed maturation patterns - when acceleration meets delay

#### Yvonne McCartney, Marie Culliton, Paul Downey, Eoghan Mooney

##### National Maternity Hospital, Holles Street, Dublin, Ireland

###### **Correspondence:** Yvonne McCartney

Placental maturation that is appropriate for gestation is important in ensuring optimal fetal development. An accelerated maturation pattern reflects maternal vascular malperfusion (MVM), the leading cause of perinatal loss, seen in approximately one third of cases. Delayed villous maturation (DVM) has also been associated with perinatal loss and is reported in 5 – 7% of placentas. A combination of both accelerated and delayed maturation patterns could be expected in a number of cases.

The aim of this study was to compare a cohort of placentas diagnosed as DVM or as a mixed pattern (features of both accelerated and delayed maturation).

Cases with a diagnosis of DVM (n = 116) and those with a diagnosis of a mixed pattern (n = 116) were selected from the pathology database between 2013 and 2016. Cases were reported by a single experienced placental pathologist. Gross details were recorded, including cord insertion, cord coiling and the presence or absence of placental disruption. Clinical details obtained included maternal age and parity, mode of delivery, gestation at delivery, birth weight and infant gender.

Groups were comparable in terms of gestational age, gender and mode of delivery. Disruption of the maternal surface was similar in both groups. Cases with a mixed maturation pattern showed a statistically significantly smaller placenta size (fetoplacental weight ratio p value 0.0113) and lower birth weight (p value 0.0263).

A mixed pattern of placental maturation correlates with features that suggest MVM.

This study uses anonymised laboratory data, ethical approval was neither sought nor required.

### P11 Women’s mental health following a miscarriage: the influence of personal and contextual variables

#### Francine deMontigny^1,2^, Chantal Verdon^2,3^, Sophie Meunier^4^, Isabel Coté^1,2^, Christine Gervais^2,3^

##### ^1^Université Du Québec En Outaouais, Gatineau, Canada; ^2^Center of Research and Studies on Family Intervention, Gatineau, Canada; ^3^Université du Québec en Outaouais, St-Jérôme, Canada; ^4^Université du Québec à Montréal, Montréal, Canada

###### **Correspondence:** Francine deMontigny

In Western societies, it has been estimated that between 20% and 25% of pregnancies end in spontaneous abortion (miscarriage). Despite this high prevalence, bereavement associated with miscarriage has received much less attention from the scientific and professional communities than that associated with stillbirth.

This cross-sectional study aimed to examine personal and contextual variables associated with women’s mental health following a miscarriage. A total of 231 women who had miscarried in the past 4 years answered a self-report questionnaire assessing their mental health (depression, anxiety, perinatal grief) and collecting personal as well as contextual characteristics.

One-way analyses of variance indicated that women who had miscarried within the past 6 months had higher scores for depressive symptoms than did women who had miscarried between 7 and 12 months ago, while anxiety level and perinatal grief did not vary according to time since miscarriage. Moreover, low socioeconomic status, immigrant status, and childlessness were associated with worse mental health following a miscarriage. In contrast, regression analysis indicated that quality of the conjugal relationship and satisfaction with health care were positively associated with women’s mental health.

Results suggest that researchers and health professionals working with women experiencing miscarriages should pay particular attention to women living in vulnerable situations (e.g. low socioeconomic status, immigrant status, childlessness). Quality of the conjugal relationship and satisfaction with health care appear to be important protective factors and could be the target of interventions aimed at reducing the deleterious effect of miscarriage on women’s mental health.

### P12 Rates, causes and risk factors of stillbirth in a large cohort of pregnant women from peri-urban areas of Karachi, Pakistan

#### Muhammad Imran Nisar, Muhammad Ilyas Ilyas, Urooj Fatima, Komal Naeem, Yasir Shafiq, Fyezah Jehan, Anita Zaidi

##### Aga Khan University, Karachi, Pakistan

###### **Correspondence:** Muhammad Imran Nisar

An estimated 2.6 million babies are stillborn every year with the highest Still Birth Rate (RBR) in Pakistan of 43.1 stillbirths/1000 births. We describe here burden, risk factors and causes of stillbirths in a cohort of pregnant women from low income community setting in Karachi.

From Jan 2012 – Dec 2013, Community Health Workers (CHWs) identified newly pregnant women through 3 monthly household visits. They were followed up till end of their pregnancy. In case of a stillbirth, a detailed verbal autopsy (VA) form was filled. Forms were reviewed by 2 independent Physicians and 3 physicians in case of disagreement to assign a cause of stillbirth. Consensus between at least two physicians was required for a definitive cause.

There were 17,321 births in total, including 16,786 live births and 535 stillbirths giving SBR of 30.8/1000 births. Previous history of stillbirth, mother’s age >30 years, nulliparity, deliveries conducted by skilled birth attendant or in hospitals, squatting position during delivery and antibiotic use at time of delivery were found to be associated with high risk of stillbirth. During pregnancy, iron supplementation, tetanus toxoid injection and physical work were found to decrease risk of stillbirth. Complications during pregnancy (vaginal bleeding, foul smelling discharge or convulsions) and at time of delivery (water breaking before pain began, premature rupture of membrane, excessive vaginal bleeding, foul smelling discharge, convulsions, abnormal fetal presentation, prolonged labor or fever within a week of delivery) were associated with high risk of stillbirths. VA coding identified pregnancy induced hypertension, antepartum hemorrhage and obstructed labor as most important causes of stillbirths.

We report a lower than reported SBR for Pakistan and identify a spectrum of risk factors and protective factors for still births. This reemphasizes need for good quality antenatal and perinatal care to decrease burden of stillbirths.

### P13 Structured daily observation of fetal movements is associated with fewer children referred to neonatal care

#### Anna Akselsson^1,2^, Anders Linde^1,2^, Susanne Georgsson^1,2^, Helena Lindgren^1^, Karin Pettersson^1^, Ingela Rådestad^2^

##### ^1^Karolinska Institutet, Stockholm, Sweden; ^2^Sophiahemmet University, Stockholm, Sweden

###### **Correspondence:** Anna Akselsson

Low awareness of fetal movements is associated with negative pregnancy outcome and the knowledge about pregnant women’s use of methods when they observe fetal movements is limited. We aimed to investigate how women, seeking care due to decreased fetal movements, pay attention to fetal movements in the third trimester. A specific aim was to identify if the degree of awareness had any effects on pregnancy outcome.

In this prospective study a questionnaire was distributed to 2683 pregnant women who contacted health care due to decreased fetal movements between January 1st and December 31st 2014, at all seven maternity clinics in Stockholm. The questionnaire was presented to the women after a physical examination where no signs of a compromised fetus were identified. The women were followed until birth and outcome data were collected from an obstetric record registry.

Women who counted the fetal movements daily had a lower risk of their child being referred to neonatal care (RR 0.25, 95% CI 0.03-0.94) and had a caesarian section before onset of labor more often (RR 1.6, 95% CI 1.2-2.2) compared with women that observed fetal movements in an unstructured way daily. Compared with women born in Sweden, a counting method was used more commonly by women born in Asia (RR 4.0, 95% CI 3.1-5.2), Africa (RR 4.0, 95% CI 2.7-5.8), Europe (RR 2.3, 95% CI 1.6-3.3), South America (RR 3.1, 95% CI 1.7-5.5) and North America (RR 4.2, 95% CI 1.8-10.0). A counting method was also used more often by women with lower education (RR 2.7, 95% CI 1.7-4.2).

Women who observed fetal movements daily by counting the movements had a lower risk of having their child referred to a neonatal clinic than those using an unstructured method.

The women gave consent to participate and permission to access supporting data when receiving information about the study. The data will not be made available in order to protect the participant’s identity. The study was approved by the Regional Ethical Review Board in Stockholm: DNR: 2013/1077-31/3.

### P14 Assisting health professionals in supporting fathers after stillbirth

#### Francine deMontigny^1,2^, Chantal Verdon^2,3^, Christine Gervais^2,3^

##### ^1^Université Du Québec En Outaouais, Gatineau, Canada; ^2^Center of Research and Study in Family Intervention, Gatineau, Canada; ^3^Université du Québec en Outaouais, St-Jérôme, Canada

###### **Correspondence:** Francine deMontigny

In spite of health professionals’ good will, fathers’ grief after a stillbirth remains invisible and is barely taken care of by the health services in Canada. Different bereavement and socialisation theories have attempted to explain men’s bereavement and their associated reactions, in order to help bridge the gap between bereaved fathers’ needs and the help and support offered. Meanwhile, research with health professionals have underlined their lack of education in regards to the triple challenges of composing with masculinity, fatherhood and bereavement. Our research team has been conducting, over the past 15 years, a program of research, which, combined with our extensive clinical experience, has enabled us to develop, implement, and evaluate a set of tools (online video, books and workshops) to assist health professionals in supporting fathers after stillbirth. The Father Bereavement Project (Movember grant 2014-2017) was implemented in two regions of Quebec, Canada in 2015.

This presentation will describe the one day workshop, its implementation and the effects on health professionals’ sense of efficacy towards fathers.

The implementation phase 1 reached 75 health professionals. The evaluation phase was carried out through a pre-post self-report questionnaire, which was analyzed with SPSS 20.

The workshop’s objectives, themes and strategies will be shared. Analysis revealed that participating health professionals reported higher self-efficacy after the workshop.

Health professionals sense of self-efficacy working with bereaved fathers can be strengthened through innovative workshops that integrate reflexive approaches and grief theories. The discussion will explore implications for bereavement education, research and clinical practices to enhance support for fathers after stillbirth.

### P15 Parents’ experiences of medical care during second trimester miscarriage

#### Sarah Cullen^1^, Barbara Coughlan^2^, Brenda Casey^1^, Sheila Power^3^, Anne McMahon^2^, Mary Brosnan^1^

##### ^1^National Maternity Hospital, Dublin, Ireland; ^2^University College Dublin, Dublin, Ireland; ^3^Psychoanalytic psychotherapist in Private Practice, Dublin, Ireland

###### **Correspondence:** Sarah Cullen

Second trimester miscarriage is defined as pregnancy loss between 12 and 24 weeks gestation A recent study conducted in a large Dublin maternity hospital reported a rate of second trimester miscarriage as 0.8%. The hospital admission has been identified as a critical part of mothers’ experiences of miscarriage and can greatly influence the mother’s recovery. The overall aim of the study was to explore parents’ experiences of hospital care during a second trimester miscarriage. This presentation will report on mothers’ and fathers’ views on the medical care received in the hospital from the time of diagnosis of the second trimester miscarriage through to follow-up care.

A focused ethnographic design was used and a series of semi-structured interviews were completed with 14 bereaved parents [9 mothers and 5 fathers]. Thematic network data analysis was used to analyse the data. Ethical approval was granted by both the Hospital and University Ethics Committees.

Under the first Global Theme of Clinical Care Needs both mothers and fathers highlighted the need for effective medical care in relation to medical treatment, pain relief and length of hospital stay. Parents highlighted the importance of adequate pain relief throughout the labour and birth. Of particular importance to some parents was the going home to prepare following the diagnosis of intra uterine death and the follow up care which they received.

Second trimester miscarriage is a significant life event for parents. The parents’ encounter of medical care received has the potential to impact positively on their experience. Recommendations for clinical practice centre on individualised pain assessment, phlebotomy procedures, follow-up investigations and appointments.

### P16 Perinatal hospice care; a different journey

#### Sarah Cullen, Heather Hughes, Barbara Cathcart, Brenda Casey

##### National Maternity Hospital, Holles street, Dublin, Ireland

###### **Correspondence:** Sarah Cullen

Pregnancy and child birth is generally a joyful experience for parents. However, when a diagnosis of life limiting condition is made during pregnancy, parents embark on a different journey. Parents need supportive, compassionate, individualised care from experienced health professionals. With this in mind a care pathway for families whose baby has been diagnosed with a life limiting condition has been developed. This care pathway adopts a multi-disciplinary specialist approach to provide individualised care to families.

The aims of care for families with diagnosis a life limiting condition is to provide compassionate supportive care from a team of experienced health professionals. At diagnosis parents are linked with a Prenatal diagnosis midwife, a Fetal medicine consultant and the Bereavement midwives. Care is provided using a multi-disciplinary team approach. The team consist of two Prenatal Diagnosis midwives, two Bereavement midwives and Consultant Obstetricians specialising in Fetal Medicine. The team is supported by other disciplines including Paediatricians, Social workers and Chaplains.

Parents are provided with individualised information regarding their baby’s condition and prognosis. Families are supported in memory making both during the pregnancy and the neonatal period. The Bereavement midwives are available to families for practical and emotional support. Parents are offered a follow up appointment with their Consultant and the Bereavement midwives following the delivery to allow for further discussions and provide on-going support.

Parents whose baby has been given a diagnosis of a life limiting condition embark on a very different journey to other parents. Families are empowered through this pregnancy journey within a supportive sensitive environment. Audit of this service is undertaken regularly using feedback from the parents and the team members. Parents are provided with individualised multi-disciplinary care from a team of experienced professionals and are guided through the journey from diagnosis to delivery and beyond.

### P17 Association of maternal sleep patterns with risk of late stillbirth in high income countries: a literature review

#### Ayesha Mir^1,3,4^, Matthew Hewitt^1,2,4^

##### ^1^Bon Secours Hospital, College road Cork, Cork, Ireland; ^2^Cork University Hospital, Cork, Ireland; ^3^Royal College of Physicians of Ireland, Dublin, Ireland; ^4^Royal College of Obstetrician and Gynaecologists, London, UK

###### **Correspondence:** Ayesha Mir

Still birth is associated with profound adverse psychosocial outcome for families and care providers. Global burden of stillbirth is very high with an estimated 2.6 million still births at 28 weeks or more occurring every year with the rate of >1/200 births in high income areas. Studies have examined risk factors for still birth but they have been unable to explore a broad range of potential risk factors in particular those relating to maternal life style and personal habits e.g obesity is highly linked to sleep related disorders.Around 1/3 of a person’s life is spent asleep but there has been little research on potential impact of sleep practices on developing foetus.

We searched the English literature from January 2006 till February 2017 for all articles related to the title of review. Data sources were Embase, Medline, Cochrane Library, PubMed, BMJ and WHO and UNICEF publications.

Studies have concluded that foetal heart rate variability is affected by maternal position.These effects are most likely foetal adaptations to positions which may produce a mild hypoxic foetal stress. Literature shows no association between risk of still birth with snoring either before or during pregnancy. A high twofold rise in still birth risk has been shown with non-left sided sleeping position on the last night of pregnancy with sleeping on back shown the greatest risk.Moreover a significant relationship has been found between sleeping regularly in daytime and late still birth risk. Getting up for toilet infrequently (once or less) has also been found significantly associated with late still birth risk.

Given the results observed in these studies, it may be required from all health care providers to emphasize optimal sleeping practices especially in late pregnancy. Further Studies with prospectively collected data are required to confirm or refute these findings.

### P18 Genetic disease in patients with fetal death

#### Monica Aguinaga^1^, Samuel Carmona^1^, Maria Cervantes^1^, Irma Monroy^1^, Rosalba Sevilla^1^, Yolotzin Valdespino^2^, Elsa Moreno^3^, Rodrigo Zamora^3^, Arturo Cardona^4^

##### ^1^Genetics and Genomics Department. Instituto Nacional De Perinatología, Mexico City, Mexico; ^2^Pathology Department. Instituto Nacional de Perinatologia, Mexico City, Mexico; ^3^Medical Direction. Instituto Nacional de Perinatologia,, Mexico; ^4^General Direction. Instituto Nacional de Perinatologia,, Mexico

###### **Correspondence:** Monica Aguinaga

Fetal death defined as the death occurring after 22 weeks of pregnancy can be caused by maternal, fetal and placenta factors. Congenital defects are a common cause of fetal deaths. They can be isolated or part of a genetic disease which can be chromosomal, monogenic or polygenic. In 2012 it was reported that 7.3% of stillbirths in Mexico were attributed to congenital disorders. The aim of this study was to evaluate all patients with fetal death during two years in the National Institute of Perinatology, Mexico City. We describe the congenital defects found in the patients.

Patients with fetal death born during 2015 and 2016 were evaluated by a medical geneticist at birth. A complete prenatal and postnatal clinical evaluation, radiography and chromosomal analysis were performed. Consent of parents was taken to perform karyotype and/or MLPA (multiplex ligation probe amplification) and necropsy. Chromosomal and DNA analysis was performed in a sample of umbilical cord.

During the study period a total of 473 stillbirths were born. Congenital defects were observed in 142 (30%) patients which are shown in Table 3. The most common cause was chromosomal anomalies (33%), followed by hydrops fetalis (10.5%) and renal anomalies (9.8%). Karyotype was obtained in 60% of samples, we validated the MLPA technique in umbilical cord and were able to obtain a result in 97% of cases.

Congenital defects cause 30% of fetal deaths in our Institute. The most common cause is a chromosomal anomaly which has been successfully diagnosed by the MLPA technique. A microarray will help to define if babies with multiple congenital defects present pathogenic copy number variants. We need to perform more studies in patients with hydrops fetalis and renal diseases in order to provide a better reproductive risk for parents.

Ethical approval for the study was granted by the National Institute of Perinatology, Mexico City Research Ethics Committee. Written informed consent was obtained by all study participants

**Table 3 (abstract P18).** Causes of congenital defects in stillbirth

GENETIC DISEASE	Number of patients	GENETIC DISEASE	Number of patients
Chromosomal	47	Cardiac	4
Caudal anomalies	11	Monogenic	10
Multiple anomalies	8	Diaphragmatic hernia	5
IUGR	4	Hydrops fetalis	15
Bone dysplasia	5	Amniotic bands	3
Hydrocephalus	1	Trombocitopenia	1
Renal	14	Disorder of Sexual Differentiation	1
Abdominal Wall defects	10	Holoprosencephaly	3

### P19 The Ten Group Classification System used to assess maternal and perinatal morbidity and mortality; a 10-year study in the Netherlands

#### Loes Monen^1^, Ida Smailbegovic^1^, Michael Robson^3^, Victor Pop^2^, Tom Hasaart^1^, Simone Kuppens^1^

##### ^1^Department of Obstetrics and Gynaecology Catharina Hospital, Eindhoven, the Netherlands; ^2^Department of Medical Health Psychology, Tilburg University, Tilburg, the Netherlands; ^3^National Maternity Hospital, Dublin, Ireland

###### **Correspondence:** Loes Monen

During recent years there has been an increase in inductions and Caesarean sections (CS). It is important to analyze if this has led to improved maternal and/or neonatal outcomes. In order to compare obstetric care, a valid and uniform classification system, such as the ‘Ten Group Classification System’ (TGCS) should be used. The TGCS has been implemented to analyze CS rates, but so far it has not been used to assess neonatal and maternal outcomes. In the current study, we have analyzed inductions, CS and maternal and neonatal outcomes.

In this retrospective cohort study, all pregnancies ≥24 weeks from 2000 to 2009, were extracted from the Netherlands Perinatal Registry (PRN), a database consisting of >95% all births in the Netherlands. For all births, neonatal and maternal outcomes were collected. All pregnancies were classified according to the TGCS. Differences were calculated using chi-square tests. For trend analyses the Cochran-Armitage test was used.

Maternal mortality did not show significant changes. In groups 1 and 2 (nulliparous women), maternal obstetric hemorrhage (>1000mL) has steadily increased over the years, from 4.4% in 2000 to 6.8% in 2009 (p < 0.001), with a similar pattern as the rise in CS rates. In all groups stillbirth rates decreased tremendously. In groups 3 and 4, postpartum hemorrhage increased from 3.1% to 4.8% (*p* < 0.001).

Maternal morbidity has increased over the years, while stillbirth rates have decreased. As a CS might also influence maternal morbidity in a possible subsequent pregnancy individualized care is very important. To analyze the effects of changes in obstetric care, it is important to collect the best quality of data. To achieve this, a system like the TGCS should be used. The TGCS can thus not only be used to analyze CS, but also for analyzing perinatal and maternal outcomes.

### P20 Unheard cries: the impact of infant loss on African American communities

#### Stacy Scott^1,2^

##### ^1^Global Infant Safe Sleep Center, Columbus, OH, USA; ^2^Baby 1st Network, Cuyahoga Falls, OH, USA

The racial disparities that exist in the United States, specifically, as it relates to prenatal, perinatal and postnatal loss cannot be ignored. Historically, African American babies die more than two to three times the rate of white babies in all categories. It also known one in 160 pregnancies ends in stillbirth for which African-American women are twice as likely to experience this type of loss. Although there is major research addressing why these type of deaths occur. This presentation will address the aftermath and impact these losses have on a segment of population who experience inequites in birth outcomes across the board.

The researcher employed a qualitative research method utilizing a phenomenological approach. Phenomenology is grounded in philosophy studying consciousness as experienced by the participant.

Analysis of the data revealed four themes common to all parents.

- Recognizing problems and responding to the loss,
- Dealing with stressful life events,
- Creating and cherishing memories of their infant, and
- Living with the loss.

Factors that influence the impact on African American parents that experience infant loss is only compounded by the lack of resources for families of color related to grief and trauma support. The goal of this research is to frame a model for addressing the gap in support services for communities who experience increased rates of infertility, miscarriages, stillbirth and infant death.

### P21 Spontaneous abortion, miscarriage and early pregnancy loss: a bibliometric analysis

#### Amanda Ross-White

##### Queen’s University, Kingston, Canada

Language is a powerful way of conveying meaning and use of specific terms can include unintended consequences. This is particularly true in medicine, where the terminology used by laypersons and professionals can differ widely, and can lead to confusion and even patient safety errors. This paper analyzes terminology used for miscarriage, early pregnancy loss and/or spontaneous abortion, revealing how its use has changed over time, resulting in a change to the Medical Subject Headings for Spontaneous Abortion beginning in January of 2018.

Using Web of Science, the author tracked publications that used the terms miscarriage, spontaneous abortion or early pregnancy loss in the keywords, titles or abstracts of scientific literature over time to determine if there has been a change in the use of these terms and whether that change is geographically based.

By the late 1990s, the term miscarriage came to be the dominant term used in the scientific literature, showing a marked increase compared to the other two terms. In separating terms by subject heading and abstract/title use, the decline in the use of the term spontaneous abortion to describe pregnancy loss in the first trimester was even more precipitous.

Based on analysis of these terms in the literature, the National Library of Medicine (US) which controls Medical Subject Headings, will be changing the entry term for Spontaneous Abortion to Miscarriage for 2018.

### P22 The Hummingbird Project: primary health care support for families pregnant after stillbirth, an intersectoral collaboration

#### Lynn Farrales^1,2,3,4^, Lee Saxell^5,6^, Jaime Ascher^3^, Zoë Hodgson^5,6^, Lora Boshoff^3^, Petra Selke^6^, Jessica Liauw^6^

##### ^1^University of British Columbia, Vancouver, Canada; ^2^Fraser Health, Burnaby, Canada; ^3^Still Life Canada: Stillbirth and Neonatal Death Education, Research and Support, Vancouver, Canada; ^4^International Stillbirth Alliance, Bristol, UK; ^5^South Community Birth Program, Vancouver, Canada; ^6^BC Women’s Hospital, Vancouver, Canada

###### **Correspondence:** Lynn Farrales

For bereaved families, pregnancy after stillbirth is often wrought with intense fear and anxiety with limited effective support and evidence to guide antenatal care. The long-term psychological outcomes after stillbirth include depression, anxiety and post-traumatic stress disorder. This study aims to: (1) describe the care and support which bereaved parents identify as important in subsequent pregnancies, (2) implement group care support for bereaved parents in their subsequent pregnancies within a primary health care context, and (3) set the groundwork for expansion of this model of care to satellite clinics.

Using principles of community-based participatory research, a research team composed of bereaved parents, midwives, nurses, family doctors and obstetricians will conduct a 3-year study. Year 1: Focus groups with bereaved families who have had subsequent pregnancies to identify important components of care and support, and construction of a curriculum for group psychosocial care. Year 2: Pilot test a curriculum for group care and support in a primary health care setting. Assess group curriculum using mixed qualitative and quantitative analyses. Year 3: Overall assessment and planning for expansion to satellite clinics.

Through focus group discussions (Year 1) and pilot testing of group sessions (Year 2), we will identify key components of effective care and support.

Our goal is to identify and develop community and psychological interventions to be integrated into clinical protocols, which enhance relevant care and support for families who are pregnant after stillbirth. It is our hope that an intersectoral team with a high index of patient involvement will help to frame a holistic approach to pregnancy after stillbirth and create the groundwork for more research in this area.

Ethical approval for the study was granted by the Behavioural Ethics Research Board of the University of British Columbia and Fraser Health Authority (Reference: H16-02671). Written informed consent will obtained for all study participants.

### P23 Cause of stillbirth in low- and middle-income countries: a multi-country study

#### Mamuda Aminu, Sarah Bar-Zeev, Sarah White, Nynke van den Broek

##### Liverpool School of Tropical Medicine, Liverpool, UK

###### **Correspondence:** Mamuda Aminu

OBJECTIVES: To assess cause of stillbirth in low- and middle-income countries (LMIC), and test the performance of computer algorithms in identifying cause of death, and to highlight vital priority areas for overall improvement in quality of care in LMIC.

DESIGN: Retrospective, observational study.

SETTINGS: 12 hospitals in Kenya, Malawi, Sierra Leone and Zimbabwe.

POPULATION: Cases of stillbirth in the selected health facilities.

METHODS: Healthcare providers (HCPs) were trained to conduct stillbirth reviews; they assigned cause of death through consensus, and collected data on cases reviewed. An expert panel reviewed the data and independently assigned cause of death. A set of computer algorithms were then used to assign cause of death for each case. Results of the three methods of cause of death assessment were analysed and compared.

MAIN OUTCOME MEASURE: Cause of stillbirth.

RESULTS: A total of 1,563 stillbirths were recorded; 1,329 (85.0%) were reviewed and 1,267 (95.3%) met the inclusion criteria. The hospital stillbirth rates for Malawi, Zimbabwe, Kenya and Sierra Leone were: 20.3, 34.7, 38.8 and 118.1 per 1,000 births, respectively. The major causes of stillbirth were: asphyxia (18.5% – 37.4%), placental disorders (8.4% – 15.1%), hypertensive disorders (5.1% – 13.6%), infections (4.3% – 9.0%), cord problems (3.3% – 6.5%), ruptured uterus (2.6% – 6.1%) and unknown (17.9% – 26.0%). The algorithms generally agreed with the expert panel (k-value = 0.34; p < 0.0005).

CONCLUSIONS: Majority of stillbirths in LMIC could be prevented with better care for all mothers and babies. HCPs should be encouraged to conduct reviews and act upon the findings to improve quality of care. Computer algorithms could complement human reviews and provide acceptable results in a research context. More research is needed to refine algorithms for facility- and community-based audits.

Our study was approved by the following ethics committees. Ethics Committee, Liverpool School of Tropical Medicine: Reference number 14.026; dated 22nd October 2014, 6th January 2015, 15th January 2015 and 19th March 2015. Kenyatta National Hospital/University of Nairobi Ethics & Research Committee; Reference number KNH-ERC/A/398; dated 23rd December, 2014. College of Medicine Research & Ethics Committee (COMREC) Malawi: Reference number P.07/14/1601; dated 15th December, 2014. Sierra Leone Ethics and Scientific Review Committee: Dated 9th October 2014 and 31st August 2015. Medical Research Council of Zimbabwe: Reference number MRCZ/A/1895; dated 9th March, 2015.

### P24 Postmortem after late stillbirth: influences on maternal decision-making in a New Zealand study

#### Robin Cronin^1^, Jane Zuccollo^2^, Minglan Li^1^, Vicki Culling^3^, John Michael David Thompson^4^, Edwin Mitchell^4^, Lesley Margaret Elizabeth McCowan^1^

##### ^1^Department of Obstetrics and Gynaecology, University of Auckland, Auckland, New Zealand; ^2^Department of Obstetrics and Gynaecology, University of Otago, Wellington, New Zealand; ^3^Vicki Culling Associates, Wellington, New Zealand; ^4^Department of Paediatrics: Child Health and Youth Health, University of Auckland, Auckland, New Zealand

###### **Correspondence:** Robin Cronin

Postmortem examination after late stillbirth is the gold standard investigation and knowing the cause of a stillbirth is important to parents. We aimed to identify factors influencing maternal decision-making about postmortem examination after late stillbirth through analysis of the New Zealand Multicentre Stillbirth Study data.

A total of 169 women with singleton pregnancy and non-anomalous late stillbirth (≥28 weeks’ gestation) from seven New Zealand health regions were interviewed within six weeks of stillbirth. We investigated decision-making about postmortem by asking participants if they would make the same decision to accept or decline a postmortem again.

All women were offered a postmortem examination. The majority (99, 58.6%) chose a full postmortem examination, 47 (27.8%) had a placental examination only, 16 (9.5%) had no investigations and 7 women (4.1%) chose a limited examination, babygram or MRI. Declining a full postmortem was associated with Maori or Pacific ethnicity (p = <0.001), parity ≥2 (p = <0.001), being unmarried (p = 0.029) and not having tertiary education (p = 0.048). The most common (47/70, 67.1%) reason for declining was that they ‘did not want the baby to be cut’. No women who consented to a full postmortem (0/99) regretted their decision but (2/99) were unsure about their choice. Ten percent (7/70) who declined a full postmortem said they would not make this decision again.

The finding that no participants in the New Zealand Multicentre Stillbirth Study regretted consenting to a full postmortem examination when interviewed in the postpartum period, but 10% regretted their decision to decline, may assist parents facing this difficult choice in the future.

Ethical approval for the study was granted by the Northern “X” Regional Ethics Committee (Reference; NTX/06/05/054). Written informed consent was obtained by all study participants.

### P25 “I’m afraid of upsetting them further”: student midwives educational needs in relation to bereavement in the maternity setting

#### Jean Doherty^1^, Sarah Cullen^1^, Brenda Casey^1^, Anne McMahon^2^, Mary Brosnan^1^, Lucille Sheehy^1^, Barbara Lloyd^2^, Theresa Barry^1^, Barbara Coughlan^2^

##### ^1^National Maternity Hospital, Dublin, Ireland; ^2^University College Dublin, Dublin, Ireland

###### **Correspondence:** Jean Doherty

Caring for bereaved parents, in the maternity setting, can be challenging for all healthcare professionals. Midwifery students are often vulnerable in their transition to becoming qualified. Being ‘sheltered’ from these situations by staff midwives and managers is quite common, exacerbating the problem. The beginning of the undergraduate midwifery program concentrates on physiological birth, with little benefit given to bereavement care in the maternity setting. It is not until the students reach their final year, that theoretical classes on grief and bereavement are given to the students. It has been questioned if this is too late, as some students end up in situations where they are left to look after bereaved parents with little or no training.

A mixed methods approach was used to evaluate the impact on students, participating on a newly developed, inter-active, bereavement care workshop. Preliminary data from a group of BSc Midwifery interns (n = 18), commencing the workshop, is presented.

89% of the students stated that lack of, or limited, exposure to bereaved parents was the main inhibitor to their confidence in supporting them. Of the respondents who answered about exposure, 6% had been in more than three situations involving bereavement, whereas 25% had not yet had any exposure. When asked what they find most difficult about caring for bereaved parents, being unsure of what to say was dominant in their responses. Other issues mentioned were the practicalities of bereavement; being afraid of upsetting parents further and how to deal with the silence in the room.

Although bereavement education is life long process, some initial findings from this study affirm the need for a more interactive approach in education and training for student midwives.

Ethical approval for this study was granted by the ethical committee of the National Maternity Hospital and University College Dublin. Written informed consent was obtained from all participants.

### P26 Bereavement care education and training in clinical practice: a workshop supporting the development of confidence and competence in midwifery students

#### Jean Doherty^1^, Brenda Casey^1^, Sarah Cullen ^1^, Anne McMahon^2^, Mary Brosnan^1^, Lucille Sheehy^1^, Barbara Lloyd^2^, Theresa Barry^1^, Barbara Coughlan^2^

##### ^1^National Maternity Hospital, Dublin, Ireland; ^2^University College Dublin, Dublin, Ireland

###### **Correspondence:** Jean Doherty

Shock, isolation, sadness, self blame and anxiety are the emotions which feature predominantly with midwives who look after families suffering bereavement through stillbirth, neonatal death and miscarriage. With 82% of healthcare professionals reporting having received no bereavement training, midwives often feel ill-equipped to cope with this aspect of their jobs. These feelings are even more prominent with student midwives, who lack the confidence needed to communicate properly with grieving parents. With this in mind, an interactive workshop was compiled to fill the competence and confidence gap in the area of bereavement education.

A review of the literature outlined the gap in bereavement education, and guided the specific topics to be covered, and the most effective educational tools to be included. Key staff members, with extensive clinical and academic experience, devised an interactive workshop. An expert panel, which included midwives, senior midwifery management, and a member of the chaplaincy team, participated in a focus group to gain their insight into the proposed content of the workshop.

The full day workshop included practical advice for students about communication – good and bad; making memories; where students can find information requested by parents; and parents’ perspectives on the impact of bereavement and how staff can help or hinder their grieving process. The workshop also included interactive role-play, which has been proven to be advantageous over didactic approaches. The final part of the workshop concentrated on the importance of self-care and included a mindfulness hour. Self-care has been advocated to increase personal coping.

The workshop is being evaluated, with the intention that it can be integrated into the curriculum going forward.

Ethical approval for this study was granted by the ethical committee of the National Maternity Hospital and University College Dublin. Written informed consent was obtained from all participants.

### P27 Improving birth outcomes: a prospective audit of the detection and management of small for gestational age (SGA) fetuses

#### Claire Dougan^1^, Alyson Hunter^1^, Stan Craig^1^, Dale Spence^2^, Emma Mc Call^2^, Emily Bailie^1^, Sunneva Gilmore^1^, Naomi Harvey^1^, Nazish Kanwal^1^

##### ^1^Royal Jubilee Maternity Hospital, Belfast Health and Social Care Trust (BH&SCT), Belfast, N. Ireland; ^2^Queen’s University, Belfast, N. Ireland

###### **Correspondence:** Claire Dougan

Stillbirth rates in the UK are one of the highest in the developed world. Using ReCoDe classification, 43% of stillbirths can be attributed to intrauterine growth restriction(IUGR). Small for gestational age(SGA); <10th centile on a customized growth chart, is synonymous with IUGR. Risk of stillbirth is reduced when IUGR is detected antenatally compared to undetected. Royal College of Obstetricians and Gynaecologists(RCOG) 2013 guidance stratifies antenatal care in those at risk of SGA.

Objectives:

- Determine whether women identified ‘at risk of SGA’ receive appropriate antenatal care according to RCOG guidance
- Compare detection rates of SGA in women who received appropriate vs. inappropriate antenatal care according to RCOG guidance;
- Compare intrapartum management and perinatal outcomes where SGA was detected vs. undetected antenatally.

We prospectively collected anonymised data for 494 consecutive singleton deliveries in BH&SCT (8.6% of annual singleton births). Clinical risk for SGA, birth weight, antenatal and intrapartum management and birth outcome data were analyzed using “IBM - Statistical analysis software package - SPSS Statistics.”

In total, 33% (165/494) of women were categorized at risk of SGA in accordance with RCOG guidance (minor 42, major 123). Overall, 56% (91/163, 2NA) were managed appropriately antenatally, with 65% (79 of 121, 2 NA) and 29% (12/42) in major and minor risk groups respectively (see Table 4). Across all categories (including ‘low risk) 11% (56/493, 1 missing) of babies were SGA at birth with a 55% (30/55, 1 missing) antenatal detection rate. SGA babies detected antenatally were on average delivered 13 days earlier than their undiagnosed counterparts. A higher proportion were delivered by caesarean section (50% vs 32%),

Our data suggests detection of SGA results in earlier delivery and changes the mode of delivery. Further research is required to ascertain whether antenatal detection of SGA impacts intrapartum management and perinatal outcomes.

**Table 4 (abstract P27).** See text for description

	Care received	<10^th^ centile at birth	SGA detected antenatally	stillbirth	Apgar <7 @5mins	Neonatal unit admission
Low risk N = 329		25/328 (8%)	Yes 10 (42%)	0	0	2
			No: 14 (58%)	0	1	2
		x1 missing data	X1 missing data			
Minor risk N = 42	Inappropriate 30/42 (71%)	2/30 (7%)	Yes: 2/2 (100%)	0	0	0
			No: 0	N/A	N/A	N/A
	Appropriate 12/42 (29%)	2/12 (16%)	Yes: 0	N/A	N/A	N/A
			No: 2/2 (100%)	0	1	0
Major risk N = 123	Inappropriate 42/121 (35%)	6/42 (14%)	Yes: 4 (67%)	0	0	1
			No: 2 (33%)	1	0	0
	2-late bookers				x1 missing data	
	Appropriate 79/121 (65%) 2- late bookers	21/79 (27%)	Yes: 14/21 (67%)	0	0	1
			No 7/21 (33%)	0	0	1

### P28 Translating lessons learnt from perinatal deaths into policies, programs and practice: perinatal death audits in Sri Lanka

#### Kapila Jayaratne

##### Family Health Bureau, Colombo 10, Sri Lanka

With the reduction of infant mortality rate to a single digit (8.2 per 1000 live births) and a larger proportion (60%) of such deaths being early neonatal deaths (END), perinatal deaths (PND) emerge as a priority area in Sri Lanka -a low and middle income country in South Asia. We aimed to analyze national PND data for action.

National Perinatal Mortality Surveillance system was implemented in 2006. All specialized hospitals (with an obstetrician/paediatrician) are required to document all PNDs, review them monthly at a hospital stakeholder meeting and send a report to Ministry of Health. Structured data collection formats, monthly reporting formats and guidelines are available.

In 2014, data were received from all hospitals (government and private sector) with labour rooms from a total of 452 hospitals (74 Specialized government hospitals, 357 Non-specialized peripheral hospitals and 21 Private hospitals). Considering total live births reported by Civil Registration System (349715), coverage of live births in this study was 99.6% (n = 348362).

The analysis included 1386 (46%) stillbirths and 1623 (54%) ENDs (Total 3009 PNDs). Stillbirth and early neonatal mortality rates were 4.0 per 1000 total births and 4.7 per 1000 live births respectively. The perinatal mortality rate was 8.6 per 1000 total births. Majority of PNDs (52.1%) were male. One third (35.7%) of PNDs occurred in primies. Birth weight < 1000g was reported in 20% of all PNDs. Nearly 10% of ENDs had a period of gestation.

Outcomes were translated into action at hospital, district and national levels; prevention strategies on premature deliveries, expansion of premature baby units, strategies on birth defects prevention, early neuro-developmental care, neonatal retrieval systems and introducing therapeutic body cooling.

Sri Lanka being a low and middle income country implements an organized PND audit system to translate the outcome for meaningful interventions.

### P29 The importance of clear inter-professional communication following stillbirth in the hospital environment

#### Vivienne Manley

##### Belfast Health & Social Care Trust, Belfast, N. Ireland

Clear visual, verbal and written communication are vital to assist the various professionals working in the care of parents following stillbirth so that the most appropriate and sensitive care is given.

This is a verbatim case-review of a chaplaincy encounter following stillbirth where a chaplain did not receive adequate information. This verbatim highlights three aspects of communication and how it can be enhanced.

Verbal communication

Professionals who are not permanent ward staff may not receive adequate information and thereby patient care may be affected. Extra care must be taken to communicate verbally.

Visual communication

A visual sign was not used in this instance to alert the professional to a death having occurred on the ward. This would have been very beneficial to all.

Written communication

Written information could have been offered to the parents at the time of still birth and so enhance the spiritual and pastoral value of the pastoral visit.

A newly written booklet for parents following stillbirth, and other distressing times, entitled “When hopes seem dashed” has been produced, and is available in the Royal Jubilee Maternity hospital of the Belfast Trust. This will enable the availability of spiritual care via a hospital chaplain to be offered and the pastoral encounter enhanced.

This study highlights the importance of clear, verbal, visual and written communication between healthcare professionals around the time of stillbirth in the hospital environment. Appropriate communication could have enabled the chaplain to be better prepared and additional written information could have enhanced the spiritual and pastoral care of the parents. The study also emphasises that reflection on a verbatim case review is a valuable tool in the learning process.

### P30 Placental changes in diabetic stillbirths

#### Daniel Shingleton^1^, Alexander Heazell^1,2^, Gauri Batra^1^

##### ^1^Central Manchester University Hospitals NHS Foundation Trust, Manchester, UK; ^2^Manchester University, Manchester, UK

###### **Correspondence:** Daniel Shingleton

Stillbirth has numerous associated risk factors and placental pathologies. However, the predictive value of these factors is low as they are also seen in live births. One such factor is maternal diabetes which is associated with a 4-fold increased risk of stillbirth. This study aimed to elucidate the spectrum of placental pathology associated with diabetes, and whether this is more profound in diabetic pregnancies which end in stillbirth.

Histological techniques were carried out on slides cut from representative blocks of placental parenchyma from different clinical conditions; normal live births, diabetic live births, unexplained stillbirths and diabetic stillbirths. Photomicrographs were taken and examined to assess placental vascularity (CD31), proliferative index (Ki67), syncytial nuclear aggregates (H&E). cytokeratin area/nuclear area (CK7) and avascular villi (CD31). Histopathological data were extracted from clinical reports.

Stillbirths had an increased CK7 area compared to normal live born cases with unexplained stillbirths being significantly raised compared to live births (p = 0.005) and diabetic stillbirths significantly raised compared to diabetic live births (p = 0.0008). Proliferative index was significantly lowered in cases of unexplained stillbirth compared to normal live born cases (p = 0.04). There was no difference in placental vascularisation in diabetes. Additionally, 80% of unexplained stillbirth placenta were classified as normal and 90% of diabetic stillbirth placenta presented with maturation disturbance based on histopathological examination.

Diabetic stillbirths have similar phenotype to that of live born controls, however, the increased CK7 area of diabetic stillbirths when compared to diabetic live births points towards the role of increased distance between fetal capillary walls and the trophoblast basement membrane playing a crucial role in outcome when pregnancy approaches term. Data gathered from unexplained stillbirths indicates an unknown mechanism for stillbirth or a non-placental cause in these cases that cannot be determined by routine histopathological examination alone.

Ethical approval for the research was sought for and granted by the proportional review sub-committee of the West Midlands – South Birmingham Research ethics committee (16/WM/0372) on the 16th August 2016. The patients whose tissue samples were used in this study had previously given consent for their use in research during either the consent for a post mortem examination of a stillborn child or during the biobank consent process for liveborn cases.

### P31 Analysis of missing data for stillbirths in a retrospective register review in six facilities across in Tanzania, Bangladesh and Nepal

#### Kyohei Takano, Hannah Blencowe

##### London School of Hygiene and Tropical Medicine, London, UK

###### **Correspondence:** Kyohei Takano

An estimated 2.6 million stillbirths occur annually and half of all stillbirths are estimated to occur during the intrapartum period. Although effective prevention needs a global strategy based on knowledge of risk factors for intrapartum stillbirths, the quality of data on intrapartum stillbirths, which have not been routinely collated in many low-income countries, tends to be poor with some data missing. The ability of study to accurately conduct analysis is dependent upon the degree of missingness of data and how the missingness of data is addressed. An overall aim of this project is to explore missingness of data in routine labour ward registers regarding stillbirths.

A retrospective register review of data collected on birth outcomes over a 1-year period was undertaken for women delivering in six health facilities across 3 countries. Descriptive analysis of the available data will be undertaken, including the prevalence of adverse birth outcomes and the levels and types of missingness of data. The impact of missing data on estimates of prevalence of stillbirth and the proportion intrapartum and antepartum will be assessed, using multiple imputations where appropriate. Data analysis is currently underway.

Labour ward registers are an important source of routinely collected data for facility births, missingness of data, and lack of collation of relevant stillbirths currently impede their use for tracking stillbirth outcomes. Improved end-user input in design and ownership of labour ward registers may improve the quality and use of data locally.

Ethical approval for the study was granted by the MSc Research Ethics Committee of London School of Hygiene and Tropical Medicine (Reference; 13581).

### P32 Getting a better picture: a prospective audit on the accuracy in ultrasound-detection in patients managed as small for gestational age

#### Sunneva Gilmore, Claire Dougan

##### Belfast Health and Social Care Trust, Belfast, Northern Ireland

###### **Correspondence:** Sunneva Gilmore

Placental insufficiency and intrauterine growth restriction are leading causes of stillbirth. To identify the pathologically small fetus for gestational age (SGA), an ultrasound estimated fetal weight (EFW) is referenced against a customised individual growth chart. This audit examined the accuracy of ultrasound-detected SGA compared with actual birth-weight (ABW) and birth-centile, in fetuses managed by delivery in a maternity unit.

A prospective audit was conducted for patients with singleton intrauterine pregnancies, who were induced or scheduled an elective caesarean section (ELCS) for the primary indication of SGA, in a maternity tertiary unit from 1st-30th September 2016. Data was collected within a teaching-hospital employing regular trainee competency assessments in ultrasonography.

In total, 6% (30/494) of deliveries underwent induction/ELCS for suspected SGA, defined as EFW below the tenth-centile on the customary growth chart. Despite this, three fetuses were on the tenth-centile. The gestational age was equal or greater than 37weeks in 90.3% (range 26weeks + 4day—40weeks + 3 days). The scan to delivery interval averaged at 5.3 days.

The birth-centile derived from the ABW detected 56.6% (17/30) with SGA appropriately. 43.4% were managed for SGA, but were not SGA based on birth-centile. Among these (13/30), 46% had an ultrasound difference of >10%, compared with 11.7% in those with birth-centile SGA. Emergency caesarean-section was similar in both groups (38% v 41%). In this audit, those with a higher ultrasound margin of error, were more likely to be above the tenth-birth-centile (Table 5).

This study reveals a discrepancy between estimated and birth-centile in a significant proportion of patients. However, in most cases the ABW is within a 10% ultrasound-margin of error. This is similar to other studies. A larger study would better inform on the accuracy of ultrasound-detection rates, establish an acceptable margin of error to improve standards, and inform rates of inappropriate medicalization in misdiagnosed SGA.

Ethical approval for the project was not required for audit purposes, however data was collected within an ethical framework.

**Table 5 (abstract P32).** Accuracy of ultrasound estimation in patients managed as small for gestational age

**Proportion of error within (%)**	**+/- 10%**	**+/- 15%**	**+/- 20%**
All Patients (30)	73.3 (22)	86.7 (26)	93.3 (28)
Gestational Age <37weeks	66.6 (2)	100 (3)	100 (3)
Gestational age >37weeks	74.1 (20)	92.5 (25)	92.5 (25)
Mean percentage difference when Birth Weight <10^th^	5.5%		
Birth weight >10th	11.1%		

### P33 Giving sleep position advice in pregnancy: will we make women anxious?

#### Jane Warland, Georgie Beaufoy, Jill Dorrian

##### Unisa, Adelaide, Australia

###### **Correspondence:** Jane Warland

Over the past decade there has been emerging evidence that suggests that maternal sleep position may be associated with stillbiirth. It has been postulated that approximately one quarter, to one third of stillbirths might be prevented by simply asking women to change their sleep position. However, there are concerns that giving women information about sleep position and stillbirth risk may make them anxious. This study aimed to determine underlying messages being conveyed to pregnant women especially with respect to sleeping position during pregnancy in order to understand any anxiety associated with giving this message.

An online survey of 537 Australian women (107 of whom were “currently pregnant”). The survey examined participant’s views regarding sleep position messages, type of information source, and pre-existing knowledge. Participant characteristics such as general anxiety and the ‘Fetal Health Locus of Control’ Scale were also collected.

The results showed that of the participants who were currently pregnant 65% (n = 66) settled to sleep on their left side, whereas only 23% (n = 23) said they settled in this position when not pregnant. When asked on a 5 point Likert scale how anxious they may have been about sleep position in their current pregnancy 85% held a “neutral” to “not at all anxious” view. Data analysis is still underway examining the correlation between state trait anxiety, fetal locus of control and any anxiety about the sleep position message in the pregnant group and these results will be presented at the conference.

This study indicates that many pregnant women were already changing their sleeping position in pregnancy. A small subset of pregnant women may feel anxiety associated with the sleep position in pregnancy message and therefore some care needs to be taken to inform women about the importance of sleep position without unduly provoking anxiety.

Ethical approval for the study was granted by The Human Research Ethics Committee of the University of South Australia (Reference; 0033096). Written informed consent was obtained by all study participants.

### P34 Withdrawn

### P35 Perinatal mortality MDT meetings: attendance in a tertiary university maternity hospital

#### Rama Akachuku, Keelin O’donoghue

##### Cork University Maternity Hospital, Wilton Cork, Ireland

###### **Correspondence:** Rama Akachuku

Perinatal deaths should be reviewed at perinatal mortality meetings. The perinatal mortality multidisciplinary team (PM-MDT) meeting is a vital forum for communication between clinicians as it ensures understanding of individual cases and facilitates appropriate follow-up. These meetings are also a valuable forum for learning for the wider disciplinary team. The aim of this project was to review the attendance of healthcare staff from a single maternity unit at PM-MDT meetings.

Our perinatal pathology service was established with the appointment of a perinatal pathologist in 2012. All hospital staff are notified about clinical/academic meetings, including PM-MDT meetings. We examined attendance records for the PM-MDT meetings from 2013 to 2016, and compared this with numbers of staff employed in the various disciplines.

There were 31 PM-MDT meetings held from 2013 to 2016 (median,8; range 6-9 per year). Those who attended included consultant obstetricians and neonatologists, doctors-in-training, midwife-specialists in bereavement/loss, clinical-midwife-managers in pregnancy loss and quality/patient safety, medical students and healthcare chaplains. Numbers of staff at the MDT meetings ranged from an average of 14 per meeting in 2013 to 13 in 2016 (range;7-17). Only 7 consultant obstetricians (7/16;44%) ever attended the meeting, although 3 consultants only attended on one occasion. Among neonatology consultants, one attended each meeting, although only for discussion of neonatal deaths. No representatives from senior midwifery management (including the Director of Midwifery) or administration (including the Hospital Manager) ever attended the meetings, and there were no staff attendees from the labour ward, ultrasound or out-patients department.

Perinatal deaths should be reviewed formally at PM- MDT meetings in maternity units. All healthcare staff have a responsibility to attend. We need to examine the reasons why attendance at PM-MDT meetings in our unit is poor, particularly among senior clinicians, labour-ward midwives, midwifery educators, and management.

### P36 Factors associated with stillbirth autopsy rates in Georgia and Utah, 2010-2014: the importance of delivery location

#### Katie Forsberg, Lauren Christiansen-Lindquist

##### Emory University Rollins School of Public Health, Atlanta, USA

###### **Correspondence:** Katie Forsberg; Lauren Christiansen-Lindquist

As the gold standard for determining the cause(s) of stillbirth, autopsies can help the grieving process, inform the management of current and future maternal care, and foster new interventions. However, stillbirth autopsies are underused in the United States, and little is known about what factors are associated with their receipt. This study aimed to determine whether demographic, operational, and medical factors are associated with the performance of stillbirth autopsies in Georgia and Utah.

Using Georgia and Utah fetal death certificates from 2010-2014, we evaluated the relationship between demographic, operational, and medical factors and stillbirth autopsy performance. Analysis was conducted using logistic regression with a predicted margins approach. Each state was analyzed separately.

The stillbirth autopsy rate was low (11.9% in Georgia (N = 5,610) and 23.9% in Utah (N = 1,425)). In Utah, the autopsy rate significantly decreased during the study period (p = 0.01). Stillbirths delivered outside of large metropolitan areas were less likely to receive an autopsy (medium/small metropolitans: prevalence ratio_GA [PR] = 0.57, 95% confidence interval [CI]: 0.48, 0.68 and PR_UT = 0.48, CI: 0.38, 0.59; nonmetropolitans: PR_GA = 0.57, CI: 0.43, 0.75 and PR_UT = 0.37, CI: 0.21, 0.63). In Georgia, autopsies were less common among stillbirths of Hispanic (vs. white) women women (PR = 0.57, CI: 0.41, 0.79), of earlier than later gestational ages (PR = 0.59, CI: 0.51, 0.69) and of multiple birth pregnancies (PR = 0.71, CI: 0.53, 0.96).

Despite strong evidence supporting the value of stillbirth autopsies, autopsy rates were low in Georgia and Utah. Approximately half of the stillbirths were delivered outside of large metropolitan areas, and this population may be particularly underserved. Additional research is needed to determine whether autopsies were not performed because they were not offered or because parental consent was not given.

This research was approved by the Institutional Review Boards of Emory University and Georgia Department of Public Health. Utah Department of Health did not require formal Institutional Review Board approval, but reviewed our study and executed a data sharing agreement, which was submitted to the Emory University Institutional Review Board

### P37 Developing and characterising animal models of stillbirth

#### Samantha Lean^1^, Rebecca Jones^1^, Mark Dilworth^1^, Esther Aiyelaagbe^1,2^, Kathryn Hentges^2^, Alexander Heazell^1^

##### ^1^Division of Developmental Biology and Medicine, University Of Mancheter, Manchester, UK; ^2^Division of Evolution & Genomic Sciences, University of Manchester, Manchester, UK

###### **Correspondence:** Samantha Lean

Investigating stillbirth in human pregnancies using laboratory approaches is challenging. Tissues are usually obtained several days after fetal death and analyses are largely limited to retrospective histopathological studies. Alternatively, pregnancies at high risk of stillbirth are studied it is difficult to know which infants would have been stillborn without intervention. There is a critical need for alternative approaches to study stillbirth. Animal models have been valuable to study and develop therapeutic strategies in other pregnancy complications. We have aimed to develop murine models of in utero fetal death to advance the understanding and prevention of stillbirth.

We have developed and characterised two mouse models of stillbirth. The first is a model of placental-related, late gestation stillbirth that involved ageing virgin female mice to 38 weeks (equivalent to ~40 years in humans) before pregnancy (controls are 8-12 weeks old). 56% of the resulting pups are growth restricted and 14% die in late pregnancy; the pups that die are significantly smaller than those that survive. Growth restriction and stillbirth in this model is associated with placental dysfunction.

The second model involves a mutation in the gene ErbB2 which causes sudden mid-gestation stillbirth in the absence of placental causes. We identified cardiac dysfunction in these fetuses, which parallels with sudden infant death syndrome, and altered expression of key target cardiac ion channel genes that may be responsible for in fetal demise secondary to cardiac pathology. These studies offer target genes to investigate in currently unexplained stillbirths.

These models provide new platforms in which to investigate stillbirths due to: a) placental dysfunction, thereby representing >40% of stillbirths in human pregnancies, or b) genetic cardiac pathologies. Furthermore, with these models we will be able to test risk stratification and therapeutic interventions. Ultimately, these studies will form the foundation for future human studies.

**Table 6 (abstract P37).** See text for description

	**2014**	**2015**	**2016**
FETAL MORTALITY RATE (x 1000)	5.9	6.1	22.1
BIRTHS (N)	663	648	619
INTRA UTERINE FETAL DEATHS (N)	4	4	14
MATERNAL AGE (YEARS)	29	23	25.8
GESTACIONAL AGE (WEEKS)	34.2	35	31.3
WEIGHT (GRAMS)	1980	2536.2	1686.4
PREVIOUS ABORTIONS (N)	1	0	2
PREVIOUS PREGNANCIES (N)	1.5	1.5	2.5
VAGINAL DELIVERY (N)	1	1	6
CESAREAN SECTION (N)	3	3	8
CONGENITAL ANOMALIES (N)	1	0	3
MATERNAL PATHOLOGIES (N)	1	1	7

All animal husbandry and experimental practices were carried out in accordance with the UK Animals (Scientific Procedures) Act 1986 under Home Office Licence 40/3385 and 70/8504. The Local Ethical Review Process of the University of Manchester approved all protocols

### P38 Frequency of intra-uterine fetal deaths in Zonal hospital of Puerto Madryn (Argentina) during the period 2014-2016

#### Maria Soledad Silva, Damián Leonardo Taire

##### Zonal Hospital “Dr. Andrés R. Isola”, Puerto Madryn, Argentina

###### **Correspondence:** Maria Soledad Silva

The Zonal Hospital “Dr. Andrés R. Isola”, serves an estimated population of 100.000 habitants. About 700 births occur in the hospital each year. The objective of this presentation is to determine the frequency of Intra Uterine Fetal Deaths (IUFD) in hospital births during the years 2014, 2015 and 2016.

Retrospective, longitudinal, observational and descriptive design. The documentation consulted is compiled in the Births-Book, and in the Monthly Obstetric Summary. The data analyzed in both documents are: gestational age, birth weight in grams, condition at birth, end of pregnancy, previous pregnancies, sex, congenital anomalies and maternal pathologies. In this series, the IUFD refers to all losses of 22 or more weeks of gestation.

The occurrence of 22 IUFD was observed from January 2014 to December 2016, out of a total of 1930 live births. The Fetal Mortality Rate was 5.9 (2014), 6.1 (2015) and 22.1 (2016) (see Table 7).The mean gestational age was 32.5 weeks. The average weight was 1894 grams. The mean maternal age was 25.9 years, multigravidae (2.1 previous pregnancies), way of ending pregnancy was cesarean section 63.6% and vaginal delivery 36.3%.

With respect to the sex of IUFD: female sex 40.9%, male sex 50% and undetermined 9%. Congenital anomalies (CA) were observed in 4 dead fetuses (18.1%). They correspond to Potter sequence, trisomy 18 phenotype, unclassified CA.

The maternal pathologies associated were: cholestasis 4.5%, placental detachment normoincerta 13.6%, pregnancy-induced hypertension 13.6%, chorioamnionitis 4.5%, gestational diabetes 4.5% and syphilis 9%.

The frequency of IUFD in our series demonstrates an increase in cases during the year 2016 with the same number of births. It is inferred the need for early detection of maternal risk factors, to avoid IUFD. The collection of data is essential to facilitate the monitoring of the content and quality of care during pregnancy and childbirth.

Ethical approval for the study was granted by the Bioethics Committee Hospital Zonal de Trelew

**Table 7 (abstract P38).** See text for description

	**2014**	**2015**	**2016**
FETAL MORTALITY RATE (x 1000)	5.9	6.1	22.1
BIRTHS (N)	663	648	619
INTRA UTERINE FETAL DEATHS (N)	4	4	14
MATERNAL AGE (YEARS)	29	23	25.8
GESTACIONAL AGE (WEEKS)	34.2	35	31.3
WEIGHT (GRAMS)	1980	2536.2	1686.4
PREVIOUS ABORTIONS (N)	1	0	2
PREVIOUS PREGNANCIES (N)	1.5	1.5	2.5
VAGINAL DELIVERY (N)	1	1	6
CESAREAN SECTION (N)	3	3	8
CONGENITAL ANOMALIES (N)	1	0	3
MATERNAL PATHOLOGIES (N)	1	1	7

### P39 Registry of post-neonatal home deaths in Chubut (Argentina) during the period 2011-2015

#### Damian Leonardo Taire, María Soledad Silva

##### Zonal Hospital “Dr.. Andrés R. Isola”, Puerto Madryn, Argentina

###### **Correspondence:** Damian Leonardo Taire

In post-neonatal mortality, occurring from 28 days to the year of life, environmental and socioeconomic conditions have a greater impact. The estimated population (2017) of the province of Chubut is 587.956 habitants. Chubut (6.9) is the second district of the country with the lowest infant mortality per 1000 live births (2015). About 9.887 newborns are born in the province every year. The objective of this presentation is to describe the incidence of Post-Neonatal Deaths (PD) in Chubut and the percentage of these deaths produced at home during the years 2011, 2012, 2013, 2014 and 2015.

Retrospective, longitudinal, observational and descriptive design. The documentation consulted is compiled by the National Direction of Statistics and Information in Health of the Ministry of Health of the Nation. This publication called Vital Statistics contains basic statistical information on deaths for the country as a whole and by jurisdiction.

**Fig. 3 (abstract P39).**
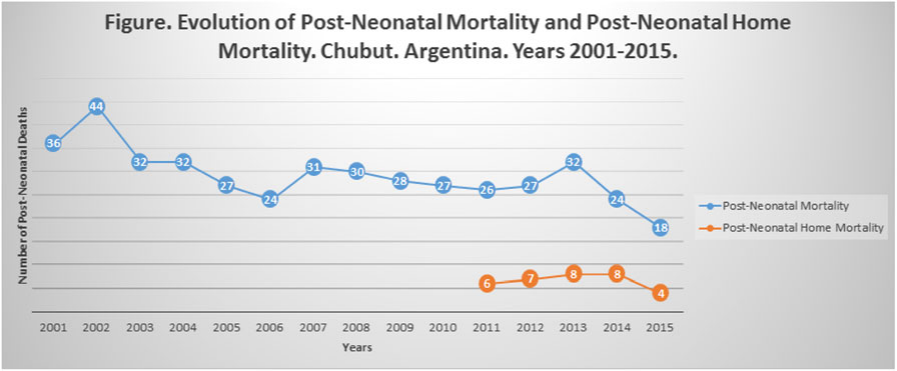
See text for description

In Chubut, in 2011, 2012, 2013, 2014 and 2015 there were 26, 27, 32, 24 and 18 PD, respectively. The 23.0% (2011), 25.9% (2012), 25% (2013), 33.3% (2014) and 22.2% (2015) deaths occurred at home (Fig. 3).

In the last 5 years, there has been a stable number of PD with an average of 25.4 deaths per year, with a decrease from 2015. It has been observed in the study period that the only year where there is no data from the PD on the place of occurrence is in 2011 (1 death without data). Based on these data, it could be inferred that a better preparation of the Statistical Reports of Death has resulted in an improvement in the registry of household mortality in Chubut.

Ethical approval for the study was granted by the Bioethics Committee Hospital Zonal de Trelew

### P40 Raised fasting plasma glucose and diagnosis of gestational diabetes in relation to risk of late stillbirth

#### Tomasina Stacey^1^, Peter Tennant^1^, Miglan Li^2^, Edwin Mitchell^3^, Lesley McCowan^2^, John Thompson^3^, Jayne Budd^4^, Bill Martin^5^, Devender Roberts^6^, Alexander Heazell^4,7^

##### ^1^School of Healthcare, University Of Leeds, Leeds, UK; ^2^Department of Obstetrics and Gynaecology, University of Auckland, Auckland, New Zealand; ^3^Department of Pediatrics, University of Auckland, Auckland, New Zealand; ^4^St. Mary’s Hospital, Central Manchester University Hospitals NHS Foundation Trust, Manchester, UK; ^5^Birmingham Women’s Hospital NHS Foundation Trust, Birmingham, UK; ^6^Liverpool Women’s Hospital NHS Foundation Trust, Liverpool, UK; ^7^Maternal and Fetal Health Research Centre, University of Manchester, Manchester, UK

###### **Correspondence:** Tomasina Stacey; Peter Tennant

The UK has one of the highest rates of stillbirth in Europe. To address this, one recommendation from MBRRACE-UK was increased focus on detection and management of gestational diabetes (GDM). The 2015 NICE guidelines recommended GDM be diagnosed by either fasting plasma glucose (FPG) ≥5.6mmol/L or 2-hour oral glucose tolerance test (OGTT) ≥7.8mmol/L. Use of FPG however remains sporadic. This study examined the joint effects of FPG levels and formal diagnosis of GDM by OGTT on stillbirth risk.

The Midlands and North of England Stillbirth Study (MiNESS) is a case-control study of non-anomalous singleton pregnancies between April 2014 and March 2016 within 41 maternity units in England. 291 women who recently experienced a late stillbirth (≥28 weeks’ gestation) and 733 controls (matched on NHS Trust and gestation) were recruited. Data were collected on various demographic, health, and lifestyle factors. 355 women were screened for GDM (89 cases and 266 controls) and had information on their FPG and OGTT results.

35 of the 355 (9.9%) women with complete screening information were diagnosed with GDM. Women with a raised FPG (≥5.6mmol/L) and a GDM diagnosis experienced similar odds of late stillbirth (OR = 1.52, 95% CI = 0.49-4.73) to women with a normal FPG and no GDM diagnosis. Women with raised FPG but no GDM diagnosis (and hence did not receive specialist care) however experienced 5.32 (95% CI = 1.44-19.72) times greater odds. This was unaffected by adjusting for ethnicity, BMI, age, and education.

A raised FPG is associated with an increased risk of late stillbirth that is largely ameliorated by a formal diagnosis with GDM, reflecting the benefits of receiving current pathways of care for GDM. Women with a raised FPG but who are not diagnosed with GDM (e.g. due to a normal 2-hour OGTT result) have a substantially increased risk of stillbirth.

Ethical approval for the study was granted by Greater Manchester Central Research Ethics Committee (Reference 13/NW/0874). Written informed consent was obtained by all study participants.

### P41 Patterns of fetal movement and the association with late stillbirth

#### Alexander Heazell^2,3^, Minglan Li^1^, Jayne Budd^3^, Robin Cronin^1^, Billie Bradford^1^, Lesley M. E. McCowan^1^, Edwin A. Mitchell^4^, Tomasina Stacey^5^, Bill Martin^6^, Devander Roberts^7^, John M. D. Thompson^1,4^

##### ^1^Department of Obstetrics and Gynaecology, University Of Auckland, Auckland, New Zealand; ^2^Maternal and Fetal Health Research Centre, School of Medical Sciences, Faculty of Biological, Medical and Human Sciences, University of Manchester, Manchester, UK; ^3^St. Mary’s Hospital, Central Manchester University Hospitals NHS Foundation Trust, Manchester Academic Health Science Centre, Manchester, Manchester, UK; ^4^Department of Paediatrics: Child Health and Youth Health, University of Auckland, Auckland, New Zealand; ^5^School of Healthcare, University of Leeds, Leeds, UK; ^6^Birmingham Women’s Hospital NHS Foundation Trust, Edgbaston, Birmingham, UK; ^7^Liverpool Women’s Hospital NHS Foundation Trust, Liverpool, UK

###### **Correspondence:** Alexander Heazell; John M. D. Thompson

Reduced frequency and intensity of fetal movements in late gestation has been associated with stillbirth. In the Midlands and North of England Stillbirth Study (MINESS) we investigated changes in fetal movements and their association with late (> = 28 weeks) stillbirth.

The MINESS case-control study was carried out over two years from April 2014 to March 2016. It recruited and interviewed 291 cases (mothers with late stillbirths) and 733 control mothers (pregnant women with ongoing pregnancies), frequency matched for maternity unit and gestation. Using a structured questionnaire data were collected about fetal movements, in particular relating to the strength and frequency during the last two weeks. Information was also collected about hiccups.

Control women most commonly reported an increase in the strength of movements in the last 2 weeks (62%), few reported a decrease (7%). For frequency staying the same was the most common response (54%) followed by an increase (25%) with few reporting decreases (9%). Cases were less likely to report increases in strength or frequency (18% and 13% respectively) and more likely to report decreases (21% and 30% respectively). In multivariable analyses, compared with those who reported no change in strength or frequency of movements in the last two weeks, those with increased strength were at a decreased risk of having a late stillbirth OR = 0.18 95%CI = (0.13,0.26). Those with decreased frequency (with no increase in strength) of fetal movements were at an increased risk OR = 3.45 95%CI = (2.20,5.43). Women who felt hiccups on a daily basis were also at reduced risk OR = 0.31 95%CI = (0.17, 0.56).

An increase in the strength of fetal movements is the norm in late pregnancy and clinical guidelines should be updated to reflect this. Patterns of fetal movements are important in predicting stillbirth, and strength is as important as frequency.

Ethical approval for the study was granted by Greater Manchester Central Research Ethics Committee (Reference 13/NW/0874). Written informed consent was obtained by all study participants.

### P42 Perinatal mortality audit of last 5 years at Bolton NHS Foundation Trust

#### Priti Wuppalapati, Joanna Smith, Neeraja Singh

##### Bolton NHS Foundation Trust, Bolton, UK

###### **Correspondence:** Priti Wuppalapati

Yearly audit of perinatal mortality since 2008-2009 but easily comparable data since 2011

All stillbirths and early neonatal death (NND) in 2014 identified databases. Information gathered from casenotes, Euroking, Badger, perinatal meeting minutes and stillbirth proformas. Compiled on excel spreadsheet and analysed.

Crude stillbirth rate 4.4/1000 in 2015 which has improved from previous year and lower than UK adjusted average for similar Trust. Main causes of death was Intrauterine Growth Restriction (IUGR) +/- placental insufficiency (1/3 of cases)

IUGR contributed to cause of death in 2/3 of cases. More than 1/3 of cases were missed IUGR. However, ongoing reducing trend of missed IUGR cases compared to previous years. 1 case of grade 3 care and 8 cases of grade 2 care – 1/3 of cases of suboptimal care. About 1 in 4 stillbirths not reported as clinical incident. About 9 in 10 women did not complete all investigations recommended in Stillbirth Pathway

Almost 8 in 10 women who required thrombophilia screen did not have this fully completed. Contraception often not discussed at postnatal review.

Recommendations: continue Growth Assessment Protocol (GAP) training and case reviews of missed Small for Gestational Age(SGA) to improve detection of Fetal Growth Restriction (FGR).

Medical review when stillbirth diagnosed to outline investigations to be carried out in management plan + review investigations ordered before discharge.

Thrombophilia results to be reviewed at postnatal review or arranged/repeated if necessary

Increased awareness to follow recommendations in Stillbirth Pathway and ensure Pathway completed – both doctors and midwifery colleagues (including in postnatal review - to include discussion of contraception)

### P43 How accurate is assessment of foetal weight in obese women?

#### Natasha Mitchell, Nabila Kalar, Kavita Verma

##### Scarborough District Hospital, Scarborough, UK

###### **Correspondence:** Natasha Mitchell

Obesity is increasingly common in the obstetric population and increases the risk of adverse pregnancy outcomes. This includes an increased risk of SGA by 50%, and significantly stillbirth, with estimated odds ratio of 1.43 . Obesity makes palpation of symphysis fundal height more difficult. Recent studies suggest however is does not affect the accuracy of ultrasound derived estimated foetal weight (EFW) but this is not routinely done in many units for all women with a BMI above 30. This study looked at the accuracy of both symphysis fundal height and ultrasound derived EFW with actual weight at delivery in a district general hospital.

38 women were prospectively identified with a singleton pregnancy and a BMI of more than 30. Symphysis fundal height was plotted on customised growth charts in addition to EFW on ultrasound. These were compared to neonatal weight at delivery. Data was analysed using ANOVA to compute F and p-values with a Tukey post hoc test to verify differences.

There was a non-significant difference between estimated foetal weight for serial ultrasound scan and actual foetal weight at birth. The difference between actual foetal weight and estimated foetal weight by symphysis fundal height was statistically different with p = 0.0001. As expected, EFW by fundal height was higher with an average difference of 788 grams.

This study found that estimated foetal weight on ultrasound in our unit was accurate at predicting estimated foetal weight in high BMI mothers. However Symphisis fundal height measurements were inaccurate often over estimating foetal growth. This may mean if pregnancies are monitored using solely fundal height measurements SGA foetuses may be missed especially those suffering from early IUGR as there may be no previous measurements to compare velocity.

### P44 Use of an integrated care pathway to improve care for women who present with intrauterine fetal death and experience stillbirth

#### John Tomlinson^1,3^, Alexander Heazell^1,2^, Karen Bancroft^1,3^, Elizabeth Martindale^1,4^

##### ^1^Greater Manchester and East Cheshire Special Interest Group into Stillbirth, Manchester, UK; ^2^Maternal and Fetal Health Research Centre, University of Manchester, UK; ^3^Bolton NHS Foundation Trust, Bolton, UK; ^4^East Lancashire Hospitals NHS Trust, Burnley, UK

###### **Correspondence:** John Tomlinson

In 2014 a Multidisciplinary Special Interest Group in Greater Manchester, Lancashire and South Cumbria was convened with a remit to improve care for women who present with intra-uterine fetal death and experience stillbirth. A clinical guideline was synthesised from available evidence and national clinical guidance. To aid implementation an integrated care pathway was also introduced to facilitate delivering optimal care. The first version of these documents was released in December 2014 and updated in February 2016; this was then used in the 13 hospitals in the geographical area.

An audit of care was undertaken prior to implementation with two further audit data collections, the first was performed 1 year after introduction of the documents. The second audit was performed a year after the guideline was updated. Data were collected using a standardised proforma.

A total of 87 cases were included in this audit, which included 29 cases from the baseline, 27 in the first year 1 and 31 at the second time point (Table 8). This audit demonstrated that use of the integrated care pathway improved the care of women who present with intra-uterine fetal death and stillbirth. There was an increase in the number of women being given a patient information leaflet. A greater proportion of women had a recommended method of induction of labour (e.g. dose and timing of misoprostol) and more likely to receive appropriate opiate analgesia when in labour. Staff gave favourable feedback about using the integrated care pathway, with many suggesting that it aided care.

Use of this integrated care pathway improves care for women who present with fetal death in utero and experience stillbirth. Ongoing development is required to ensure that improvements to care and feedback from staff suing the tools are incorporated.

**Table 8 (abstract P44).** See text for description

	2014	Post implementation Audit 1	Post implementation Audit 2
Number	29	27	31
Presentation			
Antenatal	23	17	23
Termination for fetal abnormality	1	4	2
Intrapartum	3	5	6
Unclassifiable	1	1	
Urgent Management Required	4/29 14%	11/27 41%	7/31 24%
Patient Information Leaflet Given - if urgent management not needed	17%	37%	65%
Expectant management offered - if urgent management not needed	24%	27%	32%
If induction of labour performed - National Guidance followed	0%	73%	70%
Analgesia in labour – ratio of use of diamorphine compared to pethidine	67%	75%	84%

### P45 Whose fault is it? Maternal guilt and blame 15 months after perinatal death

#### Katherine Gold^1^, Ananda Sen^3^, Irving Leon^3^

##### ^1^Department of Family Medicine, Department of Obstetrics & Gynecology, University of Michigan, Ann Arbor, MI, USA; ^2^Department of Family Medicine, Department of Biostatistics, University of Michigan, Ann Arbor, MI, USA; ^3^Department of Obstetrics & Gynecology, University of Michigan, Ann Arbor, MI, USA

###### **Correspondence:** Katherine Gold

Parents who experience stillbirth or infant death often struggle with postpartum guilt. This may be an adaptive response or can become chronic and maladaptive.

We conducted a longitudinal, three-wave mail survey over two years of bereaved mothers in Michigan (United States) who experienced perinatal death. At 15 months after loss, survey domains included questions on demographics, depression, guilt, and blame. We also analyzed whether data available at 9 months predicted guilt at 15 months. Self-report information was linked with data from an earlier survey and with State of Michigan birth and death certificates.

311 (42%) mothers responded. Most reported persistent guilt or self-blame. In multivariable analysis, higher education level (OR 2.55), depression (OR: 7.60), and presence of a maternal medical risk factor (OR 4.17) predicted greater guilt. Nearly half of women blamed their medical team for the loss, about a fifth reported feeling blamed by others. Both of these attributions were significantly associated with greater guilt in multivariable regression. Thirteen percent of women stated someone had told them to their face that the death was their fault. No other socioeconomic factors besides education were significant predictors.

The majority of mothers report persistent guilt after perinatal loss. Depression at either 9 or 15 months, higher education, and maternal medical risk factors were the strongest predictors of guilt. Attribution of blame served no protective function for mothers. While many women do not spontaneously reveal their thoughts of guilt, the high correlation between guilt and depression suggest that early screening for depression may be one option to identify women who may also be struggling with guilt or self-blame for their loss.

### P46 When there are no words: loss, meaning making and the arts

#### Louise Foott^1,2^

##### ^1^CIT Crawford College of Art & Design, Cork, Ireland; ^2^University College Cork, Cork, Ireland

Stillbirth is recognized as a traumatic loss, but there remains considerable silence around it. Current research into ‘grief work’ challenges the stages model of grieving, advocating for an approach that supports the grieving to create meaning within their experience of loss. At a time when words feel inadequate, the arts can enable meaning making through a range of different approaches.

Drawing on her own experience following the still birth of her youngest child, Laura, this auto ethnographic presentation will explore the role of a range of arts approaches within one family’s grieving process. Laura died as the author was conducting research into the role of the arts within reflective learning. Several years later this author now aims to look at her family’s own journey with grief through the lens of her research interests, exploring the important support provided by the arts within the family’s search for meaning. The author discussed her auto ethnographic research with her now teenage family and they gave their informed consent to be included.

**Fig. 4 (abstract P46).**
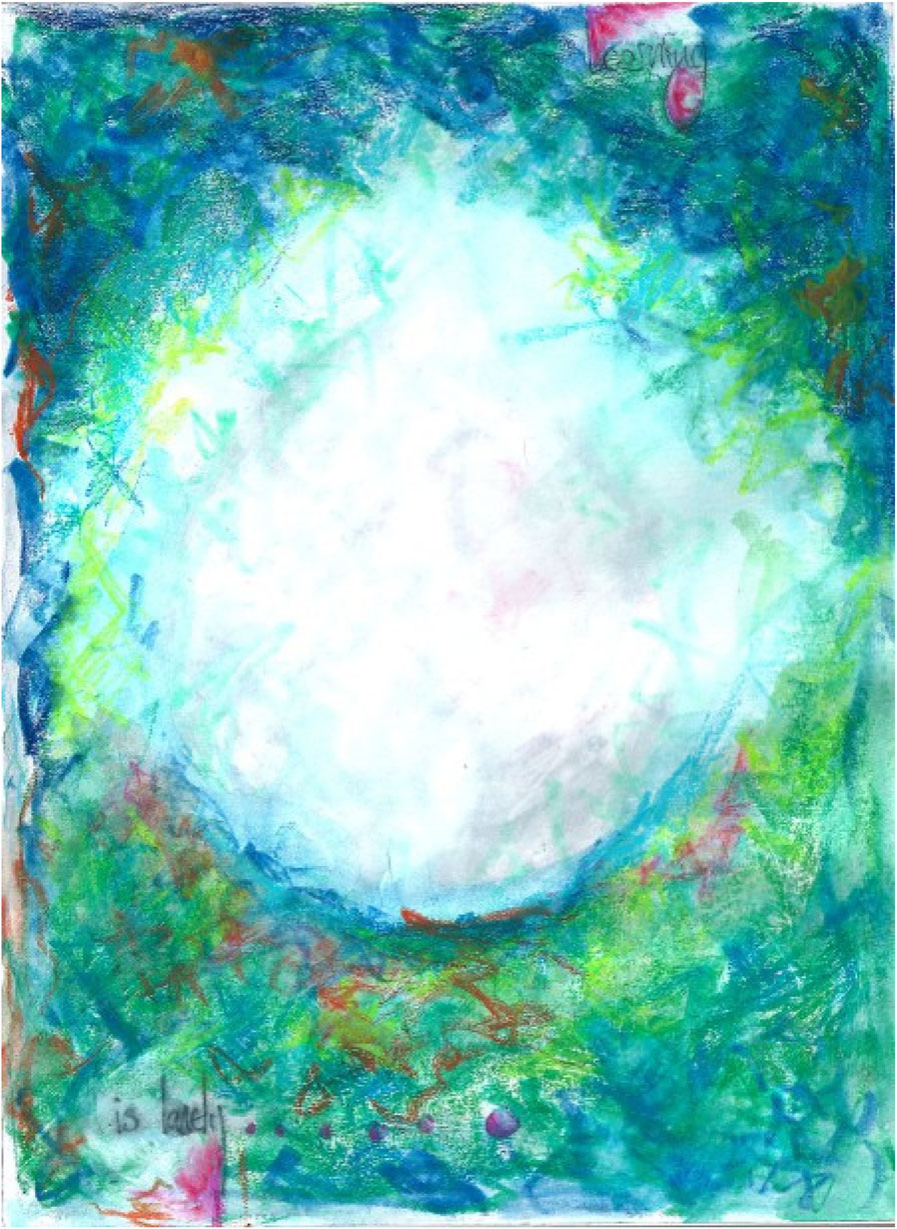
See text for description

Key aspects of this family’s experience of grieving the still birth of Laura will be documented through a multi-media presentation. Creative writing, poetry, image-making and music will give voice to the silence around particular characteristics of the trauma of stillbirth – relational connection to a child never met, meaning making when nothing makes sense and the importance of acknowledging the senses in grieving and memory making. (Fig. 4) As they journeyed with their loss, the arts supported this family to incorporate “Laura missing” in their lives.

Much of the current discourse on death and bereavement advocates for an approach that enables the grieving to ‘craft’ meaningful experiences as they navigate their way through loss. When the weight of grief exposed the limits of words, the arts offered this family other languages to connect and make sense of their loss.

### P47 Understanding the associations and significance of fetal movements in overweight or obese pregnant women: a systematic review

#### Billie F. Bradford^1^, John M. D. Thompson^1,2^, Alexander E. P. Heazell^3,4^, Lesley M. E. McCowan^1^, Chris J. D. McKinlay^2,5^

##### ^1^Department of Obstetrics and Gynaecology, Faculty of Medical and Health Sciences, University Of Auckland, Auckland, New Zealand; ^2^Department of Paediatrics: Child and Youth Health, Faculty of Medical and Health Sciences, University of Auckland, Auckland, New Zealand; ^3^Maternal and Fetal Health Research Centre, School of Medical Siences, Faculty of Biological, Medical and Human Sciences, University of Manchester, Manchester, UK; ^4^St. Mary’s Hospital, Central Manchester University Hospitals NHS Foundation Trust, Manchester Academic Health Science Centre, Manchester, UK; ^5^Liggins Institute, University of Auckland, Auckland, New Zealand

###### **Correspondence:** Billie F. Bradford, Alexander E. P. Heazell

Presentation with decreased fetal movement (DFM) is associated with fetal growth restriction (FGR) and stillbirth. DFM may be more frequent amongst overweight or obese mothers. Perception of fetal movements is widely believed to be impaired in heavier mothers due to increased abdominal fat, but this association has not been systematically assessed.

Objectives

To determine the significance and associations of fetal movements in women of increased body size.

This systematic review was conducted in accordance with the PRISMA statement and the protocol was registered (PROSPERO CRD42016046352). Major databases were searched from inception to November 2016, using a pre-defined search strategy. Studies of any design that compared fetal movements in women of increased and normal body size were included. Two authors independently extracted data and assessed quality.

22 publications from 18 observational studies were included and data were extracted from 10 studies. Increased maternal body size was not associated with altered perception of fetal movement (4 studies, 95 women, very low quality evidence), but was associated with increased presentation for DFM (2 cohort studies, 20,588 women, OR 1.56, 95% CI 1.27-1.92: 3 case-control studies, 3,445 women, OR 1.32, 95% CI 1.12-1.54; low quality evidence).

Amongst women with DFM, increased maternal body size was associated with increased risk of stillbirth and FGR (1 study, 2,168 women, very low quality evidence).

This systematic review identified limited evidence that women with increased body size are more likely to present with DFM but do not have altered perception of fetal movements. In women with DFM, increased body size is associated with worse pregnancy outcome, including stillbirth. Concerns about DFM in larger women may have greater clinical significance and should not be downgraded due to assumptions about impaired perception of fetal movements, though further confirmatory studies are warranted.

### P48 Perinatal mortality: mourning three - four years after the loss and the need for counseling

#### Janine Van Veen - Doornenbal, Judith Derks, E. van Leeuwen, I. M. de Graaf

##### Academic Medical Centre, Amsterdam, The Netherlands

###### **Correspondence:** Janine Van Veen - Doornenbal

Grief gradually decreases in time after perinatal death. Not much is known about long term grieving after perinatal death. We studied the magnitude of grief after 3-4 years.

We randomly selected cases of perinatal death between 2013-2014 in the Academic Medical Centre (Amsterdam). Women and partners were included if pregnancy was longer than 22 weeks and death occurred within 28 days after birth. We included 44 mothers with 30 partners (all male), 53% filled out the questionnaires. They separately completed the Perinatal Grief Scale (PGS-33), the Dutch Grief Scale and the Hospital Anxiety and Depression Scale (HADS). A selection of patients and their partners underwent a structured interview to evaluate the psychological counselling that was given at that time.

The results show, first, that part of the population experiences high levels of perinatal grief after 3-4 years. 11 of 39 subjects scored high (>7) on HADS-anxiety and 5 of 39 persons scored high (>7) on HADS-depression and both grief scales. Second, fathers had a significant positive relation between the period the baby lived and their PGS-score, while this relation is not significant for mothers. As a result, couples with stillbirth differ in the amount of grief. This difference disappears when the baby lives longer. Last, from the structured interviews we learned that a standard protocol for aftercare is of great importance, and that the guidance should be adjusted to the needs of the parents.

After 3-4 years, perinatal grief is still present. Those with high scores on anxiety and depression show symptoms of traumatic grief. We propose that filling out the PGS-33 and HADS should be included in standard protocols after perinatal death to identify patients at risk. The participants expressed that psychological aid after perinatal mortality is important and should be incorporated into standard care.

### P49 The experience of Australian midwives caring for women with undiagnosed vasa praevia during labour: a qualitative study

#### Nasrin Z. Javid^1^, Jon A. Hyett^2^, Caroline SE. Homer^1^

##### ^1^Centre for Midwifery, Child and Family Health, University Of Technology Sydney, Sydney, Australia; ^2^Royal Prince Alfred Women and Babies, University of Sydney, Sydney, Australia

###### **Correspondence:** Nasrin Z. Javid

Vasa praevia is a rare pregnancy complication that can cause stillbirth and early neonatal death if it is not diagnosed antenatally. Caring for women with undiagnosed vasa praevia during labour and birth is challenging and often traumatic. There is no qualitative research that examines the experiences of midwives. The aim of this study was to explore the experience of Australian midwives caring for women with vasa praevia during labour.

A qualitative descriptive study was undertaken with Australian midwives who had cared for at least one woman with vasa praevia during 2010-2016. Recruitment was mainly achieved through Australian College of Midwives and snowball sampling recruited additional midwives. Semi-structured in-depth interviews were conducted over the phone, digitally recorded and transcribed. Interviews lasted 40-140 minutes. Data were analysed using thematic analysis.

Twenty midwives were interviewed from public and private hospitals across Australia; 13 of these were involved in the care of the women with undiagnosed vasa praevia who experienced a neonatal death (n = 6) or near miss (n = 7). The over-arching theme was ‘A devastating and dreadful experience’ and included two themes of feeling the personal impact and addressing professional processes. The personal impact included ‘feeling scared’, ‘feeling shocked’, ‘feeling guilty’, and these ‘took their toll’. The professional processes included ‘working in organised chaos’, ‘challenges with intrapartum diagnosis’, ‘feeling for the parents’, ‘feeling communication was hard’, and ‘doing our best as we did not know’.

Our findings demonstrate the emotional impact experienced by midwives caring for women with vasa praevia. Caring for women who have undiagnosed vasa praevia during labour poses unique challenges for the midwives both at personal and professional level, as they witness a healthy baby rapidly becoming sick and potentially dying.

Ethical approval for the study was granted by the Human Research Ethics Committee of University of Technology Sydney on 9 March 2016 (HREC ETH15-0137). Written informed consent was obtained by all study participants.

### P50 A proposed method for increasing the diagnostic yield of placental tissue and identification and measurement of placental chronic villitis

#### Louise Sutton^1^, Lisa Hogan^1^, Patrycja Abrmczuk^1^, Des Butler^1^, Sarah Clancy^1^, John Gray^1^, Peter Kelehan^1^

##### Bon Secours Hospital Limerick at Barringtons, Georges Quay, Limerick, Ireland

###### **Correspondence:** Louise Sutton; Peter Kelehan

Chronic Villitis of unknown aetiology is an inflammatory disease of the placenta, which is focal and local in its distribution. Identification is dependent on sampling, and mild disease is recognized and quantified, depending on the number of tissue blocks taken. Examination of four sections of placental parenchyma should identify more disease than two.

We set out to show, in our prospective study, sampling two blocks of placental parenchyma, whether taking a further two blocks increased yield and changed grade. We further suggest a novel method that may also be used to increase yield in a retrospective study and a method that more accurately measures extent of disease.

When initial diagnosis of chronic villitis was made on H/E sections of two tissue blocks, two further random blocks were sampled from the placenta prior to disposal and extra sections were taken for Immunohistochemistry and special stains. The four blocks were then taken back to molten paraffin wax, flipped 180 degrees, recut and extra sections taken. This yielded eight levels of tissue from each placenta for the study. Slides were scanned using an Ion Film 2 SD Pro 9 Slide Scanner. Images were printed onto A4 sheets, the total area of villous parenchyma and immunohistochemically stained area were measured (cm^2^), subtracted, and a percentage calculated.

**Fig. 5 (abstract P50).**
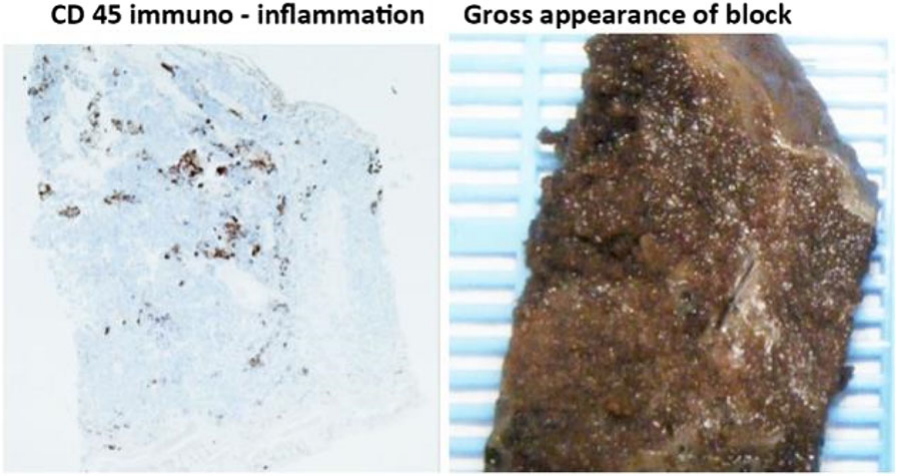
Chronic villitis of placenta

Twenty-one singleton and eleven twin placentas with chronic villitis were examined in a nine-month prospective study period. Direct comparison of the histological appearance and pattern of the two faces of each tissue block confirmed the structural heterogeneity of the placenta and also showed variation of density of inflammation best appreciated by immunohistology (Fig. 5). Statistical analysis showed no significant difference. Staining for CD45 and CD 3 produced the best results.

Chronic villitis is a potentially recurrent placental disease with poor outcome. This methodology can increase yield and accurate grading.

Ethical approval for the study was granted by Barringtons Hospital Ethics committee and the University of Ulster, Colraine.

### P51 “Better safe than sorry”- Reasons for consulting care due to decreased fetal movements

#### Anders Linde^1,2^, Ingela Rådestad^2^, Karin Pettersson^1^, Linn Hagelberg^2^, Susanne Georgsson^1,2^

##### ^1^Karolinska Institutet, Stockholm, Sweden; ^2^Sophiahemmet University, Stockholm, Sweden

###### **Correspondence:** Anders Linde

Experience of reduced fetal movements is a common reason for consulting health care in late pregnancy. There is an association between reduced fetal movements and stillbirth. We aimed to explore why women decide to consult health care due to reduced fetal movements at a specific point in time and investigate reasons for delaying a consultation.

A questionnaire was distributed at all birth clinics in Stockholm during 2014, to women seeking care due to reduced fetal movements. In total, 3555 questionnaires were collected, 960 were included in this study. The open-ended question; “Why, specifically, do you come to the clinic today?” was analysed using content analysis as well as the complementary question “Are there any reasons why you did not come to the clinic earlier?”

Five categories were revealed: Reaching dead line 348 (43%), Receiving advice from health care professionals 280 (35%), Undergoing unmanageable worry 196 (24%), Contributing external factors 123 (15%) and Not wanting to jeopardize the health of the baby 18 (2%). Many women stated that they decided to consult care when some time with reduced fetal movements had passed. The most common reason for not consulting care earlier was that it was a new experience. Some women stated that they did not want to feel that they were annoying, or be perceived as excessively worried. Not wanting to burden health care unnecessarily was a reason for prehospital delay.

Worry about the baby is the crucial reason for consulting care as well as the time which has passed since the women first experienced decreased fetal movements.

Ethical approval for the study was granted by Stockholm Research Ethics Committee of Sweden (Reference; Dnr 2013/1077-31/3). Written informed consent was obtained by all study participants.

### P52 A pilot study exploring stillbirth stigma experiences in Australia and adapting and validating a stigma scale

#### Danielle Pollock, Jane Warland, Tahereh Ziaian, Elissa Pearson, Megan Cooper

##### University Of South Australia, Adelaide, Australia

###### **Correspondence:** Danielle Pollock

The silence surrounding stillbirth has led to the wide belief that there is a stigma associated with stillbirth. Research in other fields where stigma is known to be prevalent such as HIV/Aids, suggests that stigmatisation often leads to a reduction in help- seeking behaviours, increased isolation and limited social support. Each of the consequences of stigma are also seen in stillbirth literature. Despite this, research regarding stigma stillbirth is limited, and therefore often assumed. This assumption is demonstrated by the WHO, and 2011 and 2016 Lancet series, all calling for action to reduce stigma in stillbirth. However, the extent, type and experiences of stillbirth stigma have not yet been established or explored.

A stillbirth stigma scale is being created as part of a larger mixed methods study. When developing the scale a survey was included asking parents of stillborn babies to describe their experiences during pregnancy, labour, and afterwards. The survey was completed by n = 98 Australian bereaved parents with a further n = 36 who conducted test-retest for scale validation.

Survey results suggest that antenatal education on stillbirth and fetal movements was poor, with less than 12% of bereaved parents saying they were given enough information to detect possible signs of stillbirth. Furthermore, qualitative analysis showed that after care was minimal, with many bereaved parents stating that they sought psychological help themselves. However, there were also positive stories of supportive hospital care and memory creation practices. Further data analysis has yet to be conducted, but will be presented at the conference.

While some improvements in care provision around the time of stillbirth have been made, further antenatal education needs to occur. It is particularly important for maternity care providers to give information to all women regarding stillbirth during pregnancy especially the importance of awareness of fetal movements.

Ethical approval for the study was granted by UniSA HREC Research Ethics Committee of 0000036071. Consent was obtained by all study participants before the completion of the online survey.

### P53 Umbilical cord thrombosis: association with stillbirth and opportunities for stillbirth prevention

#### Jessica Liauw, Julie Robertson, Christof Senger, Jennifer Hutcheon

##### University of British Columbia, Vancouver, Canada

###### **Correspondence:** Jessica Liauw

Umbilical cord thrombosis has been associated with growth restriction, fetal distress, and perinatal death. However, these associations are largely based on pathology case reports and high-risk cohorts, which are vulnerable to selection bias. We aimed to determine the associations between umbilical cord thrombosis and adverse perinatal outcomes among all deliveries in one large center. Since prenatal diagnosis of umbilical cord thrombosis has been reported, we also aimed to describe opportunities for stillbirth prevention.

Our study population included all non-anomalous births ≥20 weeks’ gestation at the Royal Victoria Hospital in Montreal, Canada (2001-2009). All placentas underwent routine pathological examination. Umbilical cord thrombosis was correlated with obstetric, neonatal, and pathology characteristics. We calculated odds ratios for the effect of cord thrombosis on stillbirth, small for gestational age birthweight (SGA), and neonatal death, adjusting for gestational age and maternal comorbidities. We determined the proportion of stillbirths that had umbilical cord thrombosis and did not have antenatal doppler studies, which were used as a surrogate for antenatal monitoring.

Among 27,940 deliveries, the incidence of umbilical cord thrombosis was 1 per 1000 (95%CI 0.6 to 1.4 per 1000). These were present in 41% of stillbirths, 11% of neonatal deaths, and 11% of those with SGA. Umbilical cord thrombosis was significantly associated with stillbirth (adjusted OR 166.4, 95%CI 62.3 to 444.4), and there was a trend towards increased odds of neonatal death (adjusted OR 5.2, 95% CI 1.0 to 28.6). Umbilical cord thrombosis was not associated with SGA (adjusted OR of 1.0, 95% CI 0.3 to 3.5). Among stillbirths with no antenatal doppler studies, 16% had an umbilical cord thrombosis.

Umbilical cord thrombosis is strongly associated with stillbirth, controlling for selection bias and possible confounders. It may be possible to prevent a proportion of stillbirths via antenatal identification of umbilical cord thrombosis.

Ethical approval for the study was granted by the University of British Columbia/Children’s and Women’s Health Centre of British Columbia Research Ethics Board (Reference; CW14-0341/H14-02809).

### P54 How hard is it to grow a rainbow? The emotional and mental health complications during a pregnancy after loss

#### Suzanne Maguire

##### Sands NI, Portadown, Northern Ireland

This research will highlight the serious impact the death of a baby has on a woman’s mental and emotional health in a subsequent pregnancy. It emphasises a woman and her partner’s increased risk of experiencing mental health problems and the possible difficulties with their ability to bond with the baby. It asks how antenatal care can better support people through this experience.

Research has been undertaken through the mediums of online forums and online surveys as this reaches a wider audience and is more accessible. Published literature is also utilised.

The death of a baby significantly affects a mother’s mental and emotional wellbeing in a subsequent pregnancy. The results highlighted that a woman’s experience of pregnancy and her ability to believe in the viability of the pregnancy, even when it is not complicated, is hindered. The mother, and indeed her wider social network, may not bond with the baby as a type of protection should loss happen again.

When a woman losses a baby either through stillbirth, neonatal death, or miscarriage, her ability to view the world as safe and straightforward diminishes. Pregnancy, an act which is supposedly one of the most natural things she can do, becomes an area of heightened anxiety, depression, emotional turmoil, and unease. A subsequent pregnancy was not an enjoyable experience for almost all women within this study. The poor mental health of the mother may be alleviated somewhat through the care received from medical staff. However, most mothers were offered no extra appointments or emotional support throughout the subsequent pregnancy. Care and support needs to be available so the mother can bond with her unborn child, therefore, paving the way for a stable and loving relationship once the baby is born.

### P55 Awareness of reduced foetal movements: a one-way street?

#### Suzanne Maguire

##### Sands NI, Enniskillen, Northern Ireland

This research focuses on the phenomenon of reduced foetal movements and asks whether both sides - the mother and the medical professionals - view the episode with equal significance. Recent campaigns aimed at mothers monitoring their babies movements have been successful for raising awareness. However, has this raised awareness infiltrated the medical profession?

Online forums and surveys have been used to establish how mothers felt their concerns about reduced foetal movements were viewed. Published literature has also been utilised.

When a woman presents with reduced foetal movements, she is still sometimes met with resistance. In a crowded maternity ward, and with pressures on staffing numbers, some mothers have felt that they were wasting time with their concerns. A significant number of women in this study were discharged without their concerns being alleviated and some felt that they were not treated kindly. A few went on to experience the death of their baby.

Research has shown that a change to a baby’s movements is one of three risk factors to indicate an impending stillbirth or neonatal death. Successful campaigns have led to women being aware of counting the kicks, watching for movement patterns, and the importance of ringing a health professional if they are worried about their baby’s movements. However, some women are still being told that a reduction in movements is ‘normal’ and once a heartbeat has been detected they are sent home and told ‘not to worry’. This is a false misconception, leads to confusion, and places unborn babies, and sometimes their mothers, in serious harm. This paper argues that medical professionals must place more importance on reduced foetal movements. However, with resources already stretched, it questions how this can be done.

### P56 Fetal movement awareness: reducing stillbirth in Scotland

#### Cheryl Clark, Bernie McCulloch, Angela Cunningham, Clare Willocks

##### Healthcare Improvement Scotland, Edinburgh, Scotland

###### Correspondence: Bernie McCulloch

**Fig. 6 (abstract P56).**
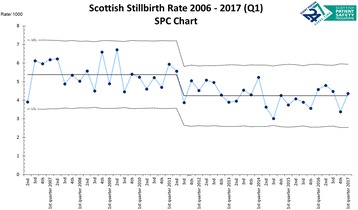
Scottish Stillbirth rate 2006-2016

In Scotland, 274 babies were stillborn in 2012 (a rate of 4.7 per 1000 births; see Fig. 6). In 2013, Healthcare Improvement Scotland launched the Maternity and Children Quality Improvement Collaborative (MCQIC), as part of the Scottish Patient Safety Programme. A key aim of MCQIC is to reduce the Scottish rate of stillbirth by 35% by 2019. The cause of stillbirth is complex but it is recognised that the need to monitor fetal movement throughout pregnancy is an important health message for women. Best practice suggests fetal movement should be discussed between 18–24 weeks gestation, but baseline data for 12 sites between March to August 2014 showed this was not consistently achieved.

Local test teams in every health board in Scotland were given tools to support measurement. These included a clear operational definition of fetal movement discussion and a sampling strategy and tools to display data in time sequence. Using the Model for Improvement, maternity teams tested ideas on a small scale and collected data to confirm if the changes resulted in an improvement. Change ideas included patient information leaflets and pocket cards.

National aggregated data from 10 of 17 boards which have reported consistently from March 2014 to December 2016 show discussion of fetal movement improved by 21%

In 2015, national outcome data showed 211 babies were stillborn, a 19.5% reduction in the rate of stillbirth compared to 2012. Although no one factor or programme can be attributed to this decline and the rate has slightly increased in 2016, it is encouraging progress towards reducing the stillbirth rate in Scotland.

### P57 Children’s Nurses’ experiences in delivering bereavement care to children and families with life limiting conditions in the Irish context

#### Stacey Power^1^, Marcella Kelly-Horrigan^2^

##### ^1^University College Dublin, Stilorgan Rd, Belfield, Ireland; ^2^NUI Galway, University Rd, Galway, Ireland

###### **Correspondence:** Stacey Power

Healthcare providers influence the experiences of families during end of life care and death of a child*. Nurses are best placed to provide bereavement support as they have opportunities to build therapeutic relationships through closely and frequently caring for the child and family. This relationship is essential within the delivery of bereavement care. However, there is a dearth of information on nurses’ emic perspective and experiences within this area.

The aim of this study was to gain a deeper understanding of the experiences of children’s nurses’ (RCN) in delivering bereavement care to children and their families with life limiting conditions, and what meaning they ascribe to their experience. In addition, the aim was to explore what impact provision of bereavement care had on RCN’s as service providers, and what their needs were in the provision of effective, supportive, quality driven bereavement care to this population.

Using a phenomenological design guided by Heideggerian approach underpinned by Ricoeur’s analytical framework, seven semi-structured interviews were conducted with RCN’s with experience of delivering children’s palliative care and bereavement care in Ireland. Interviews were taped and transcribed verbatim.

Three themes were identified; ‘being communicative and collaborative’, ‘being challenged’ and ‘being familiar’. These themes encompassed nurses’ experiences with both families and healthcare professionals, highlighting the benefits for RCN involvement in the delivery of bereavement care to promote overall best outcomes.

The findings support the role of RCN’s in the delivery of bereavement care to children and families with life limiting conditions. It highlights the need for RCN’s to be educated, up-skilled, supported, and included within the interdisciplinary team to deliver bereavement care.

Ethical approval for the study was granted by LauraLynn Research Ethics Committee (2016). Written informed consent was obtained by all study participants.

*denotes age from newborn to eighteen years

### P58 Still aware lets share

#### Linda Doran

##### Tara’s Tiny Footprints, Castledermot, Ireland

As a parent who suffered the stillbirth of our daughter in 2006 at full term due to an umbilical cord accident, I wish to raise awareness and educate people that stillbirths happen in Ireland and to remove the taboo and stigma surrounding stillbirth in society. In 2014 there were 164 stillbirths in Ireland a rate of 2.4% per live births, and an estimated 2.6 million stillbirths occur every year worldwide (per WHO).

I aim to introduce an information leaflet that can be displayed in antenatal waiting rooms, GP’s surgeries and public information areas that educates mothers to trust their maternal instincts and seek professional reassurance if they are anyway concerned. Not all stillbirths can be prevented however there are things you can do to reduce your risk which could also be outlined in the leaflet. These include, attending all antenatal appointments, good health and nutrition before and during pregnancy and monitoring and being aware of your babies movements throughout the day.

The information leaflets will raise the subject of stillbirth and introduce the dialogue and awareness into the conversation with medical staff, families and society. It will educate parents on how to reduce the risk of stillbirth in a safe non alarming way.

It is assumed that stillbirths only happen in high risk pregnancies which is just not true. A babies only direct link to the outside world is through its mother. We need to empower women and give them the confidence to trust their instincts when something feels wrong during their pregnancy and to act on it immediately. In 2014, the World Health Assembly endorsed a target of a rate of 1.2% or fewer stillbirths in every country by 2030. Let’s share and be still aware.

### P59 The Irish childhood bereavement care pyramid – planning for siblings bereaved through stillbirth

#### Orla Keegan^1,2^, Anne Marie Jones^3,2^, Celine Deane^4,2^

##### ^1^Irish Hospice Foundation, Dublin, Ireland; ^2^Irish Childhood Bereavement Network, Dublin, Ireland; ^3^Temple Street Children’s University Hospital, Dublin, Ireland; ^4^Beaumont Hospital, Dublin, Ireland

###### **Correspondence:** Orla Keegan

Understanding of children’s bereavement needs in general and bereaved siblings in particular are traditionally under-researched areas. The specific needs of siblings bereaved through stillbirth are even less well understood. This presentation aims to introduce the Irish Childhood Bereavement Care Pyramid (ICBCP) and to discuss its potential for understanding bereavement experiences and the service implications for siblings bereaved through stillbirth.

Development of ICBCP: Literature review, consultation, integration of feedback, dissemination of findings

Additional literature review (siblings; bereaved; stillbirth)

**Fig. 7 (abstract P59).**
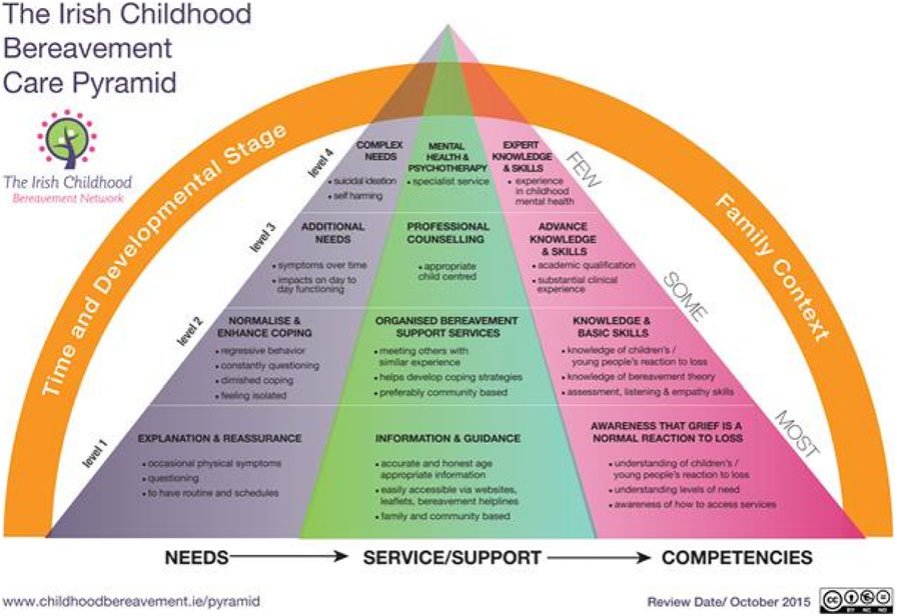
The Irish Childhood Bereavement Pyramid

The family and the passage of time are crucial contexts for support. All bereaved children need to be met with empathy and understanding; adults have a responsibility to inform themselves and respond. Smaller numbers of children develop more complex needs and require formal supports – peer-based, voluntary groups or professional intervention. The ICBN Pyramid provides a four level framework to indicate responses to children’s bereavement needs. (Fig. 7)

There were 330 stillbirths in Ireland in 2014, (circa 300 annually). The number of siblings bereaved in this way is estimated conservatively as upward of 3,000 over ten years. A recent population-based longitudinal case-control study identified bereaved siblings (any cause) under 13yrs at risk of developing a mental disorder, and most likely to develop depression. Specific to the psychological impact of stillbirth a systematic review identified long-term mental health impacts & hyper-vigilant parenting as characterizing many bereaved children’s lives. The 2016 Irish National Standards for Bereavement Care following Pregnancy Loss identify siblings as part of bereavement care concern; implementation guidance on the most effective means to achieve this is yet to be developed.

There are gaps in literature and the extent to which family, community, health services & specialist services meet siblings’ needs is unestablished. The pyramid of bereavement care situates bereaved siblings and provides an initial guide for support protocols.

### P60 Women’s awareness of stillbirth and reaction to messaging around stillbirth risk

#### Janet Scott, Laura J. Price

##### Sands (the stillbirth and neonatal death charity), London, UK

###### **Correspondence:** Janet Scott; Laura J. Price

Women’s awareness of stillbirth and reactions to stillbirth messaging were explored.

Focus group sessions were conducted involving 40 women with no experience of stillbirth (Table 9). Groups were shown four sample A3 posters and a leaflet. Discussions were independently facilitated, with a note taker behind one-way glass.

Awareness of stillbirth was limited. Although most women considered they ‘sort of knew’, none could define stillbirth accurately. Pregnant and previously pregnant women recalled being told about alcohol, smoking and healthy eating, and receiving information on miscarriage, cot death and Down’s syndrome. Some second- and third-time mothers felt they knew enough and were less likely to read information. One younger woman reported getting all her information from YouTube.

Women were surprised by stillbirth statistics. The incidence of stillbirth vs cot death had the most impact, but some women said it simply made them feel less concerned about SIDS. In general, women found statistics scary if they were not linked with advice about risk reduction. Women were surprised that flu was a risk factor, and many thought vaccination would be harmful. There was mixed reaction to information on drinking, with the suggestion that ‘overstating’ the risk could damage trust in other messages. Women were familiar with the smoking messages, though none realised there was a link with stillbirth.

Women agreed that they wanted information on modifiable risk factors only. Women felt information on non-modifiable risk factors such as ethnic group should be ‘known’ by health professionals.

Women across the UK were consistent in their knowledge and preferences. Messaging around stillbirth needs to be subtle, focusing on how to reduce risk and have a safer pregnancy. Imagery needs to reflect positive messaging and not imply loss or bereavement. Statistics should be used with care and not in isolation.

The focus group research presented in this abstract was carried out by The Focus Group, on behalf of Sands, the stillbirth and neonatal death charity. All participants gave written consent to participate

**Table 9 (abstract P60).** See text for description

**Description**	**Location**	**Age range (years)**	BME* **(%)**	***n***
Primigravidae	London	23-35	33	6
Secundi- and primigravidae	Manchester	33-45	33	3
Secundi- and plurigravidae	Bournemouth	32-40	0	7
Secundigravidae	Belfast	27-37	0	7
Secundigravidae	Glasgow	29-33	0	3
Secundi- and plurigravidae	Cardiff	25-28	0	6
Mothers with children, not currently pregnant but considering more children	London	36-43	12.5	8

*BME, black and minority ethnic

### P61 Association of stillbirth rate with community access to skilled birth attendance in rural Bangladesh

#### Louise Tina Day^1^, Johan P Velema^1^, Steven Withington^1^, Stacy L Saha^1^, Shafiul Alam^1^, Nazimul Hossain^1^, Shirajum Munira^1^, Khaleda Jesmin^1^, Swapan Pahan^1^, Kristine Prenger^1^, Dan Hruschka^2^

##### ^1^Lamb Integrated Rural Health & Development, Parbatipur, Bangladesh; ^2^Arizona State University, Tempe, US

###### **Correspondence:** Louise Tina Day

Reducing barriers to quality skilled birth attendance that reduces perinatal mortality (PNM) is a continuing challenge. In the high stillbirth setting of Bangladesh, Medically Trained Provider (MTP) deliver only 42% of births. LAMB Integrated Rural Health and Development has been working to reduce PNM with Emergency Obstetric and Newborn Care (EmONC) referral linkages from home to hospital. In LAMB community area, 2-3 Community Skilled Birth Attendants (CSBA), who are designated as MTP, are posted to a 2-bed Safe Delivery Unit (SDU) which functions 24 hours a day at Obstetric First Aid EmONC level. One SDU covers a median community population of 28,500 and approximately 500 pregnancies/year.

To examine how establishing and strengthening community services are associated with changes in stillbirth rates, we analyse the occurrence of stillbirths among 39,459 deliveries (2011-2014) in a rural Bangladesh community served by 19 SDUs. Stillbirth rates were also analysed against key determinants using multivariate analysis.

Of all mothers who delivered, 70% had 3 antenatal visits and 65% delivered with MTPs (national average 50%) with 17% delivered by SBA in the SDU-FWC (national average CSBA deliveries 0.4%). Stillbirth rates (per 1000 births) varied considerably across place of delivery (road deliveries 45.5, home 21.6, SDU-FWC by SBA 9.6, overall 22.2). After adjusting for covariates, the risk of stillbirth was lower by 33% (adjusted OR 0.67) for SDU-FWC delivery compared to homebirth.

National uptake of CSBA as delivery attendants remains low in Bangladesh, but placing them in the enabling environment of a local SDU-FWC facility was associated with increased local usage of their skilled care. This was associated with a stillbirth reduction by one third compared to unskilled deliveries at home.

Ethical approval for the study was granted by LAMB Research Ethics Committee. (Reference; 6/REC/17)

### P62 Sustaining perinatal audit in the high stillbirth rate setting – a 20 year journey in rural Bangladesh

#### Louise Tina Day, Felicity Mussell, Hafiza Khatun, Renate Verbiest, Lipi Biswas, Rekha Folia, Robyn Turner, Ruth Yvonne Lennox, Kristine Prenger, Christine Edwards, Beatrice Ambauen-Berger, Stacy L. Saha

##### Lamb Integrated Rural Health & Development, Parbatipur, Bangladesh

###### **Correspondence:** Louise Tina Day

Bangladesh has a high population stillbirth (SB) rate of 36/1000 total births. LAMB Hospital, a 150-bed hospital, serves northwest rural Bangladesh as part of LAMB Integrated Rural Health and Development NGO providing Comprehensive Emergency Obstetric and Neonatal Care for > 3000 deliveries/year.

We describe challenges faced and overcome during 20 years implementation of Perinatal and Maternal Death Audit (PNMDA) in our busy, resource-limited setting with high staff turnover and constant competing demands. Perinatal mortality rates were measured using Perinatal Problem Identification Programme (PPIP) software.

The process now includes a number of key components that facilitate sustainability: (1) Efficient data collection methods for quantitative analysis; (2) Inclusive regular multidisciplinary meetings including near miss deaths for positive feedback; (3) Implementing integrated patient and staff-friendly solutions to issues identified; (4) Caring for Health Care Providers (HCP) including creative task shifting; (5) Emotional/Spiritual care for families and HCP; (6) Using computerised data management software: PPIP and locally customised FISH (Flow Information System Hospital). The process has also created new opportunities, including (1) Involvement nationally in dissemination and training; (2) Sharing data for International Stillbirth Comparison; (3) Developing next generation of PNMDA champions.

Quantitative analysis of more than 50,000 babies born > 1000g during the years 2001 to 2017 has shown stillbirth rates falling from 55 to 30/1000 total births. The SB: Neonatal Death ratio was 1.6 : 1.0 Patient associated “Avoidable Factors” were identified in 85% of Stillbirths.

PNMDA is a valuable process in the high SB setting and can be sustained with creativity and motivation. Maintaining a holistic attitude alongside quantitative analysis of mortality rates and focusing on “caring for the carers” for the Health Care Providers at high risk of emotional fatigue can make an important contribution to the provision of respectful maternity services for families.

Ethical approval for the study was granted by LAMB Research Ethics Committee (Reference; 8/REC/17)

### P63 Stillbirth and perinatal care: time to address the Italian gap. A professional perspective

#### Claudia Ravaldi^1^, Miriam Levi^2^, Elena Angeli^1^, Gianpaolo Romeo^2^, Marco Biffino^2^, Roberto Bonaiuti^3^, Alfredo Vannacci^3^

##### ^1^CiaoLapo Onlus, Charity for Stillbirth and Perinatal Grief Support, Prato, Italy; International Stillbirth Alliance, Prato, Italy; ^2^CeRIMP-Regional Centre for Occupational Diseases and Injuries, Tuscany Region, Florence, Italy; ^3^Department of Neurosciences, Psychology, Drug Research and Child Health (NeuroFarBa), University of Florence, Florence, Italy

###### **Correspondence:** Claudia Ravaldi

Objectives: To assess current practices of Italian health care providers (HCPs) with regard to stillbirth management and to explore their need to be trained in supporting bereaved families.

750 HCPs were administered a multiple-choice questionnaire. The results related to the items exploring behaviours and emotions of HCPs, and their opinions regarding the need for professional training courses are reported.

Compliance of Italian HCPs with international guidelines recommendations; evaluation of their cognition, emotions and behaviours with regard to stillbirth care.

The response rate was 89.9%; the majority (94.1%) were female, and mean age was 37.6 (SD = 10.4) years. Midwives were largely represented (72.8%). In case of stillbirth, only slightly more than half routinely bathe and dress the baby before allowing the parents to see them for as long as they need, whereas 44.4% usually send the child away. More than half felt inadequate and some even felt to have failed to provide support to the family when dealing with a stillbirth in the past. The need for professional training courses was expressed by 90.2%, however, three-quarters had never previously attended a course on perinatal bereavement care.

There is a substantial gap between the standards of care defined by the international guidelines and the practices currently in place in Italy. Italian perinatal HCPs feel an urgent need to be offered professional training courses to better meet the needs of grieving families.

### P64 You are not alone: ten years of self-help groups for Italian bereaved parents

#### Claudia Ravaldi^1^, Alfredo Vannacci^1,2^

##### ^1^CiaoLapo Onlus, Charity for Stillbirth and Perinatal Grief Support, Prato, Italy; ^2^Department of Neurosciences, Psychology, Drug Research and Child Health (NeuroFarBa), University of Florence, Florence, Italia

###### **Correspondence:** Claudia Ravaldi

Self-help groups are an important instrument for anyone who is affected by the loss of a baby. Until 2006 no self-help group for parents affected by perinatal loss was present in Italy. Since then CiaoLapo Charity organized several self-help groups in different parts of Italy. Here we present our ten year experience, our method as well as parents’ feedback.

Since 2007, 17 self-help groups were opened in Italy by trained CiaoLapo volunteers. Self-help groups are usually managed by a bereaved parent specifically trained (at least 2 years after their own loss) and by a counselor or a psychologist specifically trained in grief and bereavement.

We collected data from participants and from facilitators, by written interviews, during periodical meetings.

CiaoLapo self-help groups, called “Little Princes” were opened during the last 10 years in 17 Italian towns: Turin, Florence, Rome, Naples, Prato, Modena, Como, Bologna, Udine, Pordenone, Genova, Milan, Varese, Cosenza, Bergamo, Perugia, Vicenza.

Attendance of parents to group is free of charge and open; before enrolment parents need to attend a face to face meeting with a facilitator; attendance to groups usually lasts a year. Parents can choose if they want to continue for a second year or if they feel to be able to quit. Frequency might be different in different groups (usually 1-2 hours meeting every three weeks or monthly, sometimes twice a month). Groups could be attended by both parents together, or by only one of them.

CiaoLapo groups are open to parents grieving for different type of pregnancy and perinatal loss. The main keywords for attendants are: empathy, comprehension, share, respect, attention, help. Both professionals and parents increased their knowledge on this instrument for perinatal grief.

### P65 From head to heart learning in perinatal bereavement care

#### Daniel Nuzum^1,2,3^, Mary Jo Corcoran^1,3^

##### ^1^Department of Clinical Pastoral Education, Cork University Hospital, Cork, Ireland; ^2^Department of Obstetrics and Gynaecology, University College Cork, Cork, Ireland; ^3^Association of Clinical Pastoral Education (Ireland) Ltd., Dublin, Ireland

###### **Correspondence:** Daniel Nuzum

Perinatal bereavement raises deep spiritual questions for many bereaved parents. Attending to the spiritual needs of bereaved parents requires healthcare chaplains to demonstrate a high level of spiritual skill and sensitivity. Clinical Pastoral Education (CPE) is an experiential reflection–action-integration model of learning used in the education of pastoral care students.

Following theoretical input, students were facilitated to experience loss through the awareness of attachment by sharing their personal stories of hope and attachment. During this time each student passed a ball of wool through the group, thereby spinning a ‘web of attachment’ between them. Music and a recorded foetal heartbeat were playing in the background. Without warning the music and heartbeat stopped and the students were asked to drop the web of wool, which fell silently to the floor. The students were invited to reflect and to note their feelings and experiences.

There were six students and two supervisors in the participating group. The awareness and expression of hopes, attachments and expectations brought the students into the world of expectant parents. Following a creative and reflective silence the participants were able to express feelings of abandonment, fear, loss, sadness, anger, shock, emptiness and loneliness. The participants demonstrated skills of empathic awareness and spiritual sensitivity as they applied their learning in subsequent role play.

The use of multisensory and experiential teaching methodology and pedagogy as part of the CPE process enabled pastoral care students to gain a deeper understanding, awareness and learning in the area of perinatal bereavement and loss. They had entered the world of bereavement. This experience when integrated into clinical practice will enable students to care more empathically for bereaved parents and to attend to deep spiritual questions.

### P66 Preliminary analysis of respectful care after stillbirth: results from a multi-country survey (US, Canada, Australia and New Zealand)

#### Emma Sacks^1^, Frances Boyle^2^, Aleena Wojcieszek^2^, Dell Horey^3^, Lynn Farrales^4^, Vicki Flenady^2^

##### ^1^Johns Hopkins University, Washington, DC, USA; ^2^Centre of Research Excellence in Stillbirth, Mater Research Institute, The University of Queensland, Brisbane, Australia; ^3^Latrobe University, Melbourne, Australia; ^4^Department of Family Practice, University of British Columbia, Vancouver, Canada

###### **Correspondence:** Emma Sacks

Stillbirth is traumatic for families yet the study of respectful care after stillbirth, especially as related to dignified care of the infant who is stillborn, is still very new.

Secondary analysis was conducted on data from an international, online survey of parents who experienced stillbirth, disseminated primarily through member organisations of the International Stillbirth Alliance. The survey covered topics relating to experiences of stillbirth and included quantitative metrics asking whether parents and stillborn infants were treated with respect, as well as open-ended questions about care experiences. Analyses included descriptive statistics and thematic analysis.

A total of 906 mothers residing in Australia (n = 416), New Zealand (n = 44), US (n = 391) and Canada (n = 55) completed the questionnaire. The majority (approximately 80%) reported being treated with kindness and respect “always” or “most of the time”, but almost 20% experienced respectful care either “never” or “only sometimes”. Patterns were similar across the four countries; the only statistically significant difference was respondents from the US less frequently reporting respectful care of the stillborn infant (p = 0.05). Surprisingly, mothers with losses later in pregnancy (40+ weeks’ gestation) reported less respectful care. However, in more recent stillbirths (within last 5 years) mothers reported fewer negative experiences, suggesting improvements over time. Qualitatively, mothers largely expressed wanting more time with their stillborn infants and many felt rushed without good explanation for the urgency. Mothers commonly stated that many of their questions were not answered, including why certain events occurred, options for burial/cremation, autopsies and concerns over their own health.

While care practices are improving, many mothers reported non-respectful care at some point. Lack of communication, including lack of informed consent, is critical to improving care after stillbirth. More research is needed on the burden of disrespectful care (including in low-income settings) and the care practices desired by families.

Ethics approval was granted by the Mater Health Services Human Research Ethics Committee on 29th November 2013 (Ref #HREC/13/MHS/121) and by the University of British Columbia Office of Research Services, Behavioural Research Ethics Board on 22nd December 2014 (Ref #H14-02784). Completion of the anonymous online survey indicated consent to participate in the study.

### P67 Fetal deaths in Brazil: spatial distribution of a time series

#### Flavio Ibiapina, Aline Veras, Raimunda Magalhães, Rosa Almeida

##### University of Fortaleza, Fortaleza, Brazil

###### **Correspondence:** Flavio Ibiapina

In a country of continental dimensions such as Brazil, discussing the distribution of fetal deaths by region contributes to the planning of public policies. This study aims to describe the causes of fetal death according to groups of preventable causes in different regions of Brazil from 2001 to 2014.

**Fig. 8 (abstract P67).**
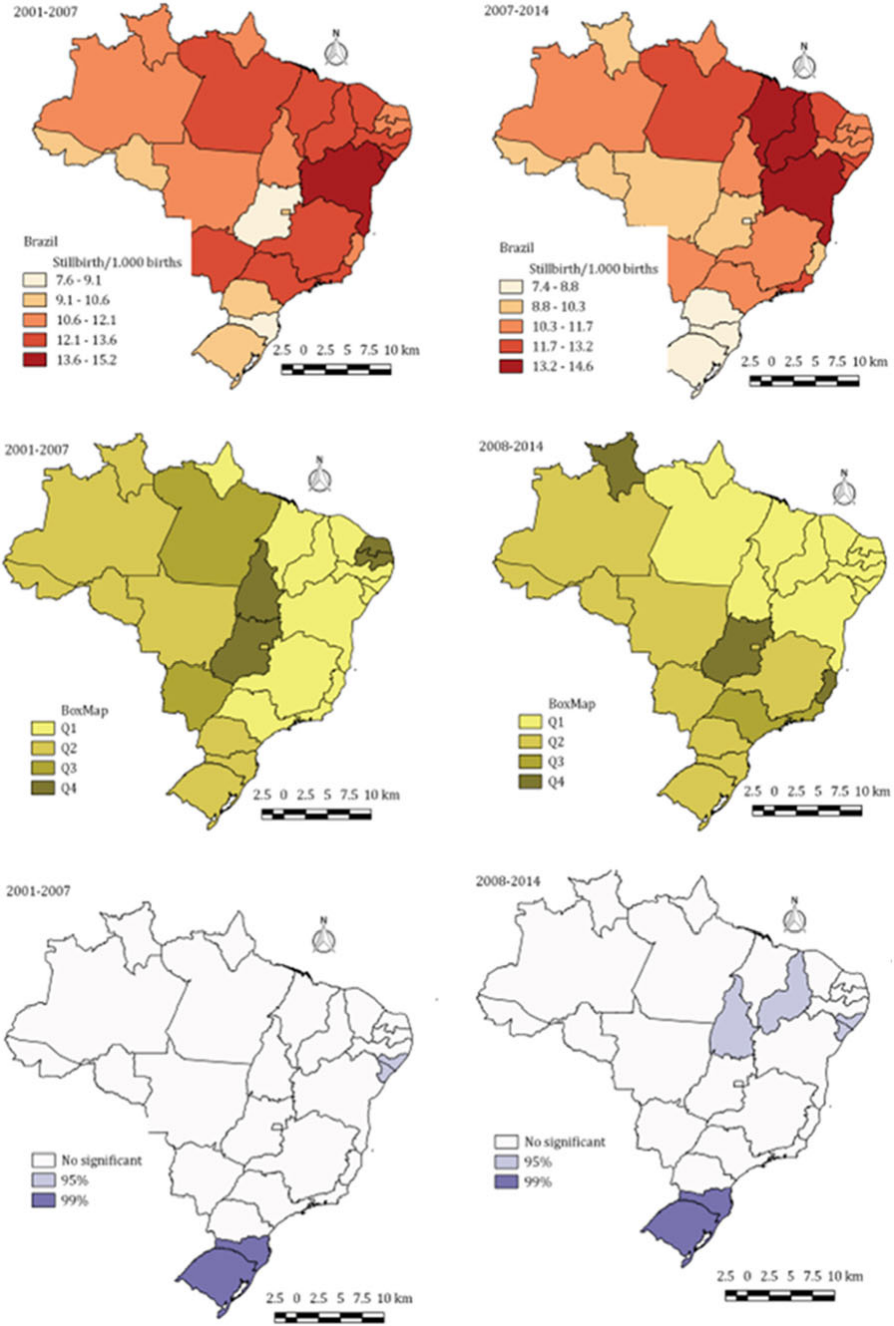
© [2017] [Ibiapina, Flavio et al]

An ecological study focused on a spatial analysis of records of fetal deaths from 2001 to 2014 in a national database. Fetal mortality rates were calculated and aggregated by Federation Units. Data normality was checked by the Shapiro-Wilk test. For spatial analysis, a neighborhood matrix was created using the contiguity criterion. A Moran Map was created and it displays four associations: Q1 (high-high), with locations with a high incidence rate and a neighbor with a high incidence rate; Q2 (Low-Low), neighboring regions with low rates; and Q3 (high-low) and Q4 (low-high), with locations with non-similar neighborhoods, reflecting transition areas. A Lisa Map was created in QGIS 2.18.3 to identify aggregates (Fig. 8).

465,050 deaths were analyzed. The average death rate was 11.3 deaths/thousand births. The Northeast region has the highest average rate: 12.9/1000 births. In the Northeast and North regions, deaths that could have been avoided by adequate care of the fetus and newborns were predominant. The Southeast region presented a prevalence of deaths that could have been avoided by adequate care of the woman during labor. In the South region, there was a predominance of deaths that could have been avoided by adequate care of the woman during pregnancy. The spatial analysis showed a rate distribution in two periods. Significant clusters and areas of greatest risk for fetal death in Brazil are identified.

Interventions aimed at reducing fetal mortality depend on structural changes related to living conditions and on direct actions defined by public health policies.

### P68 Perinatal palliative care – a guide to anticipatory bereavement care for parents

#### Heather McGovern-Silver

##### Silverleaf Consulting Services, Llc., Severna Park, MD, USA

Parents who are given the news that their baby has a life-threatening diagnosis often experience feelings of fear, anxiety, isolation, and the vast unknown. Overwhelmed and consumed by questions, they search for answers often turning to the internet for information, causing more confusion and complications. A culture needs to be created to support and guide families with the right tools and appropriate resources to provide insight for a healthy journey through loss.

A search of available literature shows gaps across many healthcare systems for providing psychosocial and spiritual support, which research has linked to parents having a negative emotional impact. The purpose of this presentation is to provide and discuss research based findings and clinical expertise to offer best practices immediately following diagnosis, providing anticipatory bereavement, giving options for pregnancy, birth, legacy building and aftercare of mom and baby.

In our almost 60 years combined clinical experience parents and families who have received appropriate anticipatory bereavement care share a sense of peace and memories of compassionate caregiving. Those families who felt their needs were not met frequently suffer from unresolved grief. The reviewed research, along with our expert clinical social work and bedside nurse experience, give us the passion to provide insight into walking alongside these special families as they prepare for the birth and death of their precious babies.

Families who are given the opportunity to process the difficult news of a life-threatening pregnancy diagnosis, presented with a guided approach for decision making which includes physical, emotional and spiritual options and needs are able to have control over many decisions and actions in an “out of control” situation. They often report having a positive experience within a healthcare system and feel well cared for, even when there is a negative outcome.

### P69 Perinatal autopsy for stillbirth ≤ 20 weeks

#### Karen Gibbins^1^, Jessica Comstock^2^, Yajing Xiong^3^, Robert Silver^1^

##### ^1^University Of Utah, Salt Lake City, UT, USA; ^2^Primary Children’s Intermountain Healthcare, Salt Lake City, UT, USA; ^3^University of Utah School of Medicine, Salt Lake City, UT, USA

###### **Correspondence:** Karen Gibbins

Utility of perinatal autopsy in ascertaining a potential cause of death is well established in evaluation of stillbirth after 20 weeks gestation. However, autopsy is not routinely performed prior to 20 weeks’ gestation, and these early fetuses may be too immature to glean useful information. Our objective was to describe our experience with perinatal autopsy in fetuses ≤ 20 weeks’ gestation.

Descriptive study of 211 fetal autopsy reports with gestational age listed as 20 weeks or fewer (2000-2015). Demographics, indication for delivery (antepartum stillbirth (AS), intrapartum stillbirth (IS), or termination for fetal anomalies), mode of delivery, gestational age estimates, degree of maceration, and autopsy findings were abstracted. Autopsy findings were categorized by organ system and recorded as normal, abnormal, or unable to determine.

**Fig. 9 (abstract P69).**
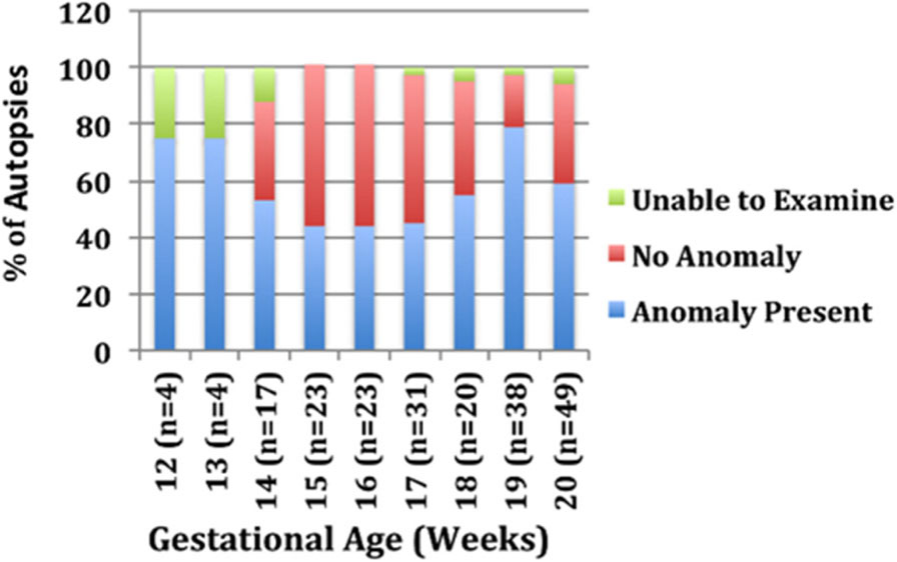
Anomalies detected at each gestational week

Mean gestational age was 17.4 weeks. 67% were stillbirths, 13% previable spontaneous labor, and 20% terminations. Delivery was via spontaneous vaginal delivery in 191 (92%), Cesarean delivery in 2 (1%) and dilation & evacuation (D&E) in 13 (6.3%). Maceration ranged from none in 31% to grade IV in 42%. Anomalies were detected in 122 (58%): skeletal anomalies in 30%, head and neck 23%, abdominal 22%, chest 21%, cardiac 21%, genitourinary 13%, and central nervous system (CNS) 8%. Only 10 fetuses (5%) were too macerated or damaged to permit useful evaluation. Detection of anomalies by gestational age is shown in Fig. 9. In fetuses with grade IV maceration, 39 (46%) had an anomaly detected. In D&E cases, anomalies were detected in 54%, although 38% were unable to be evaluated. CNS anomalies were unable to be evaluated in 110 (55%).

Fetal autopsy yields useful results in fetuses below 20 weeks’ gestation. Due to rapid postmortem liquefaction of the brain in fetuses, CNS autopsy is limited, but other organ systems remain discernable, even with significant maceration.

Ethical approval for the study was granted by all Institutional Review Boards (IRB) of participating centers. University of Utah IRB approval #00093587, initial approval date 8/23/2016; Intermountain Healthcare IRB approval #1050257, initial approval date 7/14/2016.

### P70 Risk factors for stillbirth due to placental insufficiency at term

#### Karen Gibbins^1^, Robert Silver^1^, Halit Pinar^2^, Corette Parker^3^, Vanessa Thorsten^3^, Donald Dudley^4^, Radek Bukowski^5^, George Saade^6^, Deborah Conway, Carol Hogue^8^, Barbara Stoll^8^, Robert Goldenberg^9^

##### ^1^University Of Utah, Salt Lake City, UT, USA; ^2^The Warren Alpert Medical School of Brown University, Providence, RI, USA; ^3^RTI International, Durham, NC, USA; ^4^University of Virginia, Charlottesville, VA, USA; ^5^Yale-New Haven Hospital, New Haven, CT, USA; ^6^University of Texas Medical Branch, Galveston, TX, USA; ^7^University of Texas, San Antonio, TX, USA; ^8^Emory University, Atlanta, GA, USA; ^9^Columbia University, New York, NY, USA

###### **Correspondence:** Karen Gibbins

Placental insufficiency (PI) is a leading cause of stillbirth (SB). At term, SB due to PI (PISB) can be prevented by delivery if detected. Our goal was to compare exposures between women with term PISB and women with live births (LB).

The Stillbirth Collaborative Research Network (SCRN) conducted a population based, case-control study of SBs and LB from 2006-2008. This analysis includes term births only and compares PISB to LB. PISB was ascertained using the Initial Cause of Fetal Death (INCODE) classification tool developed by the SCRN. We compared demographic, obstetric, and prenatal factors. Analyses were weighted to account for the original study design, differential consent for participation, and availability of placental histology. Weighted frequencies, crude odds ratios (ORs), and adjusted ORs (aORs) are reported.

After weighting, there were 1550 term LB to compare to 25.1 PISB. Non-Hispanic black race (aOR 5.64, 95% CI 1.47-21.58) and Hispanic ethnicity (aOR 3.73, 95% CI 1.07-13.07) were associated with increased odds of PISB (Table 10). Maternal birth in the United States had a lower risk of PISB (aOR 0.18, 95% CI 0.06-0.54). Women without insurance had much higher odds of PISB than women (aOR of 18.32, 95% CI 2.80-119.92). Medical conditions with increased risk of PISB include asthma (aOR 2.84, 95% CI 1.03-7.79), diabetes (aOR 7.22, 95% CI 1.90-27.41), and hyperthyroid disease (aOR 9.48, 95% CI 2.26-39.73). Pregnancy specific factors including nulliparity (aOR 6.85, 95% CI 2.74-17.15) and antenatal bleeding (aOR 7.80, 95% CI 2.54-23.93) also increased risk of PISB.

At term, multiple factors are associated with increased risk of PISB. Although some risk factors are disease states, many risk factors suggest decreased access to care (race/ethnicity, lack of insurance, non-native birth) and systemic disparities.

Ethical approval for the study was granted by all Institutional Review Boards (IRB) of participating centers. University of Utah IRB approval #00014353, initial approval date 9/25/2005. Intermountain Healthcare IRB approval #107704, initial approval date 10/19/2005. Written informed consent was obtained by all study participants.

**Table 10 (abstract P70).** Prenatal and antenatal differences in women with SBs, with and without evidence of placental insufficiency by INCODE*

	PI by INCODE N = 121	Non-PI SB N = 391	p-value
Prenatal care	110 (95.7)	345 (93.5)	0.395
BMI (kg/m^2^)	28.5 (7.1)	27.6 (6.8)	0.213
Obese	41 (37.6)	94 (31.1)	0.216
Maternal comorbidities			
HTN	17 (14.8)	37 (10)	0.154
Asthma	8 (7.0)	38 (10.3)	0.289
Seizures	0 (0)	6 (1.6)	0.344
DM	7 (6.1)	20 (5.4)	0.781
Hyperthyroid	3 (2.6)	1 (0.27)	0.043
Hypothyroid	2 (1.7)	8 (2.2)	1.000
Kidney disease	1 (0.9)	6 (1.6)	1.000
Sickle cell	0 (0)	4 (1.1)	0.577
Autoimmune disease (SLE/APS/RA/UC/Crohn’s)	2 (1.8) --	0 (0) --	0.055 --
Mental illness	11 (9.6)	26 (7.0)	0.370
UTI	23 (20)	49 (13.2)	0.075
Blood clotting disorder	2 (1.7)	3 (0.8)	0.340
ART	4 (3.4)	14 (3.7)	1.000
Alcohol	3 (2.5)	10 (2.6)	1.000
Tobacco	20 (16.8)	42 (10.9)	0.089
Drug use	4 (3.4)	11 (2.9)	0.759
Abuse (physical, sexual, or emotional)	2 (1.9)	5 (1.6)	1.000
Antenatal bleeding	11 (9.6)	35 (9.5)	0.980
GHD	28 (23.1)	45 (11.5)	0.001
SGA	48 (39.7)	64 (16.4)	<0.001
Gestational age of SB	29.4 (6.6)	28.1 (6.6)	0.056
Intrapartum demise	6 (5.5)	38 (11.0)	0.086

HTN = hypertension; DM = Diabetes mellitus; SLE = Systemic Lupus Erythematosus; APS = Antiphospholipid syndrome; RA = Rheumatoid arthritis; UC = ulcerative colitis; ART = Assisted Reproductive Technology

Data are reported as n (%) or mean (SD). P-values are generated via t-test, chi-square test, or Fisher’s exact test.

*Dudley DH et al. A new system for determining the causes of stillbirth. Obstet Gynecol 2010; 116: 254-260.

### P71 Predicting pregnancy outcome in women with a history of prior stillbirth

#### Nicole Graham^1,2^, Louise Stephens^1,2^, Edward Johnstone^1,2^, Alexander Heazell^1,2^

##### ^1^Maternal and Fetal Health Research Centre, Division of Developmental Biology and Medicine, Faculty of Biology Medicine and Health, University of Manchester, Manchester, UK; ^2^Departement of Obstetrics, St Mary’s Hospital, Manchester, M13 9WL, UK

###### **Correspondence:** Nicole Graham

Efforts to reduce stillbirth in high-income countries have focussed on improving care for women at increased risk. One such group is women who have had a stillbirth in their preceding pregnancy, who have an almost 5-fold increased risk. The origins of this increased risk are not well understood. This study aimed to explore the relationship between the cause of index stillbirth and subsequent pregnancy outcome.

A retrospective cohort study was conducted; cases were included if the stillbirth was investigated and the subsequent pregnancy care was in the same tertiary maternity unit. Stillbirths were classified using the ReCoDe system. All women had ultrasound assessment of placental biometry and uterine artery Doppler at 23 weeks’ gestation in the subsequent pregnancy followed by regular assessment of fetal growth.

120 cases were identified, 13 were excluded (mothers were still pregnant/outcome data incomplete). In this cohort (n = 107), there were no recurrent stillbirths, but 16 adverse outcomes (15%) including 2 second trimester miscarriages and 1 neonatal death. Overall, thirteen infants delivered preterm (<37 wks’) and eight infants were admitted to the neonatal intensive care unit. Fetal growth restriction was identified in 7 out of 16 adverse outcomes.

In the 16 adverse outcomes, the prior stillbirth was associated with placental dysfunction in 11 cases and fetal anomaly in 1 case. No adverse outcomes were seen with prior unexplained stillbirth. 6 cases of adverse outcome had an abnormal placental ultrasound, all of which had placental dysfunction in the index stillbirth.

Adverse outcome is more likely if the index stillbirth was associated with placental insufficiency. Placental ultrasound can detect recurrent placental abnormalities in a significant proportion of subsequent pregnancies. Conversely, unexplained stillbirths or those associated with fetal anomaly or infection are less likely to have adverse outcome in a subsequent pregnancy.

Ethical approval for the study was granted by South East Coast- Surrey Research Ethics Committee (Ref 16/LO/1666).

### P72 Abnormal placental cord insertion and risk of intrauterine fetal death: a systematic review and meta-analysis

#### Khadijah Irfah Ismail^1^, Ailish Hannigan^2^, Keelin O’Donoghue^3^, Amanda Cotter^1^

##### ^1^Obstetrics and Gynaecology, Graduate Entry Medical School, University Of Limerick, Limerick, Ireland; ^2^Biostatistics Department, Graduate Entry Medical School, University of Limerick, Limerick, Ireland; ^3^Obstetrics and Gynaecology, University College Cork, Cork, Ireland

###### **Correspondence:** Khadijah Irfah Ismail

Abnormal placental cord insertions including velamentous cord insertion (VCI) and marginal cord insertion (MCI) have been associated with adverse pregnancy outcomes including small for gestational age infants, preterm birth and intrauterine fetal death. We systematically reviewed and meta-analysed studies of abnormal placental cord insertions and the association with intrauterine fetal death.

Embase, Medline, CINAHL, Scopus, Web of Science and Cochrane Databases were searched in September 2016 (from inception to September 2016). Studies which contained the following: singleton pregnancies, VCI, MCI and intrauterine fetal death. Potentially eligible studies were reviewed by two authors independently. The quality of included studies was assessed using the Newcastle-Ottawa Quality Assessment Scale. Summary risk ratios with 95% confidence intervals were calculated.

**Fig. 10 (abstract P72).**
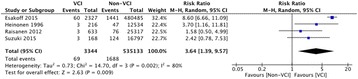
See text for description

Four studies were available for analysis of VCI compared to non-VCI for the outcome intrauterine fetal death. No studies reporting the outcome intrauterine fetal death comparing VCI, MCI and normal placental cord insertion were identified. Meta-analysis of the four studies showed a statistically significant increased risk of intrauterine fetal death (pooled RR, 3.64; 95% CI, 1.39 -9.57, P = 0.009) for the VCI group compared to the non-VCI group with evidence of significant heterogeneity (χ2 = 14.7, P = 0.002, I2 = 80%), so a random effect model was used (Fig. 10).

The available evidence suggests an association between VCI and intrauterine fetal death. The association with MCI remains unclear. Further studies to identify the risk of intrauterine fetal death with MCI are needed.

### P73 My Baby’s Movements: A stepped-wedge, cluster-randomised controlled trial testing a mobile application intervention aimed at lowering stillbirth rates

#### Vicki Flenady^1^, Glenn Gardener^1^, David Ellwood^2^, Philippa Middleton^3^, Fran Boyle^1^, Caroline Crowther^4^, Michael Coory^1^, Chris East^13^, Emily Callander^6^, Adrienne Gordon^7^, Jonathan Morris^7^, Victoria Bowring^8^, Alison Kent^10^, Sue Vlack^9^, Glyn Teale^5^, Lisa Daly^1^, Sarah Henry^1^, Hanna Reinebrant^1^, Aleena Wojcieszek^1^, Frederik Froen^11^, Jane Norman^12^

##### ^1^Mater Research Institute- University of Queensland, Brisbane, Australia; ^2^Griffith University, Brisbane, Australia; ^3^South Australian Health and Medical Research Council, Adelaide, Australia; ^4^University of Auckland, Auckland, New Zealand; ^5^University of Melbourne, Melbourne, Australia; ^6^James Cook University, Townsville, Australia; ^7^University of Sydney, Sydney, Australia; ^8^Stillbirth Foundation Australia, Annandale, Australia; ^9^School of Population Health, University of Queensland, Brisbane, Australia; ^10^Australian Capital Universities, Canberra, Australia; ^11^Norwegian Institute of Public Health, Oslo, Norway; ^12^Tommy’s Centre for Maternal Fetal Health, University of Edinburgh, Edinburgh, Scotland; ^13^Monash University, Melbourne, Australia

###### **Correspondence:** Vicki Flenady

Stillbirth is a major global public health problem. Maternal perception of decreased fetal movements (DFM) is often the only warning sign. The My Baby’s Movements (MBM) trial aims to reduce stillbirth rates using a mobile platform to enhance maternal knowledge about decreased fetal movement and encourage timely health-care seeking behaviour combined with a clinician education program on management of DFM.

A stepped-wedge, cluster-randomised design, involving 26 hospitals (8 clusters) and 260,000 women in Australia and New Zealand (ANZ) over a 3-year period (2016-2018). The primary outcome measure is stillbirth rates after 28 weeks’ gestation will be reduced by 30%. Satisfaction with the app is sought from users through a brief survey incorporated into the app.

The MBM mobile application has been developed, after pilot testing including iterative cycles of testing and improvement. App content is based on the ANZ DFM Clinical practice guidelines and brochure for women. MBM implementation is underway with results due in 2019. Initial feedback from users is very positive. The SWCRCT is an ideal design for large scale trials required to address stillbirth providing a pragmatic while robust evaluation model.

The My Baby’s Movements trial will generate information about fetal movement awareness, health promotion and perinatal health outcomes associated with a mobile application intervention. Preliminary data suggests that the mobile phone app is well received by users.

Ethical approval for the study was granted by the Mater Health Services Human Research Ethics Committee (Reference; HREC/14/MHS/141 AM02) and seven additional HREC’s across Australia and New Zealand.

### P74 Post-hospital care after stillbirth in Australia and New Zealand: how well are women’s needs met?

#### Fran Boyle^1,2,3^, Jade Ratnayake^4^, Dell Horey^1,2,3,5^, Vicki Flenady^1,2,3^

##### ^1^Centre of Research Excellence in Stillbirth, Brisbane, Australia; ^2^Mater Research Institute - University Of Queensland, South Brisbane, Australia; ^3^International Stillbirth Alliance, Bristol, UK; ^4^School of Public Health, University of Queensland, Herston, Australia; ^5^College of Science, Health & Engineering, La Trobe University, Melbourne, Australia

###### **Correspondence:** Fran Boyle

The Lancet 2016 Ending Preventable Stillbirths series focused attention on the importance of respectful and supportive care for parents faced with the tragedy of stillbirth. Most studies of care after stillbirth centre on the hospital stay. Research into care once parents leave hospital is scarce. This paper reports on mothers’ experiences and perceptions of post-hospital care after stillbirth in Australia and New Zealand and identifies areas of unmet need.

The data source was a large multi-country survey of parents who had experienced stillbirth conducted between December 2014 and February 2015. An online questionnaire was distributed through member organisations of the International Stillbirth Alliance, including parent support organisations in Australia and New Zealand. Responses to rating scale items about the type and quality of post-hospital services received by women and an open-ended question asking how care might have been improved were analysed.

460 mothers from Australia and New Zealand completed the survey. Less than half (47%) viewed their follow-up care positively and almost one-third (30%) rated their follow-up care as “poor” or “very poor”. More recent stillbirths (within the last 5 years) were associated with more positive ratings of follow-up care. Most mothers (79%) provided a free-text response about how care could have been improved. The importance of sensitive, respectful care that began in the hospital and followed women into the community was paramount. Prominent themes included: “not being forgotten”; “being treated as a mother”; and “pathways to support”.

Post-hospital care after stillbirth appears to fall short of best practice for many mothers. A need exists for more comprehensive bereavement training for hospital staff and attention to organisational aspects of care to support the hospital-to-home transition. Explicit recognition of the role of hospital staff in facilitating ongoing support for women is required.

Ethics approval was granted by the Mater Health Services Human Research Ethics Committee on 29th November 2013 (Ref #HREC/13/MHS/121) and by the University of British Columbia Office of Research Services, Behavioural Research Ethics Board on 22nd December 2014 (Ref #H14-02784). Completion of the anonymous online survey indicated consent to participate in the study

### P75 Understanding parents’ decision-making needs for autopsy consent after stillbirth: a view from Australia and New Zealand

#### Anne Schirmann^1,4^, Fran Boyle^1,2,3^, Dell Horey^1,2,3,5^, Dimitrios Siassakos^3,6^, Ingrid Rowlands^4^, David Ellwood^1,3,7^, Vicki Flenady^1,2,3^

##### ^1^Centre of Research Excellence in Stillbirth, Brisbane, Australia; ^2^Mater Research Institute - University Of Queensland, Brisbane, Australia; ^3^International Stillbirth Alliance, Bristol, UK; ^4^School of Public Health, University of Queensland, Brisbane, Australia; ^5^College of Science, Health & Engineering, La Trobe University, Melbourne, Australia; ^6^Academic Centre for Women’s Health, University of Bristol, Bristol & Southmead Hospital, Bristol, UK; ^7^Griffith University and Gold Coast University Hospital, Gold Coast, Australia

###### **Correspondence:** Anne Schirmann

Supporting parents in autopsy decision-making is an essential but challenging part of quality care after stillbirth. Parent-centred information that is clear, consistent and sensitive is needed to guide decisions and minimise the likelihood of later regret. Developing effective decision support tools requires understanding of the complex decision environment in which parents’ deliberations take place and the range and nature of influencing factors.

Data were from the ISA Lancet survey, a large multi-country survey of parents of stillborn infants conducted between December 2014 and February 2015. An online questionnaire covered various topics related to the experience of stillbirth and was distributed through ISA member organisations, including parent support groups in Australia and New Zealand. The framework method was used to qualitatively analyse mothers’ responses to open-ended questions about autopsy.

460 mothers from Australia and New Zealand participated: 454 mothers provided one or more free-text responses referencing autopsy yielding more than 1,200 data segments for analysis.

The data confirmed the immensely difficult decision that autopsy consent entails. Mothers had a strong need for answers coupled with a strong need to protect their baby. Four “decision drivers” were confirmed: preparedness for the decision; parental responsibility; possible consequences; and role of health professionals. Each had the capacity to influence a decision for or against autopsy. Also prominent were the “aftermath” of the decision: receiving the results; and decisional regret or uncertainty.

The influences on parents’ decision-making regarding autopsy are complex and multifaceted. The range of influences implies the need for tailored information that addresses different parent needs. These findings are an initial step in the development of a decision support tool applicable to Australian and New Zealand settings.

Ethics approval was granted by the Mater Health Services Human Research Ethics Committee on 29th November 2013 (Ref #HREC/13/MHS/121) and by the University of British Columbia Office of Research Services, Behavioural Research Ethics Board on 22nd December 2014 (Ref #H14-02784). Completion of the anonymous online survey indicated consent to participate in the study.

### P76 Women who seek care for decreased fetal movements - maternal characteristics and onset of labour

#### Anders Linde^1,2^, Karin Pettersson^1^, Ingela Rådestad^2^

##### ^1^Karolinska Institutet, Stockholm, Sweden; ^2^Sophiahemmet University, Stockholm, Sweden

###### **Correspondence:** Anders Linde

Pregnant women seeking care due to decreased fetal movements are relatively common in obstetric care. The aim of this study was to investigate characteristics of women who seek consultation due to decreased or altered fetal movements in late pregnancy and onset of labour.

All women with a simplex pregnancy (gestational week 28+), who sought care in Stockholm, Sweden, in 2014 due to concerns for decreased or altered fetal movements were asked to complete a questionnaire. Information on perinatal outcome was collected from medical records. The control group comprised of women who gave birth after the pregnancy week of 28 + 0 during 2014 in Stockholm.

A total of 2683 women with decreased or altered fetal movements and 26041 women in the control group were included. Women who sought care due to decreased or altered fetal movements were younger, ≤ 19-24 (p = 0.005) and more often primipara (p < 0.001) compared with the women in the control group. Women born in Sweden sought care more often than women born outside Sweden (p < 0.001). Women with a low educational level, primary school or equivalent, did not seek care to the same extent as women with a higher educational level; (p < 0.001). A higher proportion of women who sought care had a BMI between 30 to 34.9 compared to women in the control group; (p < 0.001). One in four women who sought care for decreased or altered fetal movements had the delivery induced compared to 17.4 percent of the women in the control group (p < 0.001). A dose-response effect was noted for the number of times women sought care and rate of induction.

Pregnant women seeking care due to decreased or altered fetal movements are induced more often compared to women who do not seek care for decreased fetal movements.

Ethical approval for the study was granted by Stockholm Research Ethics Committee of Sweden(Reference; Dnr 2013/1077-31/3. Written informed consent was obtained by all study participants.

### P77 Fetal growth restriction among stillbirths and its antenatal detection in Ireland: a national clinical audit

#### Paul Corcoran, Edel Manning, Irene O’Farrell, Sarah Meaney, Richard Greene

##### National Perinatal Epidemiology Centre, University College Cork, Cork, Ireland

###### **Correspondence:** Paul Corcoran

Fetal growth restriction may be the greatest contributing factor to stillbirth but antenatal detection of it during routine maternity care is poor. We sought to estimate the prevalence and the level of antenatal detection of fetal growth restriction among stillbirths in Ireland.

As part of a national clinical audit of perinatal mortality, contributors in all 20 Irish maternity units completed and submitted detailed notification forms related to stillbirths in 2011-2015. We calculated the stillbirth rate per 1,000 births using several criteria for defining stillbirths. We derived customised birthweight centiles using the Gestation Related Optimal Weight (GROW) software. Stillbirths <10th customised birthweight centile were considered small for gestational age (SGA) and those <3rd centile were considered severely SGA.

**Fig. 11 (abstract P77).**
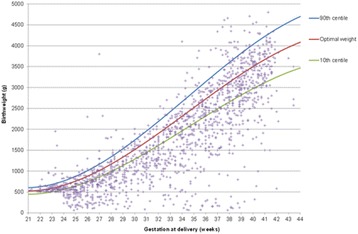
Optimal birthweight and normal range compared to actual birthweights of stillbirths in Ireland, 2011-2015

There were 1,547 notifications of stillbirths delivered in 2011-2015 after 24 weeks gestation or with a birthweight ≥500g. Depending on the case-definition criteria, the stillbirth rate ranged from 3.4 to 4.7 per 1,000 births. Forty-two percent (637/1535) of the stillbirths were severely SGA and 54.6% were SGA (837/1535) see Fig. 11. SGA was more prevalent among the stillbirths complicated by multiple pregnancy, maternal hypertension and congenital anomaly and in stillbirths delivered pre-term. Antenatal detection was at 20% (167/833) for SGA and 25% (478/633) for severely SGA. Antenatal detection in the 20 maternity units was broadly consistent with the national level. Antenatal detection varied little across a range of factors but was almost twice as common if a congenital anomaly was present (29% vs. 16%).

Antenatal detection of fetal growth restriction among stillbirths in Ireland is poor. Standardised ultrasound services involving two examinations and customised fetal growth charts should be provided for all pregnant women in Ireland.

Ethical approval for the NPEC national clinical audits of obstetric outcomes was provided by the Clinical Research Ethics Committee of the Cork Teaching Hospitals (Reference: ECM 4(g) 05/08/08)

### P78 Analysis of stillbirth as a major public health problem in Mali

#### Mamadou Berthe

##### Direction Nationale De La Santé, Marseille, France

Stillbirths estimated at 2.6 million in 2015 in developing countries are a public health problem. It is defined as the total number of stillbirths in the total number of births reported over a given period. A stillbirth is defined by WHO as a lifeless born fetus weighing ≥ 1000g and gestational age is ≥ 28 weeks of amenorrhea.

Thus, we proposed this analysis of the routine data of stillbirths registered in health facilities in Mali over a period of nine years.

This is a retrospective descriptive cross-sectional study. We used routine data from 1 January 2008 to 31 December 2016 in the Community Health Centers (first level of the health system) and Reference Health Centers (the first reference).

In total 94495 deaths were reported by first-level health institutions with an average of 10497, a median of 9934, the extremes range from 8449 in 2009 to 13448 in 2008 (see Table 11).

From 2008 to 2016 4 102 691 deliveries and 94475 stillbirths, ie a rate of 2,30%. The region of Sikasso has the highest rate (2.88%), followed by Gao and Timbuktu respectively 2.87 and 2.70.

These data from the routine health information system can be supplemented by an analytical study to better explain these high stillbirth rates in the country in order to take adequate measures.

**Table 11 (abstract P78).** Distribution by region of the number of stillbirths, number of deliveries and stillbirth rates from 2008 to 2016 in Mali

**Regions**	**Number of stillbirths**	**Number of deliveries**	**Rate of stillbirths**
**Kayes**	9531	499617	1,91
**Koulikoro**	16965	751892	2,26
**Sikasso**	24210	840856	2,88
**Ségou**	15476	648786	2,39
**Mopti**	8926	432395	2,06
**Tombouctou**	2083	77135	2,70
**Gao**	1414	49195	2,87
**Kidal**	96	4999	1,92
**Bamako**	15774	797816	1,98
**Mali**	**94475**	**4102691**	**2,30**

### P79 Framework for respectful care after stillbirth

#### Dell Horey^1,2,3,4^, Fran Boyle^2,3,4^, Vicki Flenady^2,3,4^, Anne Schirmann^2,5^

##### ^1^College of Science, Health & Engineering, La Trobe University, Bundoora, Australia; ^2^Centre of Research Excellence in Stillbirth, Brisbane, Australia; ^3^Mater Research Institute - University Of Queensland, South Brisbane, Australia; ^4^International Stillbirth Alliance, Bristol, UK; ^5^School of Public Health, University of Queensland, Herston, Australia

###### **Correspondence:** Dell Horey

Care after stillbirth is a main focus area of the new Centre of Research Excellence (CRE) in Stillbirth in Australia. This area of practice is complex, multifaceted, not well-defined and largely informed by evidence that is fragmented. The use of diverse approaches to describe or define areas of practice is common across health-care and is recognised as a significant impediment to high quality research evidence by creating barriers to evidence synthesis and research collaboration. Such challenges are evident in several systematic reviews related to care after stillbirth published in recent years that include recommendations that either encompass the broad scope of care after stillbirth or focus on isolated aspects of care. It can be difficult to consolidate key messages from different reviews because of the different approaches taken.

In other health areas, one successful strategy used to overcome similar problems has been to develop conceptual frameworks to guide research directions. The CRE aimed to develop a framework focussing on the goals of respectful care after stillbirth that could describe different activities and how they interact.

We used a multi-stage process involving clarification of the goals of respectful care after stillbirth through a review of the literature and analysis of parent responses to a large online multi-country survey about stillbirth. The initial draft framework was presented to parents and clinicians at the 2016 Perinatal Society of Australian and New Zealand (PSANZ) conference in Townsville.

**Fig. 12 (abstract P79).**
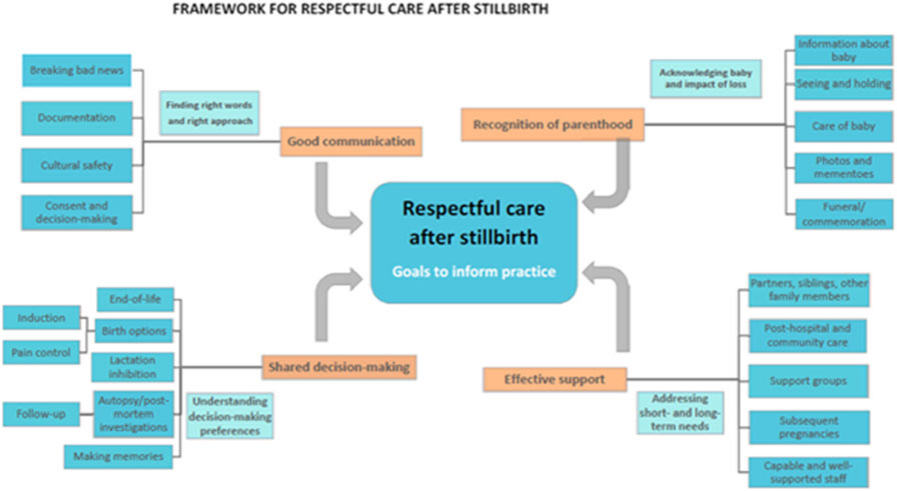
Framework for respectful care after stillbirth

The framework for respectful care after stillbirth addresses four main goals of care: good communication; shared decision-making, recognition of parenthood; and effective support. Each goal has associated practice areas (Fig. 12).

The framework shows how the context for respectful care can be used to conceptualise and evaluate different strategies to support parents who experience stillbirth and those that care for them.

Ethics approval pertaining to survey data was granted by the Mater Health Services Human Research Ethics Committee on 29th November 2013 (Ref #HREC/13/MHS/121) and by the University of British Columbia Office of Research Services, Behavioural Research Ethics Board on 22nd December 2014 (Ref #H14-02784). Completion of the anonymous online survey indicated consent to participate in the study. Ethics approval was not required for other data sources, which were drawn from published materials available in the public domain.

### P80 “Anticipating death at birth”: perinatal palliative care in a maternity hospital setting

#### Anna Maria Verling^1,2^, Orla O’Connell^1,2^, Noirin Russell^1,2^, Keelin O’Donoghue^1,2,3^

##### ^1^Pregnancy Loss Group, Department of Obstetrics & Gynaecology, University College Cork, Cork, Ireland; ^2^Cork University Maternity Hospital, Cork, Ireland; ^3^Irish Centre for Fetal and Neonatal Translational Research (INFANT), University College Cork, Cork, Ireland

###### **Correspondence:** Anna Maria Verling

Perinatal palliative care involves the provision of supportive care for a baby and their family during pregnancy and after delivery when a life limiting condition is diagnosed. The uncertainty these parents face requires a comprehensive and individualised approach from the time of diagnosis through delivery and beyond, which in our hospital is provided by the multidisciplinary bereavement team.

This was a retrospective review from 2012-2016 of all pregnancies with prenatally-diagnosed life-limiting fetal anomaly, which resulted in stillbirth or neonatal death. We excluded pregnancies diagnosed within 1-2 weeks of delivery, or those where the team had limited involvement. Cases were identified from birth registers and clinic records, with data supplemented by individual chart review. Data were analysed using Microsoft Excel.

There were 73 pregnancies with life-limiting fetal anomaly (including 10 multiple pregnancies) where parents chose to continue their pregnancy and were managed by the multidisciplinary team. Diagnosis was at a median gestation of 20 weeks (range; 12-28) and included anencephaly (9), major trisomy (20), renal agenesis (9), triploidy (4), thanatophoric dysplasia (2) and complex congenital heart disease (6). Thirty-eight infants (38/73; 52%) were stillborn and delivered at a median gestation of 32 weeks (range;23-40 + 2). The remaining infants delivered at a median gestation of 36weeks (range; 28-39). Twenty-four died on the first day of life, 9 died between day 2 and 6 and two infants died at home on days 7 and 11 respectively.

The multidisciplinary team approach is based on respect, dignity and compassion which focuses on parental choice. Working with parents collaboratively affirms the value of their baby’s life and also their identity as parents. With 14 pregnancies managed by the team in a maternity setting annually, skilled expertise has developed in caring for families following antenatal diagnosis of life-limiting fetal anomalies.

Ethical approval for the study was granted by Clinical Research Ethics Committee of the Cork Teaching Hospitals (Reference No: ECM 6(aa) 06/01/15).

### P81 Audit of quality of care provided to parents who experienced stillbirth in a hospital setting

#### Claire Everard^1,2^, Simon Long^3^, Keelin O’ Donoghue^1,2,4^

##### ^1^Pregnancy Loss Research Group, Department of Obstetrics and Gynaecology, University College Cork, Cork, Ireland; ^2^Cork University Maternity Hospital, Cork, Ireland; ^3^Department of Computer Science, Cork Institute of Technology, Cork, Ireland; ^4^Irish Centre for Fetal and Neonatal Translational Research (INFANT), Cork, Ireland

###### **Correspondence:** Claire Everard

Clinical audit is a valuable tool which can shape education, improve healthcare delivery and pinpoint areas for future research. Our aim was to develop a customised audit tool to review of the quality of care provided to parents who experience stillbirth in our hospital.

A literature search was performed and audit tools from various organizations were reviewed. A consultant obstetrician and midwife agreed the data points, using the National Clinical Guidelines and the National Bereavement Standards for reference. The audit tool was developed by a software engineer. Data were entered via a secure web form interface and charts were automatically generated and updated as new data were entered.

We report findings of the first forty charts audited. With the exception of two mothers (2/40; 5%) all deliveries occurred in the delivery suite/theatre setting. Infant birth weights ranged between 210 to 5400 grams. A plan of care was documented in 97.5% (39/40) of hospital charts when a stillbirth diagnosis was made and the infant’s name was recorded in all obstetric notes with the exception of one (1/40; 2.5%). In 85% (34/40) of cases mother’s vital sign documentation was recorded, but in 37.5% (15/40) there was no evidence of lactation care recorded. Only 20% (8/40) of obstetric charts had a fully completed stillbirth checklist which is local hospital policy for the multi-disciplinary team. Of those reviewed, 45% (18/40) had a post mortem. Contact with the community professionals was inconsistent, with 75% of general practitioners and only 62.5% of public nurses having documented contact from the hospital after stillbirth.

It is crucial that care around the time of stillbirth is reviewed to ensure that parents receive both meaningful and evidence-based healthcare, enabling them to recover from this life-altering event.

### P82 Development of an electronic audit tool to review quality of care in stillbirth

#### Claire M. Everard^1,2^, Simon Long^3^, Keelin O’ Donoghue^1,2,4^

##### ^1^Pregnancy Loss Research Group, Department of Obstetrics and Gynaecology, University College Cork, Cork, Ireland; ^2^Cork University Maternity Hospital, Cork, Ireland; ^3^Department of Computer Science, Cork Institute of Technology, Cork, Ireland; ^4^Irish Centre for Fetal and Neonatal Translational Research (INFANT), Cork, Ireland

###### **Correspondence:** Claire M. Everard

Stillbirth is a devastating clinical outcome of pregnancy for parents and healthcare staff. Research has shown that poor and uncoordinated care affects parents’ recovery and impacts future pregnancies. It is vital for maternity units to review the quality of care provided for bereaved parents. Audit is a valuable tool to determine whether a unit is giving good care and support to bereaved parents, and identifies possible improvements. Our aim was to develop a customised audit tool for review of the quality of care provided to parents who experience stillbirth.

A literature search was performed and audit tools from various organizations were reviewed. A consultant obstetrician and midwife agreed the data points, using the National Clinical Guidelines and the National Bereavement Standards for reference. The audit tool was developed by a software engineer, and loaded onto a tablet PC. Data was entered via a secure web form interface and charts were automatically generated and updated as new data was entered.

In a pilot study, obstetric charts were inputted into the audit tool, and some changes made to the format. Next, the audit tool was peer-reviewed by the hospital bereavement team and the Pregnancy Loss Research Group. The final customised audit instrument consists of 76 questions designed to record the management of bereaved parents during stillbirth diagnosis, delivery, investigation and follow–up. It takes approximately one hour to complete each chart on the audit tool.

This new audit tool captures a large amount of information about the quality of care which parents bereaved by stillbirth receive. The next phase of this audit instrument is to input the data for CUMH stillbirths from 2008 to 2016. If this audit tool provides valid, accurate and user-friendly data, it will be considered for national use and integration into the electronic health record.

### P83 The challenge of classifying the unexplained

#### Anna Maria Verling^1,2^, Indra San Lazaro^1,3^, Sarah Meaney^1,3^, Keelin O’Donoghue^1,2,4^

##### ^1^Pregnancy Loss Research Group, Department of Obstetrics & Gynaecology, University College Cork, Cork, Ireland, Cork, Ireland; ^2^Cork University Maternity Hospital, Cork, Ireland; ^3^National Perinatal Epidemiology Centre, University College Cork, Cork, Ireland; ^4^Irish Centre for Fetal and Neonatal Translational Research (INFANT), University College Cork, Cork, Ireland

###### **Correspondence:** Anna Maria Verling

Stillbirth is one of the most common adverse pregnancy outcomes. Classification of stillbirth is an essential component of clinical practice and crucial for stillbirth prevention. There are over 30 systems available for perinatal mortality classification globally. No one system has been adopted as a standard, which makes international comparison impossible. This study aimed to evaluate concordance between a selection of international stillbirth classification systems.

All stillbirths between 2008 and 2015 (n = 298) were identified following a retrospective chart review. Eight were excluded due to insufficient data. Cause of death was assigned according to five classification systems; National Perinatal Epidemiology Centre Classification (NPEC); Wigglesworth; Tulip; Relevant Conditions at Death (ReCoDe); Cause of death and associated conditions (Codac).

With the exception of Wigglesworth, the most common cause of death in the remaining four classification systems was from placental causes (28.6%, 39.7%, 36.2%, and 36.3%). In all classifications systems the percentage of stillbirths from congenital anomaly was approximately 24%. Using Wigglesworth, two thirds of stillbirths were unexplained or unclassifiable (62.8%; n = 182); this is significantly higher than the other four systems (Tulip 21.7%, ReCoDe 22.1% and Codac 23.1%) with NPEC having the lowest percentage of these cases (20%; n = 58). Of the 182 cases of unexplained stillbirth classified by Wigglesworth, between 45.8% and 61.6% were identified as having a cause of death related to the placenta within the other four systems.

The value of any classification system is in its ability to identify cause of death and determine contributing factors, however our findings show that at least half of the cases identified as unexplained in Wigglesworth can be attributed to placental conditions within the other four systems. A classification system with low rates of ‘unexplained’ stillbirth is imperative to identify and develop preventative policies and guidelines in maternity care.

Ethical approval for the study was granted by Clinical Research Ethics Committee of the Cork Teaching Hospitals (Reference No: ECM 6(aa) 06/01/15).

### P84 Developing and implementing the national Perinatal Mortality Review Tool (PMRT) for the UK

#### Jenny Kurinczuk^1^, Elizabeth Draper^2^, David Field^2^, Marian Knight^1^, Charlotte Bevan^3^, Thomas Boby^1^, Peter Brocklehurst^4^, Ron Gray^1^, Sara Kenyon^4^, Brad Manktelow^2^, Janet Scott^3^, Judy Shakespeare^1^, Lucy Smith^2^, Peter Smith^1^, Derek Tuffnell^5^, Hannah Knight^6^, Alan Cameron^6^, Zarko Alfirevic^6^, Mandy Forrester^7^, Karen Luyt^8^, Alexander Heazell^9^, Dimitrios Siassakos^8^, Claire Storey^8^, Tracey Johnston^10^

##### ^1^National Perinatal Epidemiology Unit, University Of Oxford, Oxford, UK; ^2^University of Leicester, Leicester, UK; ^3^Sands, London, UK; ^4^University of Birmingham, Birmingham, UK; ^5^Bradford Teaching Hospitals NHS Foundation Trust, Bradford, UK; ^6^Royal College of Obstetricians and Gynaecologists, London, UK; ^7^Royal College of Midwives, London, UK; ^8^University of Bristol, Bristol, UK; ^9^University of Manchester, Manchester, UK; ^10^Birmingham Women’s Hospital, Birmingham, UK

###### **Correspondence:** Jenny Kurinczuk

Parents report that in many instances when a stillbirth occurs a local, structured review of the care provided is not conducted and this is confirmed by evidence from national confidential enquiries. As a consequence parents are not always given a meaningful explanation of why their baby died and the opportunity for service improvement is missed. Based on the work led by the Department of Health and Sands we have been commissioned to develop and implement the national Perinatal Mortality Review Tool (PMRT). The goal of the PMRT is to provide a structure to the process of local perinatal death review thereby improving the quality of the review, maximising learning, and improving service delivery and information for bereaved parents.

The PMRT will be integrated within the MBRRACE-UK system which collects surveillance data about all perinatal deaths in the UK. The tool is based on the principle ‘review once, review well’ by guiding the local multi-disciplinary team through a structured process which considers all aspects of care from pre-conception through to bereavement care and parent follow-up. The views of parents are being incorporated based on evidence from the PARENTS study.

The PMRT has been scoped by a multi-disciplinary team and software development is underway to incorporate the tool into the MBRRACE-UK system. User engagement will guide development at all stages as the tool is iteratively developed and released. Alongside the tool we are developing training materials to support the conduct of high quality reviews and how to use the PMRT as part of the review process. Piloting is in planning with roll out anticipated by the end of 2017.

The PMRT has the potential to improve information for parents, as well as leading to service improvements and consequently reduce the stillbirth rate in the UK.

### P85 Care of families pregnant after stillbirth: results of an international consensus statement

#### Noor Niyar N. Ladhani^1,5,6^, Megan E. Fockler^1,4^, Louise Stephens^2^, Jon F. R. Barrett^1,5,6^, Alexander Heazell^3^

##### ^1^Women and Babies Program, Sunnybrook Health Sciences Centre, Toronto, Canada; ^2^Central Manchester University Hospitals NHS Trust, Manchester, UK; ^3^Tommy’s Stillbirth Research Centre and Maternal and Fetal Health Research Centre, Central Manchester University Hospitals NHS Trust, Manchester, UK; ^4^Lawrence S. Bloomberg Faculty of Nursing, University of Toronto, Toronto, Canada; ^5^Division of Maternal–Fetal Medicine, Department of Obstetrics and Gynaecology, University of Toronto, Toronto, Canada; ^6^Maternal Fetal Medicine, Sunnybrook Health Science Centre, Toronto, Canada

###### **Correspondence:** Noor Niyar N. Ladhani

Stillbirth has a pervasive impact on families, including during subsequent pregnancies. A current lack of clear care pathways for healthcare providers means that care provided varies widely and that many families do not receive coordinated, compassionate, and knowledgeable services. There is increasing evidence that families are not satisfied with existing care pathways and that specialized care is needed during pregnancies after stillbirth.

In October 2015, a consensus meeting was held in Canada. The meeting was a platform to discuss the current evidence, management, and challenges related to pregnancy care after stillbirth internationally, and an opportunity to develop a consensus statement on care of women and their families in pregnancies subsequent to stillbirth.

Representation from nine countries and several specialties were present, including Maternal Fetal Medicine, Obstetrics, Midwifery, Nursing, and Social Work. The meeting also included parent representation from perinatal loss organizations. Recommendations were developed for six priority areas, including:

1. Screening and Investigations - e.g. role of prenatal screening and antiphospholipid antibody testing
2. Medical Therapies – e.g. role of aspirin and anticoagulation
3. Monitoring in Pregnancy – e.g. regimens for monitoring fetal growth
4. Mode/Timing of Delivery – e.g. indications for early term delivery and mode of delivery
5. Psychosocial Care Considerations – e.g. family-centred care and specialist-led clinics
6. Other Considerations – e.g. stillbirth definitions and international considerations

The consensus statement provides guidance for care providers caring for families in pregnancies after stillbirth and will be endorsed by medical, nursing, allied health, and perinatal loss organizations.

Pregnancy after stillbirth presents unique challenges for families and care providers. Optimized medical surveillance, interventions, and psychosocial support by knowledgeable healthcare providers are crucial. The consensus statement findings represent the group’s collective analysis, evaluation, and expert opinion on the best possible way to care for women and their families in subsequent pregnancies.

### P86 Risk factors for stillbirth

#### Jennani Magandran^1,2^, Emily O’Connor^1,2^, Sarah Meaney^1,3^, Anna Maria Verling^1,2^, Noirin E Russell^1,2^, Keelin O’Donoghue^1,2,3,4^

##### ^1^Pregnancy Loss Research Group, Cork, Ireland; ^2^Cork University Maternity Hospital, Cork, Ireland; ^3^National Perinatal Epidemiology Centre, Cork, Ireland; ^4^Irish Centre for Fetal and Neonatal Translational Research (INFANT), Cork, Ireland

###### **Correspondence:** Jennani Magandran

In Ireland, the stillbirth rate is 4.5 per 1,000 births. Knowledge of stillbirth may be affected by personal or clinical experience as well as provision or absence of dedicated staff training and education. This study sought to determine the background knowledge of stillbirth among healthcare professionals.

A cross-sectional survey examining knowledge of stillbirth risk factors in healthcare professionals working in a large, tertiary-level maternity unit with 392 midwifery and 54 medical staff was performed. A detailed questionnaire on stillbirth risk factors was distributed to a random sample of staff members. Descriptive analysis was performed using SPSS v23.

Two hundred and twelve surveys were completed by a cohort of 167 midwives (79%) and 45 doctors (21%). Of these, 93.4% were female (n = 198). Half of participants correctly identified the rate of stillbirth and 62.3% correctly identified the definition of stillbirth in Ireland. Forty-four per cent had attended a stillbirth delivery. Questions and statements regarding risk factors for stillbirth are summarised in Table 12. The most common correctly identified risk factors for stillbirth were smoking (98%), pre-existing hypertension (91.7%), obstetric cholestasis (89.4%), previous history of stillbirth (84.7%), BMI > 30 (82%), recurrent pregnancy loss (76.2%) and maternal age > 35 (75.5%). Less well recognised risk factors included previous caesarean section (29.6%) and maternal influenza A infection (35.5%)

Although many risk factors were correctly identified, there was a lot of variation in knowledge and awareness of established stillbirth risk factors among midwives and doctors in our centre. Many respondents were uncertain regarding the relevance of risk factors and left questions unanswered. Dedicated education for healthcare professionals regarding stillbirth risk factors is essential to optimise patient care.

Ethical approval for the study was granted by Clinical Research Ethics Committee of the Cork Teaching Hospitals (Reference No: ECM 6(aa) 06/01/15).

**Table 12 (abstract P86).** Correct identification of known risk factors for stillbirth and answers regarding statements on stillbirth in 212 Irish healthcare professionals

**Risk factors for Stillbirth**	**True (%)**	**False (%)**	**Don’t know (%)**	**Unanswered (%)**
Previous caesarean section delivery	**59 (29.6)**	125 (59)	15 (7)	13 (6.1)
Previous stillbirth	**172 (84.7)**	23 (10.8)	8 (3.8)	15 (7.1)
Recurrent pregnancy loss	**154 (76.2)**	37 (17.5)	10 (4.7)	9 (4.2)
Obstetric cholestasis	**185 (89.4)**	14 (6.6)	8 (3.8)	5 (2.4)
Influenza A	**70 (35.5)**	67 (31.6)	60 (28.3)	15 (7.1)
Maternal smoking	**202 (98)**	3 (1.4)	1 (0.5)	6 (2.8)
Pre-existing hypertension	**187 (91.7)**	9 (4.2)	8 (3.8)	8 (3.8)
Maternal age over 35 years	**154 (75.5)**	41 (19.3)	9 (4.2)	8 (3.8)
BMI over 30	**140 (82.4)**	17 (8)	13 (6.1)	42 (19.8)
**True statements regarding stillbirth**				
Maternal age >40 doubles risk	**132 (62.3)**	35 (16.5)	35 (16.5)	10 (4.7)
Sleeping in right lateral position increases risk	**24 (11.3)**	154 (72.6)	30 (14.2)	4 (1.8)
Multiple pregnancy increases risk	**140 (66)**	44 (20.8)	20 (9.4)	8 (3.8)
Black women have 4-5 times higher risk than Caucasian women	**45 (21.2)**	78 (36.8)	82 (38.7)	7 (3.3)
Down syndrome increases risk of stillbirth at term	**84 (39.6)**	89 (42)	31 (14.6)	8 (3.8)
Primiparity doubles the risk	**28 (13.2)**	137 (64.6)	35 (16.5)	12 (5.6)
Risk increased beyond 39 weeks in primigravida > 40 years old	**136 (64.2)**	55 (25.9)	16 (7.5)	5 (2.4)
Inter-pregnancy increase of BMI by 5units	**89 (42)**	47 (22.2)	68 (32)	8 (3.8)
**False statements regarding stillbirth**				
Risk with obstetric cholestasis 10%	116 (54.7)	**37 (17.5)**	52 (24.5)	7 (3.3)
IUGR is a factor in 40% of cases	166 (78.3)	**16 (7.5)**	23 (10.8)	7 (3.3)
80% of stillbirth occurs in high-risk pregnancies	92 (43.4)	**89 (42)**	24 (11.3)	7 (3.3)
40% of stillbirths at term due to placental abruption	117 (55.2)	**50 (23.6)**	40 (18.9)	5 (2.4)
Commonest cause of stillbirth 24-28 weeks is infection	112 (52.8)	**42 (19.8)**	50 (23.6)	8 (3.8)
Recreational cannabis use increases risk by 20%	119 (56.1)	**32 (15.1)**	56 (26.4)	5 (2.4)
Equal risk between GDM and IDDM patients	52 (24.5)	**117 (55.2)**	38 (17.9)	5 (2.4)

### P87 Process of developing guidelines for health professionals who support parents who have had a loss from a multiple pregnancy

#### Judith Rankin^1^, Louise Hayes^1^, Judy Richards^1^, Lisa Crowe^1^, Claire Campbell^2^, Nicholas Embleton^2^

##### ^1^Newcastle University, Newcastle upon Tyne, UK; ^2^Newcastle upon Tyne Hospitals NHS Foundation Trust, Newcastle upon Tyne, UK

###### **Correspondence:** Judith Rankin

Parents who lose one baby from a multiple pregnancy experience mixed emotions of enormous grief for the baby who died along with hope and joy at the birth of their surviving baby. In our qualitative work, staff reported that they felt they lacked confidence in supporting bereaved parents in this situation. We describe here the development of guidelines for health professionals involved in supporting parents after the loss of a baby from a multiple pregnancy.

We worked with health professionals and parents to develop the guidelines using a co-design approach. Transcripts of interviews from our previous study were reviewed to identify ‘training points’, factors that were important in determining whether or not parents felt supported by health professionals at the time of their bereavement, and areas where health professionals felt they lacked confidence. Draft guidelines were circulated to health professionals, patient representatives and representatives from relevant patient organisations. These individuals were then invited to attend a workshop to discuss the draft guidelines and agree changes. The final version of the guidelines has been disseminated.

We identified behaviours and actions that staff can adopt that parents find helpful around the time of a bereavement which were incorporated in the guidelines: Recognising that the pregnancy is a multiple pregnancy; Acknowledging the bereavement; Providing emotional support to parents; Providing appropriate information to parents; Providing as much continuity as possible; Offering memory making; Handling cot occupancy sensitively; Preparing parents for discharge from hospital.

Health professionals often lack confidence in supporting parents who have experienced a bereavement from a multiple pregnancy. We worked with health professionals and parents to develop guidelines for staff working in this area. The guidelines will be revised in response to feedback on their use and we will assess their impact on staff knowledge and confidence.

### P88 Stillbirth in Ireland, 2015 – a national clinical audit into mortality due to stillbirth

#### Irene O’Farrell, Paul Corcoran, Edel Manning, Sarah Meaney, Linda Drummond, Paulette deFoubert, Richard Greene

##### National Perinatal Epidemiology Centre, UCC, Cork, Ireland

###### **Correspondence:** Irene O’Farrell

Mortality due to stillbirth is a significant measurement of the outcome of obstetric care. For this reason, in 2011, the National Perinatal Epidemiology Centre (NPEC) established the first national clinical audit of stillbirth in Ireland.

Anonymised data on stillbirths that occurred between January 1 and 31 December 2015 were collected by contributors from each of the 19 maternity units in Ireland using a validated and standardised notification form. National rates per 1,000 births and corresponding 95% confidence intervals were calculated.

In total in 2015, 294 stillbirths were reported to the NPEC, representing a rate 4.5 per 1,000 births. The most common causes of death in stillbirths were major congenital anomaly (n = 79, 27%) and placental conditions (n = 71, 24%). Antepartum deaths accounted for 270 of stillbirths (92%) and intrapartum deaths accounted for 18 (6%), the pre-delivery life status of the baby was unknown for 6 stillbirths (2.0%). Labour was induced for over two-thirds of women who experienced antepartum stillbirth (n = 178, 67%; unknown for 6 cases) whereas labour was spontaneous for 17% (n = 45). Vaginal cephalic delivery was the most common mode of delivery (n = 160, 59%) for women experiencing antepartum stillbirth.

Robust clinical audit of perinatal outcomes are vital for monitoring and improving patient care. The establishment of a confidential enquiry for deaths due to stillbirth should be considered in order to enhance the lessons which may improve care. Anonymised placental histology reports on perinatal death should be submitted to the NPEC as part of this audit: this would facilitate standardised interpretation and classification of placental conditions.

Ethical approval for the NPEC national clinical audits of obstetric outcomes was provided by the Clinical Research Ethics Committee of the Cork Teaching Hospitals (Reference: ECM 4(g) 05/08/08)

### P89 Incorporation of vasa praevia screening into a routine anomaly scan

#### Elizabeth Daly- Jones^1^, Lisa Story^2^, Alexandra Drought^1^, Ciara Mckenna^3^, Philippe De - Rosnay^1^, Millicent Nwandison^1^, Neil Sebire^4^, Dr Philippe Jeanty^5^, David Nyberg^3^

##### ^1^West Middlesex University Hospital, West London, UK; ^2^St Thomas’ Hospital, London, UK; ^3^The Medical Chambers Kensington, London, UK; ^4^Editor of The fetus.net, Nashville, Tennessee, USA; ^5^Great Ormond Street Hospital, London, UK

###### **Correspondence:** Elizabeth Daly- Jones; Lisa Story

Vasa praevia (VP) has a reported fetal mortality of 60% if not recognized before attempt at vaginal delivery. Prenatal detection by ultrasound is possible in nearly all cases, but can be notoriously difficult unless there is a high index of suspicion. We report our experience in detection of VP using a structured protocol as part of the routine fetal anomaly scan at 20-22 weeks

Patients attending anomaly screening at a single centre over a 5 year period (2012-2016) underwent assessment for VP using a structured protocol as a part of a routine fetal anatomy scan. Suspected cases of VP were then re-scanned by a specialist sonographer and subsequently followed up. The diagnosis of VP was assigned by documentation of fetal vessels beneath the membranes, within 20 mm of the internal cervical os.

24690 anatomy scans were performed during the study period and 56 of these were identified as potential VP at the 20 -22 week anomaly scan. Of these, 20 were confirmed by the ultrasound specialist at 28 weeks or later. In five patients the only risk factor was a velamentous insertion. All 20 patients had planned caesarean delivery but one patient bled at 35 weeks before planned delivery and this fetus died. Another patient experienced bleeding and an emergency caesarean delivery at 31 weeks 3 days, and this baby lived. The remaining 18 had caesarean delivery as planned. Placental histology was available in 11 cases. No case of VP was unexpectedly found at delivery during the study period (100% detection).

The incidence of vasa praevia in this unselected population is 1:1234. Fetal mortality was 5% for cases detected prenatally. It is therefore recommended that routine assessment should be incorporated into routine anomaly screening using a structured protocol similar to the one presented here.

### P90 Still birth saga: trends of etiology over a period of ten years in a tertiary care hospital in India

#### Neelam Aggarwal

##### N aggarwal, Pgimer, Chandigarh, India

Although India shares the highest magnitude of still births among all south east Asian countries but as per NHS -4 there is marked achievement in health status of women. We studied causes of all still births(SBs) who delivered in our institute over last ten years and analyzed the trends.

Objective:

To analyze the etiology of still births and their changing trend over a period of 10 years period.

This is a retrospective cohort study of all still birth delivered in a tertiary care referral institute (PGIMER) of northern India over ten year period from 2007 to 2016. The data was collected from monthly and annual perinatal audits and causes of stillbirths along with details of each case were systematically reviewed. Cusick test for trend was used to analyze the changing trends over these years.

There were 54160 total births during study period and out of these 3620 were SBs. The still birth rate ranged from 62 to 73.6 per 1000 total births. The major causes of still births were hypertensive disorders (27.58%), antepartum hemorrhage (19.5%), and congenital anomalies (9.34%). Other causes were poor maternal condition (7.98%), unexplained (7.75%), intrapartum still births (3.55%), rupture uterus (2.29%).Among all still births 53.4% of women had intrauterine fetal death before reaching the hospital.The autopsy rate has raised over years and it was 42% in 2016.

There was no significant change in trends of still birth rate over the years. Even the etiology of still births is almost same over years which may be attributed to being a referral institute.Hypertensive disorder of pregnancy and related complications remained the single most major preventable cause of still birth

### P91 Association of posttraumatic stress following stillbirth with being blamed for the stillbirth, as mediated by maternal age and in-hospital support

#### Allison Badgley, Lauren Christiansen-Lindquist, Carol J. Rowland Hogue

##### Emory University, Atlanta, GA, USA

###### **Correspondence:** Lauren Christiansen-Lindquist

About 1 in 200 pregnancies in the United States end in stillbirth, resulting in substantial psychological morbidities for bereaved mothers and their families. Factors occurring during and immediately after the stillbirth have been reported to have the biggest impact on the later development of posttraumatic stress (PTS) symptoms in bereaved mothers, highlighting the importance of support received in the hospital.

**Fig. 13 (abstract P91).**
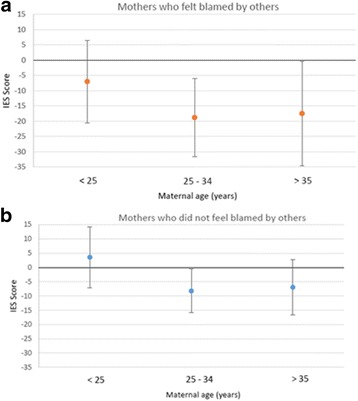
**a** Adjusted* mean change in IES score** attributable to reporting in-hospital support among women who felt blamed others for their stillbirth. **b** Adjusted* mean change in IES score ** attributable to reporting in-hospital support among women who did not feel blamed by others for their stillbirth. *Adjusted for: maternal age, education, marital status, trait anger, time since loss, and interaction of in-hospital support with age and blame. **Impact of Event (IES) Scores <zero indicate fewer post-traumatic stress symptoms than the mean for the group

We investigated the relationship and possible mediators between maternally reported support received from hospital staff and PTS symptoms (measured with the Impact of Events Scale [IES], range 0-75), from SCRN-OASIS follow-up maternal interviews of a sample (n = 254) of mothers in the Stillbirth Collaborative Research Network study who had a stillbirth 6 months to 3 years prior to follow-up. At the follow-up interview, women were asked about events surrounding their earlier loss. Multivariable generalized linear models were weighted by propensity scores for loss to follow-up and included potential confounders and interactions (see list in accompanying Fig. 13).

The average IES score for participants reporting no in-hospital staff support was 31.4 (std = 17.5), compared to 27.1 (std = 16.1) among those reporting support from nurses and other hospital staff (p = 0.111). However, PTS symptoms and associations with support varied by age at index event and blame status. The support/PTS association was not significant among women aged under 25, but was significant for women 35 or older who reported having felt blamed for their loss. Hospital staff support was significantly associated with lower PTS symptoms in all women aged 25-34, especially among those who reported having felt blamed by others (see accompanying Fig. 13).

Receiving support in the hospital from nurses and other staff may be especially effective in preventing post-traumatic stress among vulnerable populations of bereaved mothers who may feel blamed for their loss, particularly if they are 25 or older.

The SCRN-Outcomes after Study Index Stillbirth (OASIS) study was approved by Institutional Review Boards at all participating sites, including Emory University (Study 763-2005 (IRB00000764)). Women with stillbirth who had participated in the original SCRN case-control study were contacted 6 months to 3 years after index delivery if they had provided written consent for further contact.

### P92 Prevalence of maternal disseminated intravascular coagulation after intrauterine fetal death and correlation with maceration grade - a retrospective cohort study

#### Dana Muin, Vanessa Koller, Helmuth Haslacher, Herbert Kiss, Anke Scharrer, Alex Farr

##### Medical University Of Vienna, Vienna, Austria

###### **Correspondence:** Dana Muin

Disseminated intravascular coagulation (DIC) is a clinicopathological syndrome characterised by generation of fibrin clots and concomitant consumption of platelets and coagulation factors, leading to organ failure and contributes to high mortality, if left untreated.

Obstetrical conditions associated with DIC include maternal septic shock, placental abruption, amniotic fluid embolism, as well as preeclampsia and HELLP and acute fatty liver. Fetal maceration has first been proposed as a trigger for DIC in 1901, when DeLee reported about “temporary haemophilia” in a woman who had developed a bleeding disorder after placental abruption and stillbirth of a severely macerated fetus.

The present study was designed to retrospectively evaluate the incidence of coagulopathy in a study cohort over 14 years and correlate the findings with fetal maceration grade using three different DIC-scoring systems.

Coagulation test results (platelet count, fibrinogen, D-Dimer, prothrombin time) were reviewed, scored and correlated with fetal maceration grade

Ten (10.2%) women suffered minor postpartum haemorrhage (PPH), three (3.0%) women suffered major PPH, two of which had clinically verified overt DIC (maceration grades I in both cases). Erez-Score resulted in DIC (score ≥26) in ten (10%) women, Clark-Score resulted in DIC (score ≥3) in four (4%) women and ISTH-score resulted in DIC (score ≥5) in three (3%) women. All women (n = 2; 2%) in the study population with documented overt DIC were correctly identified by all three scores. Erez-Score showed a statistically significant correlation between women tested positive for DIC (score ≥ 26) and fetal maceration grades 0 and 1 compared to fetal maceration grades 2-3 (p <0.05).

Combination of three DIC-scores (ISTH, Erez et al., Clark et al.) achieve the highest sensitivity in detecting women with clinically overt DIC. Low fetal maceration grade is significantly correlated with overt and non-overt DIC test-results by the Erez-Score.

Ethical approval for the study was granted by Medical Research Ethics Committee of the Medical University of Vienna, Austria (Reference Number 1231/2017). Written informed consent was obtained by all study participants.

### P93 Understanding patient satisfaction as a measure of quality in perinatal bereavement care in Spanish hospitals

#### Paul Cassidy^1,2^

##### ^1^University Complutense Madrid, Madrid, Spain; ^2^Umamanita, Madrid, Spain

The objective of the study was to identify the aspects of care that had the greatest predictive power over patient satisfaction.

The study used a cross-sectional descriptive design with an online questionnaire, which included a series of objective measures of care (postmortem contact, mementoes, terminology, birthing method, pathology studies, negligence, etc.) and subjective evaluations of care quality using a battery of statements, measured on a Likert scale. The study included women who had experienced fetal death ≥16 weeks gestation (stillbirth or termination of pregnancy) and within 2 years prior to participation in the study. Based on a series of theoretical considerations related to grief and care, multiple sequential (forward method) regression was used to assess the ability of IVs (objective and subjective) to predict satisfaction.

Responses from 615 women were analysed. Following appropriate testing for multicollinearity and singularity, a significant regression equation was found for both sets of IVs (objective and subjective). The final combined equation (F(8, 581) = 213.701, *p* <0.001) had an R^2^ score that explained 74.9% of the variance (see Table 13). 7 subjective variables were retained by the model, of which the 4 that made the strongest contribution to satisfaction were: ‘feeling that HPs listened’, ‘doctors and nurses working well as a team’, ‘being well-informed about procedures’, and ‘being emotionally supported by nurses/midwives’. Only ‘incidence of reported or perceived negligence’ was retained from the set of objective variables.

The results suggest that satisfaction with perinatal bereavement care is a complex construct, whereby listening skills, coordination of care and effective information permit women to feel in control of decisions. The dominance of subjective measures suggests that satisfaction had more to do with the way that outcomes (e.g. having postmortem contact, keeping linking objects, having an autopsy conducted, etc.) were arrived at than the outcomes themselves.

Ethics approval for the project was not required from the institution for non-clinical studies, however data was collected within an ethical framework.

**Table 13 (abstract P93).** Final regression model summary

				**Change statistics**			
	Independent variables*	**R** ^**2**^	**Std. Error of the Estimate**	**R Square Change**	**F Change**	**Sig. F Change**	**Stand.** **co-eff. Beta**
1	I felt that the professionals listen to me	.585	.855	.585	817.328	.000	.223
2	The doctors, nurses and midwives seemed to work well as a team	.664	.770	.079	135.874	.000	.189
3	In general they kept me/us well-informed of all steps and procedures	.699	.730	.035	67.293	.000	.159
4	Incidence of ‘reported’ or ‘perceived’ negligence**	.720	.704	.021	43.840	.000	.166
5	I felt emotionally supported by the midwives/nurses	.736	.685	.016	34.150	.000	-.134
6	I felt that I had control over decisions related to medical aspects of care (type of birth, medication)	.743	.676	.007	15.032	.000	.093
7	Even though I lost my baby, they (professionals) treated me like a mother	.747	.671	.004	9.798	.002	.087
8	There was one professional who guided me/us through the whole process	.749	.669	.002	4.837	.028	.062

*n = 582*

*Method: Forward*

*Durbin-Watson: 2.024*

*(F(8, 581) = 213.701, p <0.001)*

*Translated from Spanish by the author

**2.5% of respondents stated that they made a claim due to negligence and a further 23.3% replied that they believed there to be negligence, but didn’t report it (n = 608/MR = 7)

### P94 The exploration of the emotional impact of perinatal bereavement support on midwives caring for grieving parents

#### Felicity Agwu Kalu

##### Rotunda Hospital, Dublin, Ireland

Midwives and other healthcare professional are expected to respond appropriately to the needs and expectations of grieving parents around the time of loss and in subsequent pregnancies after the loss to enable them to cope with their loss. However, the provision of effective bereavement support is challenging for some midwives because midwives not only have to provide significant and varied amount of emotional support to bereaved parents but also have to cope with their own emotional responses to the grief of parents.

Aim of the study

To explore the emotional impact of perinatal bereavement support on the midwives caring for grieving parents.

The objectives of the study were:

To identify the factors that affect emotional well being of midwives caring for bereaved parents

To identify emotional support needs of midwives to promote their abilities to provide effective support to grieving parents

Data were collected through a structured questionnaire from three maternity hospitals in Ireland. 268 midwives completed the questionnaire (71% response rate). Ethical approval was received from three maternity hospitals, which were the research sites.

The results of the study showed that the emotional strength of midwives were significantly related to their abilities to provide effective care to grieving parents (N = 268, r = .378, p < .01). However, not all midwives had the emotional strength to remain calm while providing bereavement care.

Midwives are emotionally challenged while providing perinatal bereavement care to grieving parents. Organisations therefore need to provide adequate emotional support and personal development opportunities to midwives caring for bereaved parents to promote their emotional wellbeing and consequent abilities to provide effective care to bereaved parents.

An ethical approval for the study was granted by The Rotunda Hospital, National Maternity Hospital, and Coombe Women & Infants University Hospital Research Ethics Committees (REC-2013-009 & REC-2013-018). This study was an anonymous survey. It was clearly stated on each of the participant’s information leaflet that, by voluntarily completing and returning the questionnaire, the participant was consenting to participate in the research

### P95 Systematic review: what are parents’ and healthcare professionals’ experiences of care after stillbirth in low and middle income countries

#### Clare Shakespeare^1^, Abi Merriel^2^, Danya Bakhbakhi^1^, Claire Storey^3^, Dimitrios Siassakos^2^

##### ^1^North Bristol NHS Trust, Bristol, UK; ^2^University of Bristol, Bristol, UK; ^3^International Stillbirth Alliance, Bristol, UK

###### **Correspondence:** Clare Shakespeare

Stillbirths have a profound impact on women, families, and healthcare workers. The burden is highest in low and middle income countries (LMICs) where an estimated 98% of stillbirths occur. There is need for respectful and supportive care for women, partners and families after bereavement. Previous reviews have focussed mainly on high income, Westernised settings.

A systematic review of quantitative, qualitative and mixed-method studies of parents’ and healthcare professionals’ experiences of care after stillbirth in LMICs was carried out. Studies were screened and data extracted in duplicate. Data was analysed using the Sandelowski meta-summary technique to identify themes which could inform guidelines for health care workers and improve the provision of care.

Studies were identified from Africa, Asia, Latin America and Europe. Emerging themes identified include:

Women want supportive, respectful and empathetic care by skilled health care professionals, with explanation and counselling both during and after delivery. Receiving this can positively influence their experience.

Women may experience feelings of guilt, or feel that they carry much of the emotional burden alone, and may fear negative social consequences and isolation due to their reproductive status. Support by partners, families and the community throughout the process from diagnosis to recovery at home is desired by women.

Spiritual and religious support is important, especially for women who rely on religious, spiritual or supernatural explanations for what has happened to them.

Both staff and patients believe there is a need for specific training in caring for women who experience perinatal loss.

More research is needed in a range of LMICs. Although there will always be cultural differences to consider, there are sufficient similarities with themes identified in reviews from high income settings to consider the development of an international consensus for care after stillbirth for women and families in all settings.

### P96 Risk assessment and management of diabetes in pregnancy and associated stillbirth – an audit in North Bristol

#### Kate Dodd, Katie Cornthwaite, Dimitrios Siassakos

##### North Bristol NHS Trust, Bristol, UK

###### **Correspondence:** Kate Dodd

Diabetes in pregnancy is a key risk factor for stillbirth, with gestational diabetes affecting around 5% of pregnancies in the UK. It can cause fetal complications including macrosomia, polyhydramnios, delivery complications and stillbirth.

Patients are risk-stratified at their booking appointment and those that are high-risk are offered further investigation for diabetes in their pregnancy and increased monitoring for possible complications. Early detection of diabetes is vital for initiation of appropriate management to optimise patients’ antenatal care.

We retrospectively investigated notes of patients who had a stillbirth that delivered at North Bristol NHS Trust between February 2013 and October 2016 using a proforma. We analysed the data using simple statistical tests.

Of 52 notes obtained, 39 were included. One patient had pre-existing diabetes. Sixteen were diagnosed as having definite risk factors for gestational diabetes, whilst a further 12 patients had indeterminate risk factors. Of these, seven patients (43.8%) were offered screening for gestational diabetes and 5 underwent the screening – all of which were negative.

On post-mortem examination, one fetus showed evidence of macrosomia and three showed evidence of diabetes. Of these cases: one mother had one episode of significant glycosuria but no other risk factors for diabetes; one had risk factors for gestational diabetes but not screened; one had no risk factors for diabetes; and the macrosomic fetus was born from the patient with pre-existing diabetes.

Diabetes in pregnancy is a major risk factor for stillbirth and it is fundamental that patients are correctly risk-stratified for diabetes at their booking appointment, with high risk patients receiving appropriate screening and management.

With this project, we aim to enhance the care of women by improving the risk-stratification identification process and arrange appropriate follow-up investigations and management to inform antenatal care and ultimately to reduce the risk of stillbirth.

Ethical approval for the audit was granted by the audit team at North Bristol NHS Trust prior to the project being undertaken

### P97 Saving Babies Lives: risk assessment and surveillance for fetal growth restriction in women who have experienced a stillbirth

#### Kate Dodd, Danya Bakhbakhi, Dimitrios Siassakos

##### North Bristol NHS Trust, Bristol, UK

###### **Correspondence:** Kate Dodd

Around 3600 stillbirths occur in the UK annually - one of the highest rates in high income countries.

In 2014, the ‘Saving Babies Lives’ bundle was developed and supported by NHS England and the Royal Colleges, and developed using MBRRACE reports to improve prevention of stillbirths, including risk assessment and surveillance for fetal growth restriction.

Impaired fetal growth is a major risk factor for stillbirth and detection of growth-restricted fetuses is vital for recognition and appropriate management to prevent stillbirths.

‘Saving Babies Lives’ aims for all women to be risk-stratified for fetal growth restriction, and all high-risk women should have serial growth scans. All low-risk women should be assessed for growth restriction using had symphysis-fundal height (SFH) measurements and referred for further assessment if indicated.

We retrospectively investigated notes of women who had experienced a stillbirth in a hospital in Bristol between February 2013 and October 2016. We used the Saving Babies Lives’ recommendations to identify if women met the criteria for fetal growth surveillance and were managed appropriately.

Of 52 notes obtained, 39 were included. Although all 39 patients had SFH measurements taken, none had measurements plotted on growth charts. Thirty-six patients (92.3%) were suitable for SFH measurements used to measure fetal growth. Twelve patients were deemed high-risk of growth restriction and all had serial growth scans. No patients were mis-straified. Five high-risk patients (12.8%) had evidence of fetal growth restriction, with no low-risk patients showing evidence growth restriction.

Plotted SFH measurements and serial growth scans are fundamental in the detection and management of fetal growth restriction and its impact on stillbirths. We aim to enhance our care of women by improving the documentation of measurements and risk-stratification to reduce the risk of stillbirth by adhering to guidance from the ‘Saving Babies Lives’ bundle.

Ethical approval for the audit was granted by the audit team at North Bristol NHS Trust prior to the project being undertake

### P98 Association between delays in obstetric care and stillbirth: a case-control study

#### Marley Martins^1^, Flavio Ibiapina^2^, Ocilia Carvalho^1^, Antônio Viana Junior^1^, Raimunda Magalhães^2^, Herlânio Carvalho^1^

##### ^1^Maternidade-Escola Assis Chateaubriand – Universidade Federal do Ceará, Fortaleza, Brazil; ^2^University of Fortaleza, Fortaleza, Brazil

###### **Correspondence:** Marley Martins, Flavio Ibiapina

Stillbirth is a sensitive indicator of the quality of and accessibility to health care among pregnant women at all levels of care. This study aims to assess the association between delays in the care provided to pregnant women seeking obstetric care and stillbirth in a reference tertiary maternity hospital in Northeastern Brazil.

Case-control study with 65 participants (35 controls and 30 stillbirths) conducted at the Assis Chateaubriand Maternity Hospital (Federal University of Ceará) from January to April 2017. Live births occuring on a same day were selected as controls. Controls were matched to cases (1 to 1) on gestational age at birth. Five controls refused to participate in the interview. There were 5 intrapartum and 30 antepartum deaths. Pearson’s chi-squared test and Fisher’s exact test were used to compare the groups. Statistical significance was set at p < 0.05.

There were no statistically significant differences between the groups regarding socioeconomic and obstetric variables. The delays assessed were: absence or inadequacy of antenatal care (54.3 × 33.3%, p = 0.074), patient unaware of the signs of risk (20 × 3.3%, p = 0.045), problems with patient transportation (22.9 × 10%, p = 0.148), difficult access for geographic reasons (42.9 × 30%, p = 0.208), delayed diagnosis of obstetric status (28.6 × 6.7%, p = 0.023), lack of trained personnel (31.4 × 6.7%, p = 0.013), delayed initiation of treatment (74.3 × 40%, p = 0.005), regulatory difficulties/referral–counter-referral (17.1 × 23.3%, p = 0.377), lack of resources/infrastructure (45.7 × 20%, p = 0.026).

In the stillbirth group, most delays in obstetric care were related to the patient/family, the health system/infrastructure and the health team.

### P99 Pregnancy after loss: women’s challenges and resources needed

#### Francine Demontigny^1,2^, Chantal Verdon^2^, Emmanuelle Dennie-Fillion^2^

##### ^1^Université Du Québec En Outaouais, Gatineau, Canada; ^2^Center of Studies and Research in Family Intervention, Gatineau, Canada

###### **Correspondence:** Francine Demontigny

From the pre-pregnancy to the postnatal period, future mothers having experienced a previous perinatal bereavement undergo alternating emotions ranging from happiness to fear and anxiety. Whereas some attention has been paid to the needs of mothers during future pregnancies, little has been done to create resources for them. Purpose. Explore mother’s needs in a pregnancy following perinatal bereavement.

A qualitative research was carried out with 8 women in the context of a focus group.

From pregnancy to the postpartum period, women identified various challenges met in regards to the pregnancy. These challenges related to the concepts of conflicting emotions as well as a sense of urgency “making up for lost time”, fear of the unknown, loss of naivety, isolation as well as inadequately trained professionals and resources to help them cope. Following these interviews, a support group for couples experiencing a future pregnancy was set up to meet the needs of this specific population. An online space to promote sharing and peer support was created as well.

Professionals can provide better support for couples experiencing a pregnancy after perinatal bereavement. Recommendations for nursing and midwifery practice, education and future research are identified.

### P100 Abnormal placental cord insertion and risk of adverse pregnancy outcomes: results from a prospective cohort study

#### Khadijah Irfah Ismail^1^, Ailish Hannigan^2^, Keelin O’Donoghue^3^, Amanda Cotter^1^

##### ^1^Obstetrics and Gynaecology, Graduate Entry Medical School, University of Limerick, Limerick, Ireland; ^2^Biostatistics Department, Graduate Entry Medical School, University of Limerick, Limerick, Ireland; ^3^Obstetrics and Gynaecology, University College Cork, Cork, Ireland

###### **Correspondence:** Khadijah Irfah Ismail

Placental cord insertions (PCI) are categorised as central/eccentric (>2cm from placental margin), marginal (<2cm from placental margin) or velamentous (insertion into the membranes), with marginal and velamentous classified as abnormal PCI. A recent meta-analysis suggests an increased risk of intrauterine fetal death (IUFD) with velamentous cord insertion. We examined the association of abnormal PCI with adverse pregnancy outcomes including IUFD.

This prospective cohort study examined 1005 placentas from consecutively delivered singleton pregnancies in a tertiary centre. All the placentas were examined following delivery. Standardised images of each placenta were taken. Distance of PCI to the placental margin was measured digitally using ImageJ software. Outcomes including small for gestational age (SGA) infants (<10th centile), low birthweight (<2500g), preterm labor (<37 weeks gestation), IUFD and emergency cesarean section (CS) were compared across groups (central, abnormal PCI) using logistic regression, adjusting for maternal age, parity and smoking status. Odds ratios (OR) and 95% confidence intervals were estimated using SPSS.

The rates of velamentous and marginal cord insertions in a total of 1,005 singleton pregnancies were 3.6% (n  =  36; 95% CI  =  2.5–4.9%) and 6.4% (n  =  64; 95% CI  =  4.9–8.1%), respectively. Abnormal PCI were found to be significantly associated with an increased risk of SGA after adjusting for maternal age, smoking status and parity (Table 14). Preterm labor was more common in the abnormal PCI group (5.1% vs 10.0%, p = 0.04) but the adjusted OR was not significant (Table 14). There was no difference in emergency CS rate across groups. There was one case of IUFD in the cohort with normal PCI and a non-SGA fetus.

In this large prospective cohort, we were unable to assess the association of abnormal PCI with IUFD. Due to the rarity of IUFD, a much bigger study is needed to assess its association with abnormal PCI.

Ethical approval for the study was granted by Health Service Executive Research Ethics Committee of University Hospital Limerick (REC Reference 32/13). Written informed consent was obtained by all study participants.

**Table 14 (abstract P100).** Maternal Characteristics and pregnancy outcomes across groups (Normal PCI and Abnormal PCI)

	**Normal PCI (n = 905)**	**Abnormal PCI (n = 100)**	**p-value**
Maternal age ≥35 years	332 (36.7%)	30 (30%)	0.19
Parity			
0	331 (36.7%)	39 (39%)	0.33
1	299 (33.1%)	26 (26%)	
2+	272 (30.2%)	35 (35%)	
Smoker	127 (14.8%)	22 (22.7%)	**0.04**
Birth weight <2500g	32 (3.5%)	11 (11.0%)	**<0.001**
SGA infant	120 (13.3%)	21 (21.0%)	**0.035**
Gestational age < 37 weeks	46 (5.1%)	10 (10.0%)	**0.04**
Emergency CS	147 (16.2%)	18 (18.0%)	0.65

### P101 Factors contributing to bereaved parents of stillbirth consent to post-mortem examination and reflections of their decision: a qualitative analysis

#### Erin Mccloskey, Alex Cohen, Hannah Blencowe

##### London School Of Hygiene & Tropical Medicine, London, UK

###### **Correspondence:** Erin Mccloskey

One of the most difficult decisions bereaved parents face with the sudden loss of their child is to decide whether they wish to conduct a post mortem examination (PME) to determine the cause of death. Although PMEs remain the gold standard in revealing a stillbirth’s cause of death, consent rates have decreased. This project explores bereaved parents’ decisions to accept or reject a post mortem examination and their responses related to the results.

This secondary qualitative analysis explores a dataset which 22 parents were interviewed comprising of 25 respondents. Three interviews comprised of both mothers and fathers. Thematic analysis with an essentialist/realist approach was utilized as it identifies and reports patterns from experiences, meanings and reality of the participants within the data set. NVivo10 was used to manage the data.

Ten couples expressed a strong desire to find out the cause of death gave consent to a thorough PME. Parents who gave consent prioritized determining the cause of death by means of genetic testing to confirm they could try for another baby. One couple gave consent to a limited PME, because they felt it wasn’t necessary to have a thorough investigation. Eleven couples refused PMEs because of the lack of support and empathy from staff and time constraints to bury the child. Parents reported psychological distress from panic attacks and depression to more severe mental health issues.

Consent to PME was largely dependent upon the parents’ rapport with healthcare staff and how PME was explained to them. Bereaved parents should not experience inadequate and unsupportive care as it may affect consent rates. Despite how respondents felt about consenting to PME, all parents felt the need to improve stillbirth bereavement policies by educating the public through means of participating in studies to honor their child.

This study is a secondary qualitative analysis. Ethical approval for the study was granted by the University of Manchester Research Ethics Committee (Project, 09392). Written informed consent was obtained by all study participants.

### P102 Investigations undertaken following a stillbirth across Australian hospitals

#### Hanna Reinebrant^1^, Glenn Gardener^1^, David Ellwood^2^, Michael Coory^1^, Kassam Mahomed^3^, Yee Khong^4^, Adrienne Gordon^5^, Alison Kent^6^, Vicki Flenady^1^

##### ^1^Mater Research Institute-University Of Queensland, Brisbane, Australia; ^2^Gold Coast University Hospital, Gold Coast, Australia; ^3^Ipswich Hospital, Ipswich, Australia; ^4^South Australian Pathology, Adelaide, Australia; ^5^Royal Prince Alfred Hospital, Sydney, Australia; ^6^Centenary Hospital for Women and Children, Canberra, Australia

###### **Correspondence:** Hanna Reinebrant

Every day six babies are stillborn in Australia with 30- 50% classified as unexplained. A cornerstone to developing effective intervention strategies to prevent stillbirth is the accurate identification of cause of death based on appropriate investigations. In this study we aimed to determine use of the comprehensive national clinical practice guidelines on investigations for stillbirth.

A prospective multi-centre study of all stillbirths excluding terminations of pregnancy was undertaken across 23 largely tertiary level maternity hospitals. Data were entered by hospital staff using a purpose-built online database and classified according to the Perinatal Society of Australia and New Zealand (PSANZ) system.

541 stillbirths were included; 49% were ≥28 weeks’ gestation and 16% were intrapartum. A comprehensive maternal history was reported as available in 84% and external examination by the clinician in 88%. Testing for feto-maternal haemorrhage was undertaken in 69%.Placental histopathology was undertaken in 94%. While autopsy was offered to parents in 95% of stillbirths, only 51% had an autopsy. Amniocentesis was performed in 19%. 41% of stillbirths remained unexplained.

The recommended stillbirth investigations showed reasonable compliance across tertiary settings in Australia. Although some areas for improvement were shown, including the fundamental component of taking a comprehensive maternal history. Autopsy rates remain low despite the national educational program (IMPROVE), on recommended investigations and information materials for families. The proportion of unexplained stillbirths is high and efforts to improve investigation and classification of stillbirths remains an important issue in Australia.

### P103 How do mobile pregnancy applications address decreased fetal movement as a risk factor for adverse perinatal outcomes and stillbirth?

#### Lisa Daly^1^, Vicki Flenady^1^, Han Le^2^, Janine Roberts^2^, Kirsten Gibbons^1^

##### ^1^Mater Research Institute - The University Of Queensland, Brisbane, Australia; ^2^The University of Queensland, Brisbane, Australia

###### **Correspondence:** Vicki Flenady

Maternal perception of fetal movement is an indicator of fetal wellbeing, while a perceived decrease in fetal movement (DFM) has clinical significance as a predictor of pregnancies at risk of adverse outcomes, including stillbirth. Increasingly, pregnant women are turning to digital sources of health information to build knowledge, track data, share experiences and seek reassurance. Mobile applications intended for use during pregnancy offer information about fetal movement, and may influence behaviour of women experiencing DFM.

A systematic review was conducted to assess how mobile “pregnancy” applications address decreased fetal movement, utilise evidence-based information, and encourage health care-seeking behaviour. The search strategy identified eligible mobile applications, with inclusion criteria based on accessibility, reach, relevance and quality. Two reviewers extracted data from eligible mobile apps using predefined, standardized formats, investigating the content explicitly linking DFM and adverse perinatal health outcomes.

Based on inclusion criteria, 24 mobile applications relevant to pregnancy were included in the review; all were available in English, had more than 100,000 installations, and had high user quality ratings. All applications provided information about DFM. However, explicit linkage of DFM to potential adverse health outcomes was slim: only two mobile applications in the sample explicitly linked DFM to each of the following adverse outcomes: low birthweight, fetal growth restriction, emergency delivery and pre-term birth. Four apps linked DFM to higher risk of stillbirth.

This review is the first to assess information about fetal movement available in the mobile applications used globally by millions of pregnant women. Women experiencing DFM may act more quickly to investigate concerns if aware of potential, related adverse perinatal outcomes. In development of antenatal education, clinicians, hospitals and health systems should consider the content of mobile applications and their contribution to patient knowledge and decision making.

### P104 Is area-based socio-economic deprivation associated with stillbirth in Queensland, Australia? A retrospective population-based study, 1994-2011

#### Susannah Hopkins Leisher^1,2^

##### ^1^Columbia University Mailman School of Public Health; Mater Research Institute, University of Queensland, Brisbane, Australia; ^2^International Stillbirth Alliance, Bristol, UK

About 2.7 million babies are stillborn every year. While 98% occur in low- and middle-income countries, in high income countries huge disparity exists for disadvantaged women. The primary objective of this study was to examine the association between area-based socio-economic deprivation and stillbirth risk. Secondary objectives were to explore changes over time, and association between deprivation and both cause-specific and gestational age-specific stillbirth.

A retrospective population-based study, including singleton births included in the routine birth data set between July 1994 and December 2011 in the Queensland, Australia. Of 928,313 births, 893,648 were included in descriptive analysis and 890,084 in regression analysis.

This study found that greater deprivation is associated with a higher risk of stillbirth. The adjusted risk increased by 5% for every quintile increase in deprivation (22% higher among most-deprived than least-deprived births, and 36% higher among births of at least 28 weeks’ gestation). For every cause and gestational age period, the risk of stillbirth was higher in the most-deprived than the least-deprived quintile. The risk of stillbirth due to perinatal infection, hypertension, and antepartum hemorrhage was more than twice as high among the most-deprived as least-deprived. The gestational age risk was isolated to term births where the risk of stillbirth was increased by 13% for every quintile increase in deprivation. There was also evidence of a decreased risk of stillbirth among most-deprived as compared to least-deprived quintiles over time.

Results suggest that one driver of high stillbirth risk in Queensland is area-based inequity, possibly related to differing access to, demand for, or quality of prenatal services. Improved access to and quality of perinatal services in the most-deprived areas are needed. The higher risk for term stillbirth and stillbirth due to antepartum hemorrhage, perinatal infection, and hypertension should be explored.

The Australian Institute of Health and Welfare Human Research Ethics Committee (HREC) and Queensland Health Central Office HREC approved the original study of which this study forms a part (#EC2009/3/34 and #HREC/05/QHC/009). Specific approval for this study is documented in #HREC/15/MHS/36 and #HREC/15/MHS/36/AM07, and by the London School of Hygiene and Tropical Medicine MSc Research Ethics Committee (#10280). The data was routinely collected and had been de-identified; hence, it was deemed unnecessary to obtain informed consent. However, due to at-risk populations (pregnant women and fetuses), extra precautions were taken. Continuous variables were converted to categorical and continuous versions deleted. Combining variable categories and primary and secondary suppression of data were used to protect the confidentiality of births represented by small (non-zero) counts

### P105 BHRUT Multidisciplinary Strategies to Reduce Stillbirths and Term IUD

#### Celia Burrell

##### Barking Havering and Redbridge University Hospital, Romford, Essex, UK

The UK stillbirth rate fell slightly from 0.54% (2000) to 0.47% (2013), but remains higher than other European countries. UK is ranked 24th of the 50 highest income countries worldwide (Lancet Reducing Stillbirth Series 2016). BHRUT stillbirth rate was 0.59% (2011) falling to 0.4% (2013). BHRUT aims to reduce stillbirth and neonatal death by 50% by 2030 (‘Sign Up To Safety Campaign’ 2014).

Method: To discuss the Bereavement Team and Risk Management Team Multidisciplinary Strategies to reduce stillbirth and term IUD rates.

Results: (1) IMPROVEMENT IN TEACHING -INTRODUCTION OF FETAL LOSS STUDY DAY (2015). Annual multidisciplinary study day teaches staff to improve communication, provide sensitive and supportive care, with feedback and talks from bereaved women.

(2) INTRODUCTION OF CENTRAL MONITORING CTG AND A SPECIALIST CTG MIDWIFE (2016).

Supports midwives and doctors with CTG interpretation during intra-partum and antenatal care. Includes ‘Fresh Eyes’ hourly CTG review in labour, to identify CTG misinterpretation to escalate and expedite delivery.

- REVIEW STILLBIRTH, IUD AND FETAL LOSS >24 WEEKS.

Introduce proforma to review cases, and launch RCA investigation after Serious Incidents Group review. Lessons learnt are widely disseminated.

(3) SERVICE IMPROVEMENT: INTRODUCTION OF CONSULTANT-LED BEREAVEMENT CLINIC (2015). Introduction of new guidelines; improvement in communication with proforma -women submit questions for RCA; review and discuss case notes (investigations -postmortem); practice the duty of candour; provide individualised/tailored management; and empowers women with additional support in subsequent pregnancy.

BHRUT Multidisciplinary strategies have resulted in dramatic improvements:

- No CTG Misinterpretation Related SI or RCA declared past 8 months (August 2016-April 2017);

-New Proforma resulted in greater communication, empowerment and involvement of bereaved women in RCA investigations and management plan in subsequent pregnancy.

(Lessons Shared– Abstract published as Poster Presentation at MBRRACE 2016 & EBC launch 2016)

